# Poecillastrosides, Steroidal Saponins from the Mediterranean Deep-Sea Sponge *Poecillastra compressa* (Bowerbank, 1866)

**DOI:** 10.3390/md15070199

**Published:** 2017-06-26

**Authors:** Kevin Calabro, Elaheh Lotfi Kalahroodi, Daniel Rodrigues, Caridad Díaz, Mercedes de la Cruz, Bastien Cautain, Rémi Laville, Fernando Reyes, Thierry Pérez, Bassam Soussi, Olivier P. Thomas

**Affiliations:** 1 School of Chemistry, National University of Ireland Galway, University Road, H91 TK33 Galway, Ireland; KEVIN.CALABRO@nuigalway.ie; 2 Cosmo International Ingredients, 855 avenue du Docteur Maurice Donat, 06250 Mougins, France; remi.laville@airlquide.com; 3 Géoazur, Université Côte d’Azur, CNRS, OCA, IRD, 250 rue Albert Einstein, 06560 Valbonne, France; elaheh.lotfi-kalahroodi@univ-rennes1.fr (E.L.K.); daniel4rodrigues@gmail.com (D.R.); bassam.soussi@gu.se (B.S.); 4 Institut Méditerranéen de Biodiversité et d’Ecologie marine et continentale, CNRS—Aix-Marseille University, IRD—University Avignon, Station Marine d’Endoume, rue de la batterie des lions, 13007 Marseille, France; thierry.perez@imbe.fr; 5 Fundación MEDINA, Centro de Excelencia en Investigación de Medicamentos Innovadores en Andalucía, Avda. del Conocimiento 34, Parque Tecnológico de Ciencias de la Salud, E-18016 Armilla, Granada, Spain; caridad.diaz@medinaandalucia.es (C.D.); mercedes.delacruz@medinaandalucia.es (M.d.l.C.); bastien.cautain@medinaandalucia.es (B.C.); fernando.reyes@medinaandalucia.es (F.R.); 6 Department of Marine Sciences, University of Gothenburg, P.O. Box 460, SE40530 Gothenburg, Sweden; 7 Oman Centre for Marine Biotechnology, P.O. Box 236, PC 103 Muscat, Oman

**Keywords:** sponge, saponins, deep-sea, *Poecillastra compressa*

## Abstract

The first chemical investigation of the Mediterranean deep-sea sponge *Poecillastra compressa* (Bowerbank, 1866) led to the identification of seven new steroidal saponins named poecillastrosides A–G (**1**–**7**). All saponins feature an oxidized methyl at C-18 into a primary alcohol or a carboxylic acid. While poecillastrosides A–D (**1**–**4**) all contain an *exo* double bond at C-24 of the side-chain and two osidic residues connected at O-2′, poecillastrosides E–G (**5**–**7**) are characterized by a cyclopropane on the side-chain and a connection at O-3′ between both sugar units. The chemical structures were elucidated through extensive spectroscopic analysis (High-Resolution Mass Spectrometry (HRESIMS), 1D and 2D NMR) and the absolute configurations of the sugar residues were assigned after acidic hydrolysis and cysteine derivatization followed by LC-HRMS analyses. Poecillastrosides D and E, bearing a carboxylic acid at C-18, were shown to exhibit antifungal activity against *Aspergillus fumigatus*.

## 1. Introduction

In the marine environment, steroid and triterpenoid glycosides are widespread metabolites mainly produced by echinoderms [1,2,3], although saponins have also been isolated from other marine invertebrates such as octocorals or sponges [4,5]. To date, about 70 saponins have been reported from sponges [6] including sarasinosides from *Asteropus* spp. [7,8], *Melophlus* spp. [9,10], and *Lipastrotethya* sp. [11], ulososides from *Ulosa* sp. [12,13] and *Ectoplyasia ferox* [14], pandarosides and acanthifoliosides from *Pandaros acanthifolium* [15,16,17,18], wondosterols from the association of two sponges [19], erylosides, sokodosides, nobiloside, and formosides from *Erylus* spp. [20,21,22,23,24,25,26,27,28,29], ptilosaponosides from *Ptilocaulis spiculifer* [30], mycalosides from *Mycale laxissima* [31,32], feroxosides from *Ectyoplasia ferox* [33], and silenosides from *Silene vulgaris* [34]. While some sponge saponins can be oxidized on the D ring or can contain unusual side chains, the aglycone of most of them belongs to the 30-norlanostane triterpenoid family, with steroidal saponins being rather rare for sponges. Some sponge saponins were subjected to different bioassays and they usually demonstrated interesting biological activities, mostly cytotoxicity against tumor cell lines [35,36,37].

**Figure 1:**
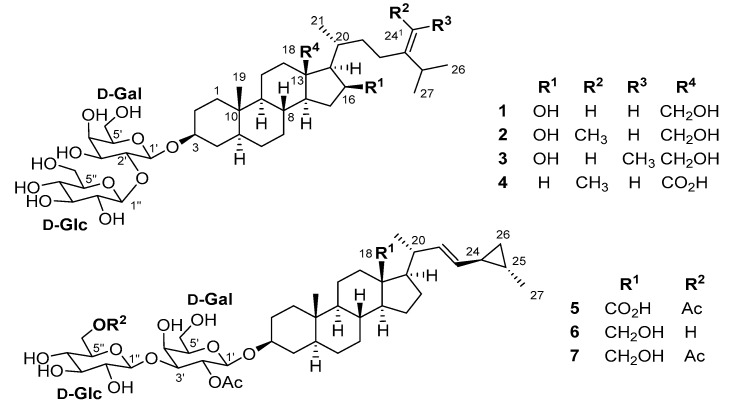
Structure of poecillastrosides A–G (**1**–**7**).

In our continuous efforts to describe the chemical diversity of marine sponges from the Mediterranean, we undertook the first chemical study of the deep-sea Tetractinellid sponge *Poecillastra compressa* (Bowerbank, 1866). The genus *Poecillastra* is known to produce a broad range of secondary metabolites such as macrolactams [38,39], nitrosohydroxyalkylamines [40], sesquiterpenes, and steroids [41,42]. We report herein the isolation and structure elucidation of seven new steroidal glycosides named poecillastrosides A–G (**1**–**7**) from the deep-sea sponge *P. compressa* (Figure 1). Their structures were deduced from spectroscopic data including 1D- and 2D-NMR experiments as well as high-resolution mass spectra (HRESIMS) analyses. Three different aglycone moieties were identified, and oxidation at the C-18 position is a common feature among all isolated saponins. Poecillastroside A (**1**) contains an ergostane aglycone, whereas poecillastrosides B–D (**2**–**4**) contain a poriferastane, and poecillastrosides E–G (**5**–**7**) a cholestane with a cyclopropyl ring on the side-chain.

## 2. Results and Discussion

The freeze-dried sponge sample (43.1 g) was macerated and repeatedly extracted with a mixture of CH_2_Cl_2_/CH_3_OH (1:1) under sonication. The extract (7.9 g) was fractionated by Reversed Phase C18 Vacuum Liquid Chromatography with solvent mixtures of decreasing polarity. The methanolic fraction was then purified by successive RP-Phenylhexyl and C18 HPLC yielding pure compounds **1**–**7**. 

Compound **1** was isolated as a yellowish amorphous solid. Its molecular formula C_40_H_68_O_13_ was determined by HRESIMS. The ^1^H NMR spectrum of **1** suggested a steroidal saponin (Table 1). First, the characteristic anomeric signals at δ_H_ 4.49 (d, *J* = 7.6 Hz, 1H, H-1′), 4.56 (d, *J* = 7.9 Hz, 1H, H-1″), and δ_C_ 101.8 (C-1′), 105.2 (C-1″) evidenced the presence of two sugar residues. The ^1^H NMR data of the steroid revealed one methyl singlet at δ_H_ 0.88 (s, 3H, H_3_-19), three methyl doublets at δ_H_ 1.02 (d, *J* = 6.8 Hz, 3H, H_3_-21) and 1.03 (d, *J* = 6.8 Hz, 6H, H_3_-26 and -27), ten methylene groups, an oxygenated methylene with the AB system at δ_H_ 3.95 and 3.59, a 1,1-disubstituted olefin at δ_H_ 4.70 and 4.71 (H_2_-24^1^), seven methine groups, two oxygenated methines at δ_H_ 3.72 (m, 1H, H-3), 4.26 (td, *J* = 7.7, 3.7 Hz, 1H, H-16), and three quaternary carbons at C-10, C-13 and C-24. When compared to usual steroids, this aglycone lacks one characteristic methyl signal for C-18. A hydroxylation was proposed at this position based on the presence of an AB system at δ_H_ 3.59 (d, *J* = 11.5 Hz, 1H, H-18b) and 3.95 (d, *J* = 11.5 Hz, 1H, H-18a) and further key H-12b, H-14, H-17/C-18, and H_2_-18/C-13, C-14, C-17 HMBC correlations. Another unusual feature for the steroid moiety was evidenced in the HSQC spectrum with signals of an oxygenated methine at δ_H_ 4.26 (td, *J* = 7.7, 3.7 Hz, 1H, H-16) and δ_C_ 72.8 (CH, C-16). The location of this hydroxyl group at C-16 was confirmed after interpretation of key H-16/H-17 and H-16/H-15a COSY and TOCSY correlations. While most of the relative configurations were in accordance with a common steroid core, the relative configuration at C-16 was established after examination of the NOESY spectrum. Absence of clear nuclear Overhauser effect (nOe) between H-16 and H-14 but also H-18 together with some overlap between H-17 and H-22 did not allow a straightforward determination of the relative configuration at this position. However, H-16/H-15a and H-8/H-15b nOes suggested a β orientation for the hydroxyl group at C-16. As a confirmation of this orientation, the coupling constant values of H-16 were in perfect accordance with those observed for the same signal of a closely related analogue weinbergsterol B, isolated from the sponge *Petrosia weinbergi* [43]. NMR signals of the sugar residues were assigned by extensive COSY, TOCSY, and HSQC interpretation. HMBC experiment evidenced H-5′/C-1′, H-1″/C-2′, H-5″/C-1″ long-range correlations, thus revealing the pyranose nature of these two sugars and their connection at C-2′. Finally, the connectivity of the sugar with the aglycone at C-3 was confirmed through the key HMBC H-1′/C-3 correlation. Moving to the relative configuration of the residues, the large coupling constants between H-1′/H-2′ and H-1″/H-2″ (7.9 and 7.6 Hz, respectively) were consistent with a β configuration for both anomeric centers. This interpretation was confirmed with the one-bond coupling constant ^1^*J*_CH_ ≈ 160 Hz for the two anomeric positions [44]. In addition, the coupling constant values of ^3^*J*_H3′–H4′_ 3.2 Hz and ^3^*J*_H5′–H4′_ close to zero suggested an axial position for the hydroxyl at C-4 and, therefore, a β-galactopyranosyl residue attached at C-3 of the aglycone [45]. For the second sugar residue, all coupling constants were measured with values between 7 and 9 Hz which implies equatorial positions for all oxygen atoms and, therefore, a β-glucopyranosyl residue connected at C-2′ of the first residue. 

Assuming a usual absolute configuration for the aglycone, we turned towards the pyranose moieties. After hydrolysis of the acetal bonds, the resulting monosaccharides were derivatized with l-cysteine methyl ester and phenylisothiocyanate in pyridine [46]. By comparison with standards, a d absolute configuration was assigned for both glucose and galactose monosaccharides. 

Compound **2** was isolated as a yellowish amorphous solid. The molecular formula of **2** was determined by HRESIMS as C_41_H_70_O_13_. The spectroscopic data were very similar to those of **1**, thereby suggesting that both compounds were close analogues. Examination of the ^1^H NMR spectrum revealed the presence of an additional methyl group at δ_H_ 1.59 (d, *J* = 6.3 Hz, 3H, H_3_-24^2^) placed on the double bond at C-24^1^, therefore, leading to a poriferastane skeleton. The relative configuration of **2** was found to be the same as that of poecillastroside A based on nOe correlations. A key H_3_-24^2^/H_2_-23 nOe led us to assign the configuration of the double bond as *E*.

Compound **3** was isolated as a pale yellowish amorphous solid with the same molecular formula C_41_H_70_O_13_. Both compounds **2** and **3** are, therefore, isomers. The ^1^H NMR spectra were almost identical except for a deshielding of the signal corresponding to H-25, from δ_H_ 2.24 in **2** to δ_H_ 2.85 for **3**. We first supposed that a change in the configuration of the double had occurred. Due to the low amount of compound available, the corresponding carbons were not visible neither in the ^13^C NMR spectrum nor in the HSQC, HMBC spectra. We, therefore, decided to enhance the sensitivity of the HSQC spectrum using the recently developed Pure Shift HSQC experiment [47]. Gratifyingly, we were then able to observe both HSQC spots corresponding to C-24^1^ and C-25 (Figure S24). The shielding of the C-25 signal from δ_C_ 36.0 for **2** to δ_C_ 29.8 for **3** clearly confirmed a *Z* configuration for the double bond of 3.

Compound **4** was isolated as a pale yellowish amorphous solid with a molecular formula C_41_H_68_O_13_. The ^1^H NMR spectrum of **4** was very similar to the one of **2** except for the absence of the signals corresponding to the AB system of H_2_-18 and a shielding observed for δ_H_ 2.64 (m, 1H, H-12a). The only explanation consistent with all these observations, including the molecular formula, was the replacement of the hydroxyl group at C-18 by a carboxylic acid. This interpretation was further supported by a key H-17/C-18 HMBC correlation. Based on the chemical shift of the signal H-25 the configuration of the double bond was found to be the same as in **2**.

**Figure 2:**
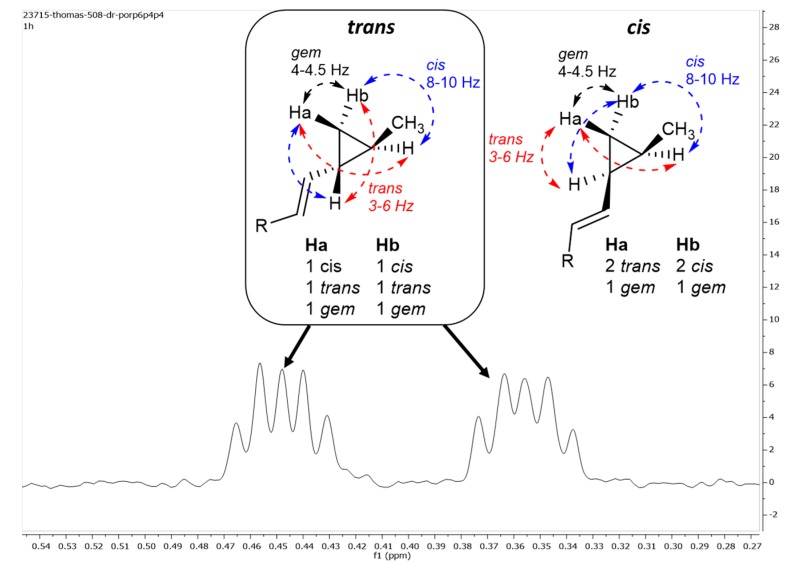
Assignment of the relative configuration of the disubstituted cyclopropane through ^1^H NMR coupling constants [52].

Compound **5** was isolated as a white amorphous solid with a molecular formula of C_43_H_66_O_15_. Despite strong differences when compared with **1**–**4**, the NMR data of **5** evidenced that the molecule was a steroidal saponin (Table 2). The aglycone exhibited an unusual skeleton with the presence of a terminal methylated cyclopropyl ring on the lateral chain. This assumption was based on the shielded signals of H-25 and H-26 but also by COSY, HSQC, and HMBC data analyses with the key H-27/C-24, H-27/C-26 HMBC correlations. Further analysis of ^1^H NMR data revealed the *E* geometry of the olefinic bond (*J*_H-22,-23_ = 15.2 Hz). No clear nOe correlations were observed for assessing the relative configuration around the cyclopropane ring. Gratifyingly, comparison with literature data and synthetic analogues of sterols with an identical side-chain led us to propose a *trans* configuration for the substituents at C-24 and C-25 of this ring [48,49,50,51]. To confirm this configuration in our case, we decided to look further into the coupling constants of the signals corresponding to the cyclopropane protons. Only the signals of the methylene and their multiplicity were clearly identified in the ^1^H NMR spectrum (Figure 2). In the case of a *trans* configuration of the two substituents around the cyclopropane, H_a_ and H_b_ would have the same splitting pattern as they would have in the presence of a pseudo C2 axial symmetry perpendicular to the cyclopropane plane. The ^3^*J* coupling constants between protons in a *cis* configuration are known to be between 8 and 10 Hz while values below 7 Hz are always observed when placed in a *trans* configuration. The multiplicity for both signals is observed as a doublet or triplet with coupling constants around 8 and 4 Hz, respectively. This same splitting pattern for both signals is only consistent for a *trans* configuration. Indeed, for a *cis* configuration, one of the two *gem* protons H_b_ would exhibit two large ^3^*J* coupling constants of 8 Hz. We, therefore, confirm a *trans* configuration for the two substituents and estimate the *gem* ^2^*J* coupling constants between H_a_ and H_b_ to be around 4 Hz. The presence of a carboxyl group at C-18 was inferred first from the HRESIMS data and then from the deshielding of H-12a, exactly in the same manner as for compound **4**. Another difference with **4** arose from the absence of the signal corresponding to the oxygenated methine at C-16. This feature was confirmed by COSY, HSQC, and HMBC correlations. Looking at the glycosidic part of the saponin, the relative configuration was similar to those of **1**–**4**, therefore, confirming one galactose linked to the aglycone and one glucose linked to the galactose. HMBC showed long-range correlations between H-1″/C-3′, H-2′/C_Ac_ (δ_C_ 172.2), and H-6″/C_Ac_ (δ_C_ 172.8), thereby indicating the presence of two acetyl groups at C-2′ and C-6″. Unlike compounds **1**–**4**, the glycosidic link between both sugar residues was placed at C-3′ of the galactose. Deshielding of the signal of C-3′ at δ_C_ 82.4 in the ^13^C NMR spectrum confirmed this new substitution pattern.

Compound **6** was isolated as a white amorphous solid with a molecular formula of C_41_H_66_O_13_. The spectroscopic data were very similar to those of **5**, thereby suggesting a close aglycone moiety. However, some changes were noticed by HSQC and HMBC analyses. Indeed, in the aglycone moiety, we observed the same AB system for H_2_-18 as that present in compounds **1**–**3**. The long-range H-17/C-18 HMBC correlation confirmed the presence of an oxygenated methylene at C-13. In the d-β-glucose residue, the chemical shifts, and the COSY data were consistent with a terminal primary alcohol at C-6″, thereby implying the loss of the acetate at this position.

Compound **7** was isolated as a white amorphous solid with a molecular formula C_43_H_68_O_14_. The ^1^H NMR spectrum evidenced the fact that **7** is a close analogue of **6**. The long-range H-6″/C_Ac_ (δ_C_ 172.8) HMBC correlation revealed the presence of an acetate group linked at O-6″ as in compound **5**. The relative configuration of **7** was the same as those of **5** and **6**.

Poecillastrosides A–G were tested in a panel of antimicrobial and cytotoxicity assays, including antibacterial activity against Gram positive (methicillin resistant (MRSA) and methicillin sensitive (MSSA) *Staphylococcus aureus*), and Gram negative bacteria (*Escherichia coli*, *Klebsiella pneumoniae*, *Pseudomonas aeruginosa*, and *Acinetobacter baumannii*), antifungal activity against *Aspergillus fumigatus*, and cytotoxicity against the hepatic tumoral cell line hep_G2. Poecillastrosides D (**4**) (MIC_90_ = 6 µg/mL) and E (**5**) (MIC_90_ = 24 µg/mL) were the only two molecules active in the assay against *A. fumigatus*, revealing a key role of the carboxylic acid functionality at C-18 in the antifungal activity of this structural class. On the other hand, cytotoxicity assays also revealed weak activity of some members of the family against the hep_G2 human cell line, with IC_50_ values of 38, 28, and 89 µg/mL for poecillastrosides B, C, and D (**2**–**4**), respectively. None of the compounds of this family displayed activity against the bacterial pathogens at the highest concentration tested (96 µg/mL for compound **1**–**5**, and 64 µg/mL for compounds **6** and **7**).

## 3. Material and Methods

### 3.1. General Experimental Procedures

Optical rotations were recorded with a PerkinElmer 343 polarimeter equipped with a 10 cm microcell and a sodium lamp. UV measurements were obtained by extraction of the Diode Array Detector (DAD) signal of the Ultra-High Pressure Liquid Chromatography (UHPLC) Dionex Ultimate 3000 (Thermo Scientific, Waltham, MA, USA). NMR experiments were performed on a 500 MHz (Advance, Bruker, Billerica, MA, USA) or a 600 MHz (Agilent, Santa Clara, CA, USA) spectrometer. Chemical shifts (*δ* in ppm) are referenced to the carbon (*δ*_C_ 49.0) and residual proton (*δ*_H_ 3.31) signals of CD_3_OD. High-resolution mass spectra (HRESIMS) were obtained from a mass spectrometer Agilent 6540. HPLC separation and purification were carried out on a Jasco LC-2000 series equipped with a UV detector coupled with an Evaporative Light Scattering Detector, ELSD (Sedere, Alfortville, France).

### 3.2. Biological Material

*Poecillastra compressa* (Bowerbank, 1866) was collected in the Mediterranean Sea, off the French coasts, on 15 October 2014 at 200 m depth using a Remotely Operated Vehicle (Super Achille, COMEX S.A., Marseille, France). The voucher specimen “CS2ACHP09_ECH04” is kept at the Marine Station of Endoume (OSU Institut Pythéas, Marseille, France).

### 3.3. Extraction and Isolation

The dry sponge sample (43.1 g) was ground with a mortar and extracted with a mixture of CH_3_OH/CH_2_Cl_2_ (1:1, *v*/*v*) at room temperature, yielding 7.9 g (18% yield from dry-weight) of extract after solvent evaporation. The crude extract was fractionated by RP-C18 vacuum liquid chromatography (elution with a decreasing polarity gradient of H_2_O/CH_3_OH from 1:0 to 0:1, then CH_3_OH/CH_2_Cl_2_ from 1:0 to 0:1). The CH_3_OH (422 mg) fraction was then subjected to RP-HPLC on a preparative phenylhexyl column, 250 mm × 19 mm, 5 µm (Xselect, Waters, Milford, CT, USA), using a mobile phase of water (A) and acetonitrile (B). The method was developed on 30 min acquisition time: isocratic 60% B for 15 min, then linear gradient to 98% B in 1 min, held at 98% B for 10 min, back to 60% B in 1 min, and held at that percentage of B for 3 min. Selected fractions from this chromatography were then purified by RP-HPLC on a semi-preparative HTec C18 column, 250 mm × 10 mm, 5 µm (Nucleodur, Macherey-Nagel, Düren, Germany), with the following methods for each subsequent purification: isocratic 47% B to afford pure **1** (4.3 mg, 9.98 × 10^−3^% *w*/*w*), isocratic 49% B to afford **2** (6.2 mg, 1.44 × 10^−2^% *w*/*w*) and **3** (1.4 mg, 3.49 × 10^−3^% *w*/*w*), isocratic 50% B to afford **4** (1.6 mg, 3.71 × 10^−3^% *w*/*w*), isocratic 51% B to afford **5** (0.9 mg, 2.09 × 10^−3^% *w*/*w*), and isocratic 53% B to afford **6** (0.7 mg, 1.62 × 10^−3^% *w*/*w*) and **7** (0.8 mg, 1.86 × 10^−3^% *w*/*w*).

Poecillastroside A (**1**): Yellow, amorphous solid; ${\left[\alpha \right]}_{D}^{20}$ +12.8 (*c* 0.1, CH_3_OH); UV (DAD) λ_max_ 195 nm; ^1^H NMR and ^13^C NMR data, see Table 1; HRESIMS (−) *m*/*z* 755.4582 [M − H]^−^ (calcd. for C_40_H_67_O_13_, 755.4587, ∆ − 0.7 ppm). 

Poecillastroside B (**2**): Yellow, amorphous solid; ${\left[\alpha \right]}_{D}^{20}$ +13.2 (*c* 0.1, CH_3_OH); UV (DAD) λ_max_ 210 nm; ^1^H NMR and ^13^C NMR data, see Table 1; HRESIMS (−) *m*/*z* 769.4743 [M − H]^−^ (calcd. for C_41_H_69_O_13_, 769.4744, ∆ − 0.1 ppm). 

Poecillastroside C (**3**): Yellow, amorphous solid; ${\left[\alpha \right]}_{D}^{20}$ +13.0 (*c* 0.1, CH_3_OH); UV (DAD) λ_max_ 212 nm; ^1^H NMR and ^13^C NMR data, see Table 1; HRESIMS (−) *m*/*z* 769.4745 [M − H]^−^ (calcd. for C_41_H_69_O_13_, 769.4744, ∆ + 0.1 ppm). 

Poecillastroside D (**4**): Yellow, amorphous solid; ${\left[\alpha \right]}_{D}^{20}$ +8.9 (*c* 0.1, CH_3_OH); UV (DAD) λ_max_ 222 nm; ^1^H NMR and ^13^C NMR data, see Table 1; HRESIMS (+) *m*/*z* 791.4567 [M + Na]^+^ (calcd. for C_41_H_68_NaO_13_, 791.4563, ∆ + 0.5 ppm). 

Poecillastroside E (**5**): White, amorphous solid; ${\left[\alpha \right]}_{D}^{20}$ −6.2 (*c* 0.1, CH_3_OH); UV (DAD) λ_max_ 220 nm; ^1^H NMR and ^13^C NMR data, see Table 2; HRESIMS (+) *m*/*z* 845.4307 [M + Na]^+^ (calcd. for C_43_H_66_NaO_15_, 845.4299, ∆ + 0.9 ppm). 

Poecillastroside F (**6**): White, amorphous solid; ${\left[\alpha \right]}_{D}^{20}$ −27.3 (*c* 0.1, CH_3_OH); UV (DAD) λ_max_ 222 nm; ^1^H NMR and ^13^C NMR data, see Table 2; HRESIMS (+) *m*/*z* 789.4405 [M + Na]^+^ (calcd. for C_41_H_66_NaO_13_, 789.4401, ∆ + 0.5 ppm). 

Poecillastroside G (**7**): White, amorphous solid; ${\left[\alpha \right]}_{D}^{20}$ −14.1 (*c* 0.1, CH_3_OH); UV (DAD) λ_max_ 225 nm; ^1^H NMR and ^13^C NMR NMR data, see Table 2; HRESIMS (+) *m*/*z* 831.4518 [M + Na]^+^ (calcd. for C_43_H6_8_NaO_14_, 831.4507, ∆ + 1.3 ppm). 

### 3.4. Determination of the Absolute Configuration of the Pyranoses

Hydrolysis of glycosides and derivatization of the subsequent monosaccharides were performed individually following previously described methodologies [46]. The monosaccharide derivatives separation was carried out by UHPLC-HRMS on Acquity BEH (Ethylene Bridged Hybrid) C18 1.7 µm, 2.1 mm × 100 mm (Waters). The column was heated at 40 °C. The eluent consisted of water with 0.1% formic acid (A) and acetonitrile/methanol/isopropanol (50:25:25, *v*/*v*/*v*) with 0.1% formic acid (B). The analysis was performed in isocratic mode at 13% B and at a flow rate of 360 µL/min. The injection volume was set at 3 µL. The identity of all monosaccharide derivatives was confirmed after extraction of the ion [M + H]^+^ at *m*/*z* 433.1098 (Figure S55).

### 3.5. Evaluation of the Biological Activities

Compounds **1**–**7** were tested for their ability to inhibit the growth of Gram positive bacteria (*S. aureus* ATCC29213 (MSSA), and *S. aureus* MB5393 (MRSA)) and Gram negative bacteria (*E. coli* ATCC25922, *K. pneumoniae* ATCC700603, *P. aeruginosa* PAO1, and *A. baumannii* CL5973), and fungi (*A. fumigatus* ATCC46645), following previously described methodologies [53,54]. Cytotoxic activity against the hepatic human tumoral cell line hep_G2 was determined as previously reported [55].

## 4. Conclusions

Poecillastrosides A–G (**1**–**7**) share an unusual oxidized methyl at C-18, and they are the first saponins exhibiting this feature. The structures of poecillastrosides E–G (**5**–**7**) also incorporate a terminal methylated cyclopropyl ring already known in some sponge steroids and already investigated for biosynthetic studies [56]. This cyclopropanation process could lead to the cholestane skeleton, then ergostane, and finally poriferastane, all of them being present in the metabolome of this sponge. Many sterols containing a cyclopropyl ring have been isolated to date [57], but to our best knowledge, this is the first time that saponins containing a 3-membered ring on the side-chain have been reported. Poecillastrosides D (**4**) and E (**5**), bearing a carboxylic acid at C-18, were found to be the most bioactive compounds in the antimicrobial bioassays with an interesting antifungal activity against *Aspergillus fumigatus*.

## Figures and Tables

**Table 1:** NMR spectroscopic data for poecillastrosides A–D (**1**–**4**) in CD_3_OD (500 MHz for ^1^H NMR data and 125 MHz for ^13^C NMR data).

No.	1		2		3		4	
	δ_H_, mult. (*J* in Hz)	δ_C_	δ_H_, mult. (*J* in Hz)	δ_C_	δ_H_, mult. (*J* in Hz)	δ_C_	δ_H_, mult. (*J* in Hz)	δ_C_
**1**	1.70, m	38.1	1.69, m	38.1	1.69, m	38.1	1.69, m	38.2
	0.98, m		0.98, m		0.98, m		0.98, m	
**2**	1.90, m	30.5	1.90, m	30.5	1.90, m	30.5	1.92, m	30.5
	1.50, m		1.50, m		1.50, m		1.48, m	
**3**	3.72, m	80.2	3.72, m	80.2	3.72, m	80.2	3.72, m	80.3
**4**	1.71, m	35.5	1.71, m	35.6	1.71, m	35.5	1.70, m	35.6
	1.34, m		1.34, m		1.34, m		1.32, m	
**5**	1.12, m	46.2	1.12, m	46.2	1.12, m	46.2	1.12, m	46.1
**6**	1.34, m	29.9	1.34, m	29.9	1.34, m	29.8	1.32, m	29.9
	1.32, m		1.31, m		1.31, m		1.29, m	
**7**	1.73, m	33.3	1.74, m	33.3	1.75, m	33.3	1.74, m	33.1
	0.94, m		0.94, m		0.95, m		0.92, m	
**8**	1.67, m	36.1	1.67, m	36.1	1.67, m	36.1	1.38, m	38.5
**9**	0.75, m	56.2	0.74, m	56.2	0.74, m	56.2	0.72, m	55.9
**10**		36.8		36.9		36.8		36.8
**11**	1.51, m	22.8	1.52, m	22.8	1.52, m	22.8	1.63, m	24.4
	1.31, m		1.32, m		1.32, m		1.34, m	
**12**	2.01, m	38.9	2.01, m	38.8	2.01, m	38.8	2.64, m	38.2
	1.11, m		1.10, m		1.10, m		1.09, m	
**13**		48.1		48.1		48.1		55.8
**14**	1.10, m	55.1	1.10, m	55.1	1.10, m	55.1	1.39, m	58.4
**15**	2.17, m	38.5	2.16, m	38.6	2.16, m	38.6	1.81, m	26.5
	1.34, m		1.33, m		1.33, m		1.19, m	
**16**	4.26, td (7.7, 3.7)	72.8	4.26, td (7.9, 3.7)	72.8	4.26, td (7.9, 3.7)	72.8	1.80, m	24.4
							0.89, m	
**17**	1.19, m	62.3	1.19, m	62.3	1.19, m	62.3	1.48, m	57.4
**18**	3.95, d (11.6)	62.6	3.95, d (11.6)	62.6	3.95, d (11.6)	62.4		180.1
	3.59, d (11.6)		3.60, d (11.6)		3.60, d (11.6)			
**19**	0.88, s	12.8	0.88, s	12.8	0.88, s	12.9	0.76, s	12.8
**20**	1.94, m	31.6	1.93, m	32.2	1.93, m	32.0	1.49, m	38.8
**21**	1.02, d (6.8)	19.0	1.07, d (6.7)	19.1	1.02, d (6.7)	19.1	1.09, d (6.3)	19.1
**22**	1.87, m	35.5	1.73, m	35.5	1.83, m	36.8	1.45, m	36.0
	1.21, m		1.18, m		1.18, m		1.14, m	
**23**	2.15, m	32.4	2.13, m	26.8	2.04, m	29.1	2.07, m	29.9
	1.98, m		1.94, m		1.83, m		1.90, m	
**24**		158.0		148.2		146.9		147.9
**24^1^**	4.71, br s 4.70, br s	106.7	5.19, q (6.7)	116.6	5.17, q (6.7)	117.7	5.18, q (6.7)	116.8
**24^2^**			1.59, d (6.3)	13.4	1.58, d (6.3)	12.8	1.56, d (6.7)	13.4
**25**	2.29, h (6.5)	34.8	2.24, m	36.0	2.85, m	29.8	2.19, m	35.6
**26**	1.03, d (6.8)	22.5	0.99, d (6.8)	22.7	0.99, d (6.8)	21.4	0.98, d (6.8)	22.7
**27**	1.03, d (6.8)	22.3	0.99, d (6.8)	22.6	0.99, d (6.8)	21.4	0.98, d (6.8)	22.6
**1′**	4.49, d (7.6)	101.8	4.49, d (7.6)	101.8	4.49, d (7.6)	101.8	4.48, d (7.5)	101.8
**2′**	3.70, m	80.8	3.69, t (10.2)	80.8	3.69, t (10.2)	80.8	3.70, t (10.2)	80.8
**3′**	3.65, dd (9.6, 3.3)	74.8	3.65, dd (9.6, 3.3)	74.8	3.65, dd (9.6, 3.3)	74.8	3.64, dd (9.5, 3.3)	74.8
**4′**	3.84, d (3.2)	70.0	3.84, d (3.2)	70.0	3.84, d (3.2)	70.0	3.84, d (3.1)	70.0
**5′**	3.50, t (6.1)	76.4	3.50, t (6.1)	76.4	3.50, t (6.1)	76.4	3.49, t (6.2)	76.4
**6′**	3.73, m	62.7	3.73, m	62.7	3.73, m	62.7	3.73, m	62.7
	3.71, m		3.71, m		3.71, m		3.71, m	
**1″**	4.56, d (7.9)	105.2	4.56, d (7.9)	105.2	4.56, d (7.9)	105.2	4.56, d (7.9)	105.2
**2″**	3.25, dd (9.1, 7.9)	75.8	3.25, dd (9.1, 7.9)	75.8	3.25, dd (9.1, 7.9)	75.8	3.25, dd (9.0, 7.8)	75.8
**3″**	3.37, t (8.8)	77.7	3.37, t (8.8)	77.7	3.37, t (8.8)	77.7	3.37, t (8.9)	77.7
**4″**	3.33, t (9.3)	71.4	3.33, t (9.3)	71.4	3.33, t (9.3)	71.4	3.33, t (9.4)	71.4
**5″**	3.29, m	78.4	3.29, m	78.4	3.29, m	78.4	3.28, m	78.4
**6″**	3.84, dd (11.2, 2.3)	62.4	3.84, dd (11.1, 2.3)	62.4	3.84, dd (11.1, 2.3)	62.4	3.84, dd (13.5, 2.8)	62.4
	3.71, m		3.71, m		3.71, m		3.71, m	

**Table 2:** NMR spectroscopic data for poecillastrosides E–G (**5**–**7**) in CD_3_OD (500 MHz for ^1^H NMR data and 125 MHz for ^13^C NMR data of **5**; 600 MHz for ^1^H data and 150 MHz for ^13^C data of **6** and **7**).

No.	5		6		7	
	δ_H_, mult. (*J* in Hz)	δ_C_	δ_H_, mult. (*J* in Hz)	δ_C_	δ_H_, mult. (*J* in Hz)	δ_C_
**1**	1.70, m	38.0	1.72, m	38.2	1.72, m	38.2
	0.97, m		0.97, m		0.98, m	
**2**	1.85, m	30.4	1.86, m	30.7	1.87, m	30.8
	1.44, m		1.46, m		1.46, m	
**3**	3.62, m	79.9	3.63, m	80.0	3.62, m	80.0
**4**	1.58, m	35.8	1.58, m	35.9	1.58, m	36.0
	1.17, m		1.17, m		1.19, m	
**5**	1.12, m	46.0	1.09, m	46.1	1.10, m	46.1
**6**	1.32, m	30.3	1.32, m	29.9	1.31, m	30.4
	1.29, m		1.29, m		1.27, m	
**7**	1.76, m	33.1	1.68, m	33.5	1.67, m	33.5
	0.94, m		0.87, m		0.87, m	
**8**	1.53, m	38.8	1.43, m	37.1	1.43, m	37.0
**9**	0.73, m	55.9	0.68, m	56.0	0.68, m	56.0
**10**		36.7		36.8		36.8
**11**	1.63, m	24.4	1.53, m	22.3	1.53, m	22.3
	1.31, m		1.36, m		1.34, m	
**12**	2.63, m	38.4	2.44, d (12.8)	35.9	2.44, dt (12.7, 3.4)	35.9
	1.10, m		0.94, m		0.94, m	
**13**		55.6		47.9		47.9
**14**	1.38, m	58.4	1.11, m	57.6	1.12, m	57.6
**15**	1.75, m	30.8	1.70, m	29.9	1.71, m	29.9
	1.30, m		1.30, m		1.29, m	
**16**	1.78, m	25.8	1.54, m	25.0	1.54, m	24.9
	1.53, m		0.98, m		0.98, m	
**17**	1.46, m	57.3	1.15, m	58.2	1.16, m	58.1
**18**		180.1	3.65, d (11.5)	60.2	3.65, d (11.1)	60.4
			3.45, d (11.6)		3.45, d (11.7)	
**19**	0.73, s	12.7	0.83, s	12.7	0.83, s	12.7
**20**	1.92, m	42.4	2.26, m	41.7	2.26, m	41.7
**21**	1.07, d (6.3)	21.2	1.07, d (5.9)	22.1	1.07, d (6.4)	22.1
**22**	5.21, dd (15.1, 8.5)	134.6	5.22, dd (14.8, 9.0)	136.0	5.22, dd (15.2, 8.9)	136.0
**23**	4.90, m	132.4	4.94, dd (14.8, 8.1)	131.6	4.94, dd (15.2, 8.3)	131.6
**24**	0.96, m	23.4	0.93, m	23.4	0.93, m	23.4
**25**	0.62, m	15.5	0.62, m	15.5	0.62, m	15.5
**26**	0.44, td (9.0, 4.5)	15.2	0.45, m	15.2	0.45, m	15.2
	0.36, dt (9.0, 4.5)		0.36, m		0.35, m	
**27**	1.03, d (5.9)	18.8	1.03, d (5.8)	18.9	1.03, d (5.9)	18.9
**1′**	4.56, d (8.0)	101.1	4.55, d (7.9)	101.2	4.56, d (8.0)	101.2
**2′**	5.11, dd (8.4, 8.1)	72.5	5.12, dd (9.0, 7.7)	72.6	5.11, dd (10.1, 8.0)	72.4
**2′-Ac**	2.06, s	21.2	2.06, s	21.2	2.06, s	21.2
		172.2		171.2		172.2
**3′**	3.76, dd (10.2, 3.3)	82.4	3.80, dd (10.0, 2.8)	82.2	3.76, dd (10.1, 3.2)	82.4
**4′**	4.07, d (3.2)	70.2	4.11, d (3.1)	70.2	4.07, d (3.4)	70.2
**5′**	3.55, t (6.1)	76.4	3.56, t (6.2)	76.4	3.55, t (6.4)	76.4
**6′**	3.74, m	62.3	3.74, m	62.2	3.74, m	62.1
	3.73, m		3.72, m		3.72, m	
**1″**	4.39, d (7.6)	106.0	4.38, d (7.9)	106.0	4.38, d (8.0)	106.0
**2″**	3.21, t (8.3)	74.6	3.19, t (8.3)	74.8	3.21, t (8.3)	74.7
**3″**	3.32, t (10.1)	77.7	3.35, m	77.9	3.33, m	77.9
**4″**	3.28, t (9.6)	71.6	3.28, m	71.3	3.29, m	71.5
**5″**	3.46, m	75.3	3.64, m	80.0	3.46, m	75.3
**6″**	4.38, d (11.9)	64.7	3.84, m	62.5	4.38, dd (11.9, 2.7)	64.7
	4.20, dd (11.9, 6.1)		3.67, m		4.20, dd (11.9, 6.2)	
**6″-Ac**	2.06, s	20.8			2.06, s	20.8
		172.8				172.8

## Supplementary Materials

The following are available online at www.mdpi.com/1660-3397/15/7/199/s1: HRMS and ^1^H, ^13^C, COSY, TOCSY, HSQC, HMBC, and NOESY NMR data for compounds **1**–**7** as well as procedures for absolute configuration of compound **3**.
