# Supplementary material for: Poecillastrosides, Steroidal Saponins from the Mediterranean Deep-Sea Sponge Poecillastra compressa (Bowerbank, 1866)
Source: Mar Drugs. 2017 Jun 26;15(7):199. doi: 10.3390/md15070199 (PMC5532641; doi:10.3390/md15070199)
Supplement: Supplementary file 1 [file marinedrugs-15-00199-s001.pdf]

Supplementary Information for

Poecillastrosides, Steroidal Saponins from the Mediterranean Deep-Sea Sponge *Poecillastra compressa* (Bowerbank, 1866)

Kevin Calabro [1,2], Elaheh Lotfi Kalahroodi [3], Daniel O. Rodrigues [3,4], Caridad Díaz [5], Mercedes de la Cruz [5], Bastien Cautain [5], Rémi Laville [2], Fernando Reyes [5], Thierry Pérez [4], Bassam Soussi [3,6,7] and Olivier P. Thomas [1,3,*]

[1] National University of Ireland Galway, School of Chemistry, Marine Biodiscovery, University Road, Galway, Ireland; kevin.calabro@unice.fr (K.C.); olivier.thomas@nuigalway.ie (O.T.)
[2] Cosmo International Ingredients, 855 avenue du Docteur Maurice Donat, 06250 Mougins., France; remi.laville@airlquide.com (R.L.)
[3] Université Côte d'Azur, CNRS, OCA, IRD, Géoazur, 250 rue Albert Einstein, 06560 Valbonne, France; elaheh.lotfi-kalahroodi@univ-rennes1.fr (E.L.K.); daniel4rodrigues@gmail.com (D.R.)
[4] Institut Méditerranéen de Biodiversité et d'Ecologie marine et continentale. CNRS – Aix-Marseille Univ – IRD – Univ Avignon. Station Marine d'Endoume, rue de la batterie des lions, 13007, Marseille, France; thierry.perez@imbe.fr (T.P.)
[5] Fundación MEDINA, Centro de Excelencia en Investigación de Medicamentos Innovadores en Andalucía, Avda. del Conocimiento 34, Parque Tecnológico de Ciencias de la Salud, E-18016, Armilla, Granada, Spain; caridad.diaz@medinaandalucia.es (C.D.); mercedes.delacruz@medinaandalucia.es (M.C.); bastien.cautain@medinaandalucia.es (B.C.); fernando.reyes@medinaandalucia.es (F.R.)
[6] Department of Marine Sciences, University of Gothenburg, PO Box 460, SE40530 Gothenburg, Sweden; bassam.soussi@gu.se (B.S.)
[7] Oman Centre for Marine Biotechnology, PO Box 236, PC 103, Muscat, Oman

P4 **Figure S1.** (–)-HRESIMS analysis of **1**
P5 **Figure S2.** ^{1}H NMR spectrum of **1** at 500 MHz in CD_3OD
P6 **Figure S3.** COSY NMR spectrum of **1** at 500 MHz in CD_3OD
P7 **Figure S4.** TOCSY NMR spectrum of **1** at 500 MHz in CD_3OD
P8 **Figure S5.** NOESY NMR spectrum of **1** at 500 MHz in CD_3OD
P9 **Figure S6.** ^{13}C NMR spectrum of **1** at 125 MHz in CD_3OD
P10 **Figure S7.** HSQC NMR spectrum of **1** at 500 MHz in CD_3OD
P11 **Figure S8.** HMBC NMR spectrum of **1** at 500 MHz in CD_3OD
P12 **Figure S9.** (–)-HRESIMS analysis of **2**
P13 **Figure S10.** ^{1}H NMR spectrum of **2** at 500 MHz in CD_3OD
P14 **Figure S11.** COSY NMR spectrum of **2** at 500 MHz in CD_3OD
P15 **Figure S12.** TOCSY NMR spectrum of **2** at 500 MHz in CD_3OD
P16 **Figure S13.** NOESY NMR spectrum of **2** at 500 MHz in CD_3OD
P17 **Figure S14.** ^{13}C NMR spectrum of **2** at 125 MHz in CD_3OD
P18 **Figure S15.** HSQC NMR spectrum of **2** at 500 MHz in CD_3OD
P19 **Figure S16.** HMBC NMR spectrum of **2** at 500 MHz in CD_3OD
P20 **Figure S17.** (–)-HRESIMS analysis of **3**
P21 **Figure S18.** ^{1}H NMR spectrum of **3** at 500 MHz in CD_3OD
P22 **Figure S19.** COSY NMR spectrum of **3** at 500 MHz in CD_3OD
P23 **Figure S20.** TOCSY NMR spectrum of **3** at 500 MHz in CD_3OD
P24 **Figure S21.** ^{13}C NMR spectrum of **3** at 125 MHz in CD_3OD
P25 **Figure S22.** HSQC NMR spectrum of **3** at 500 MHz in CD_3OD
P26 **Figure S23.** PSYCHE_1D NMR spectrum of **3** at 500 MHz in CD_3OD
P27 **Figure S24.** PS-HSQC NMR spectrum of **3** at 500 MHz in CD_3OD
P28 **Figure S25.** HMBC NMR spectrum of **3** at 500 MHz in CD_3OD
P29 **Figure S26.** (+)-HRESIMS analysis of **4**
P30 **Figure S27.** ^{1}H NMR spectrum of **4** at 500 MHz in CD_3OD
P31 **Figure S28.** COSY NMR spectrum of **4** at 500 MHz in CD_3OD
P32 **Figure S29.** TOCSY NMR spectrum of **4** at 500 MHz in CD_3OD
P33 **Figure S30.** ^{13}C NMR spectrum of **4** at 125 MHz in CD_3OD
P34 **Figure S31.** HSQC NMR spectrum of **4** at 500 MHz in CD_3OD
P35 **Figure S32.** HMBC NMR spectrum of **4** at 500 MHz in CD_3OD
P36 **Figure S33.** (+)-HRESIMS analysis of **5**
P37 **Figure S34.** ^{1}H NMR spectrum of **5** at 500 MHz in CD_3OD
P38 **Figure S35.** COSY NMR spectrum of **5** at 500 MHz in CD_3OD
P39 **Figure S36.** NOESY NMR spectrum of **5** at 500 MHz in CD_3OD
P40 **Figure S37.** ^{13}C NMR spectrum of **5** at 125 MHz in CD_3OD
P41 **Figure S38.** HSQC NMR spectrum of **5** at 500 MHz in CD_3OD
P42 **Figure S39.** HMBC NMR spectrum of **5** at 500 MHz in CD_3OD
P43 **Figure S40.** (+)-HRESIMS analysis of **6**
P44 **Figure S41.** ^{1}H NMR spectrum of **6** at 600 MHz in CD_3OD
P45 **Figure S42.** COSY NMR spectrum of **6** at 600 MHz in CD_3OD
P46 **Figure S43.** ^{13}C NMR spectrum of **6** at 150 MHz in CD_3OD
P47 **Figure S44.** HSQC NMR spectrum of **6** at 600 MHz in CD_3OD
P48 **Figure S45.** HMBC NMR spectrum of **6** at 600 MHz in CD_3OD

P49 **Figure S46.** (+)-HRESIMS analysis of **7**
P50 **Figure S47.** ^{1}H NMR spectrum of **7** at 600 MHz in CD_3OD
P51 **Figure S48.** COSY NMR spectrum of **7** at 600 MHz in CD_3OD
P52 **Figure S49.** TOCSY NMR spectrum of **7** at 600 MHz in CD_3OD
P53 **Figure S50.** ^{13}C NMR spectrum of **7** at 150 MHz in CD_3OD
P54 **Figure S51.** HSQC NMR spectrum of **7** at 600 MHz in CD_3OD
P55 **Figure S52.** HMBC NMR spectrum of **7** at 600 MHz in CD_3OD
P56 **Figure S53.** UPLC-qToF analysis of the four monosaccharide derivatives;
Figure S54. Absolute configuration of pyranose moieties of **3;**
Figure S55. ESI-(+) spectrum of D-(+)-glucose derivative

Compound Formula	Name	RT	Algorithm
$C_{40}H_{68}O_{13}$	**Poecillastroside A**	6.251	Spectrum Extraction

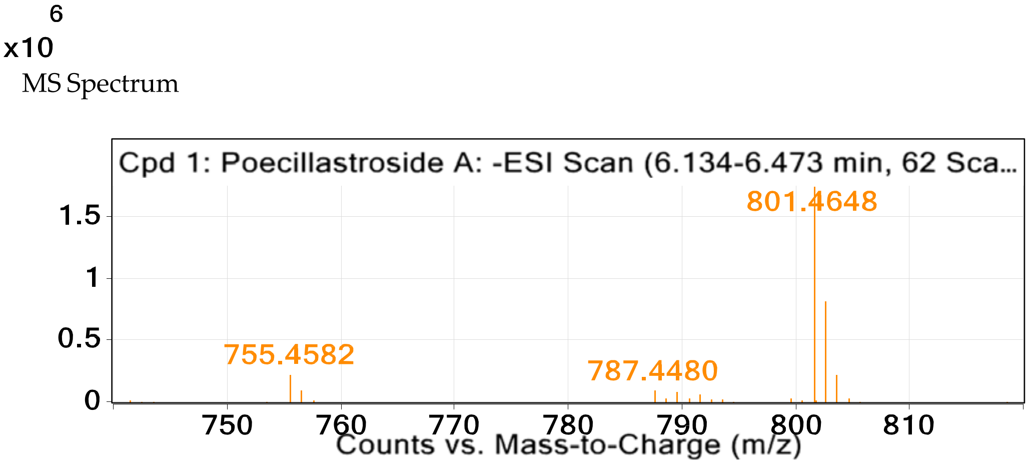

Figure S1. (–)-HRESIMS analysis of **1**.

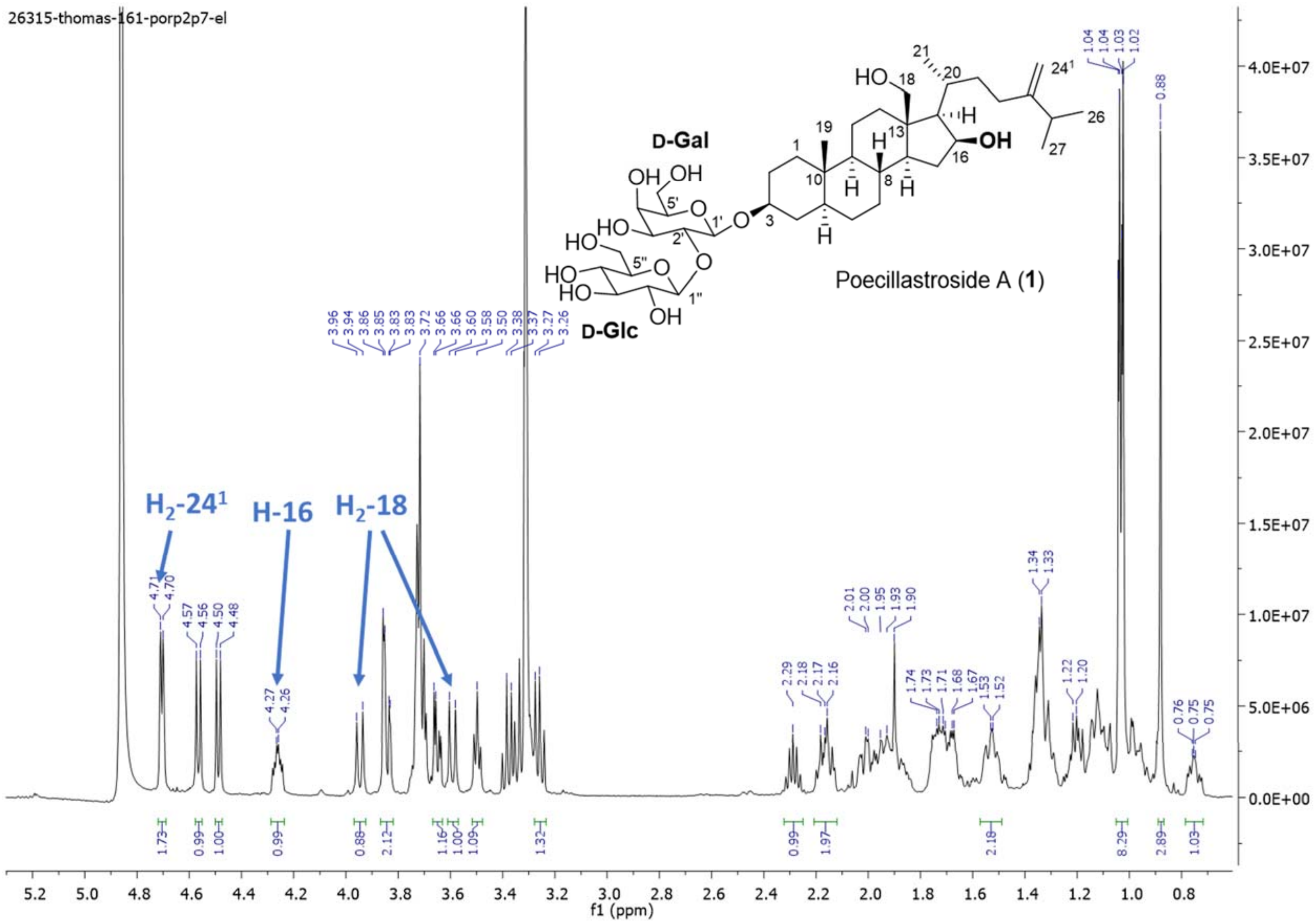

Figure S2. ^{1}H NMR spectrum of **1** at 500 MHz in CD_3OD

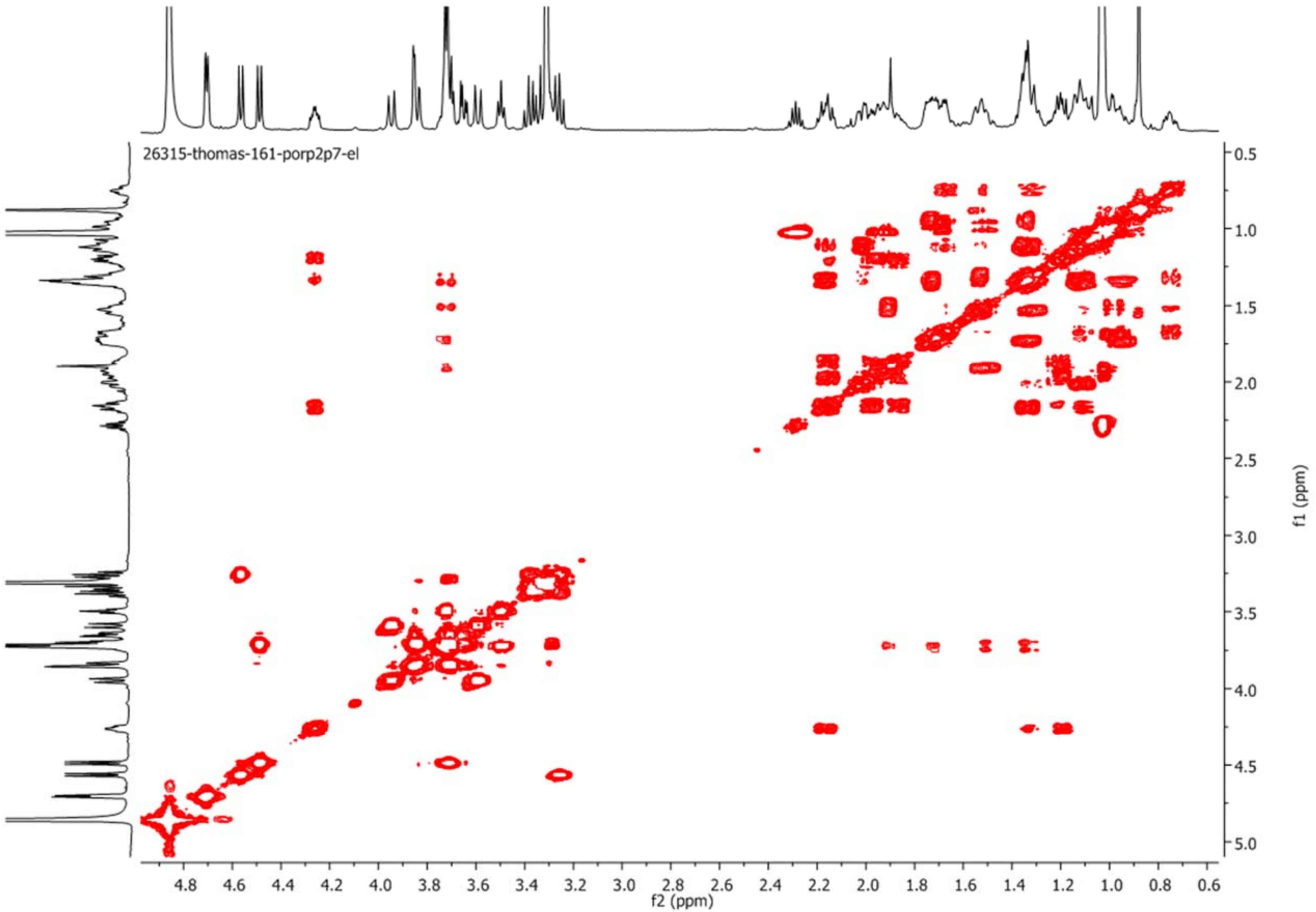

Figure S3. COSY NMR spectrum of **1** at 500 MHz in CD_3OD

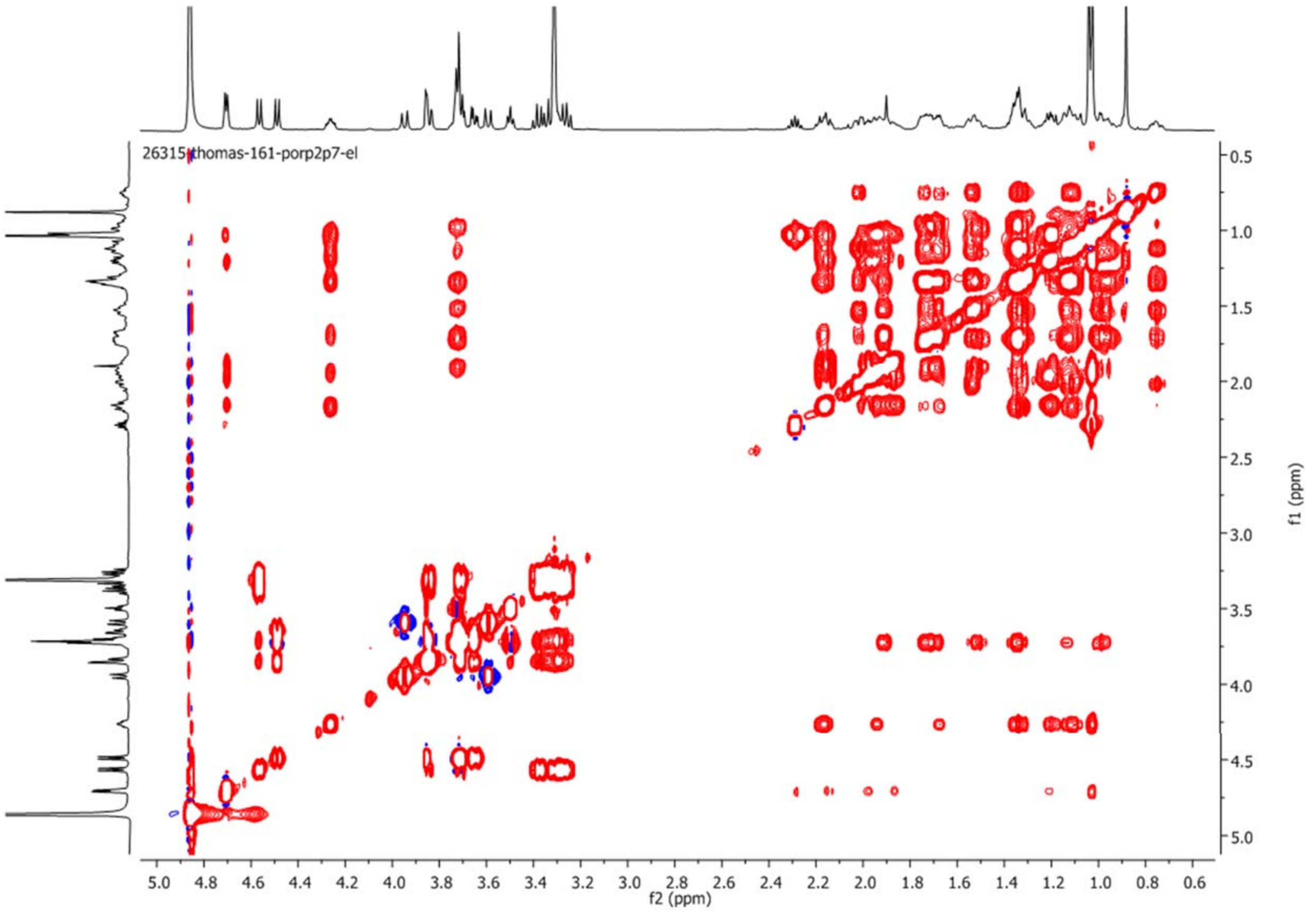

Figure S4. TOCSY NMR spectrum of **1** at 500 MHz in CD_3OD

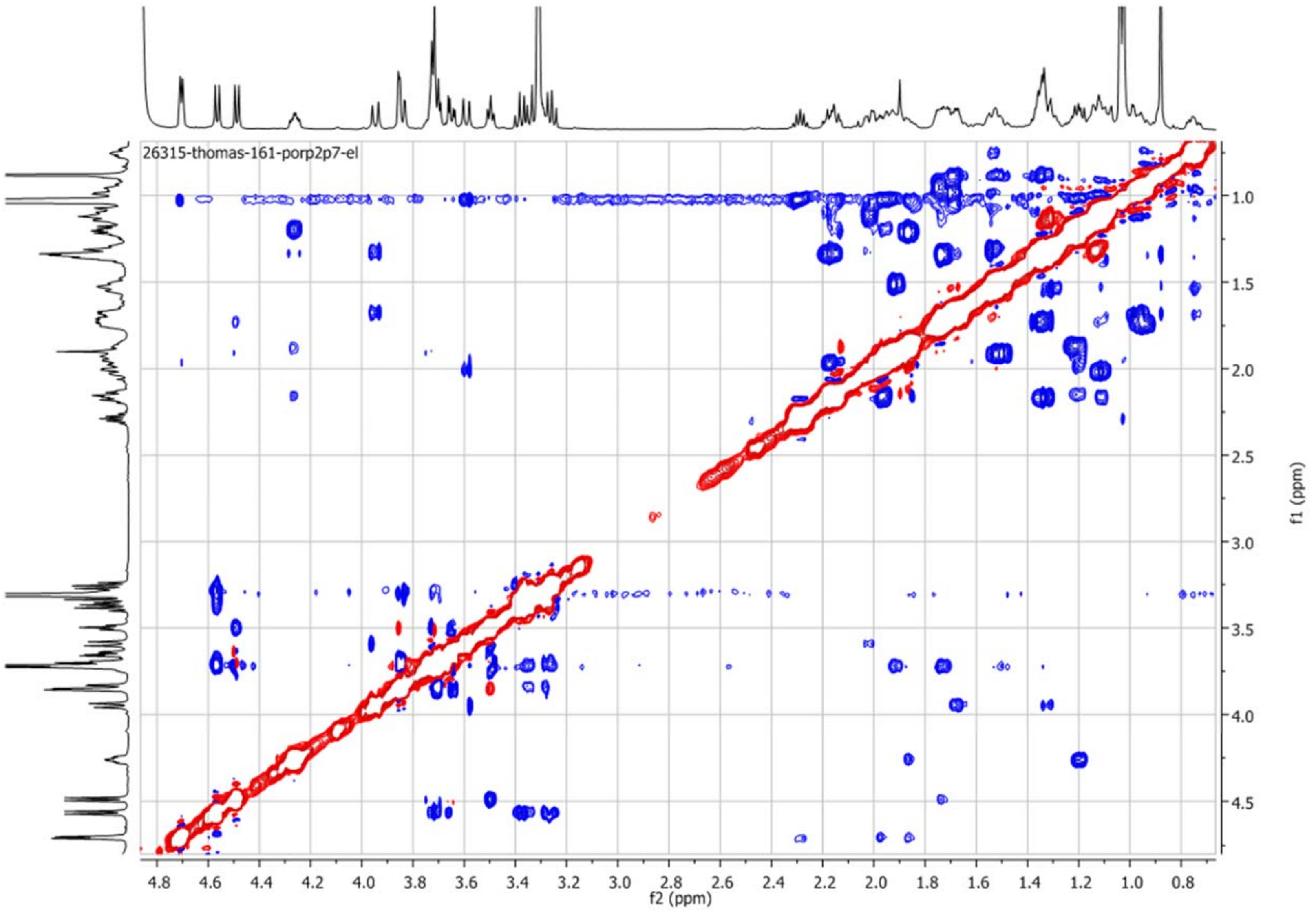

Figure S5. NOESY NMR spectrum of **1** at 500 MHz in CD_3OD

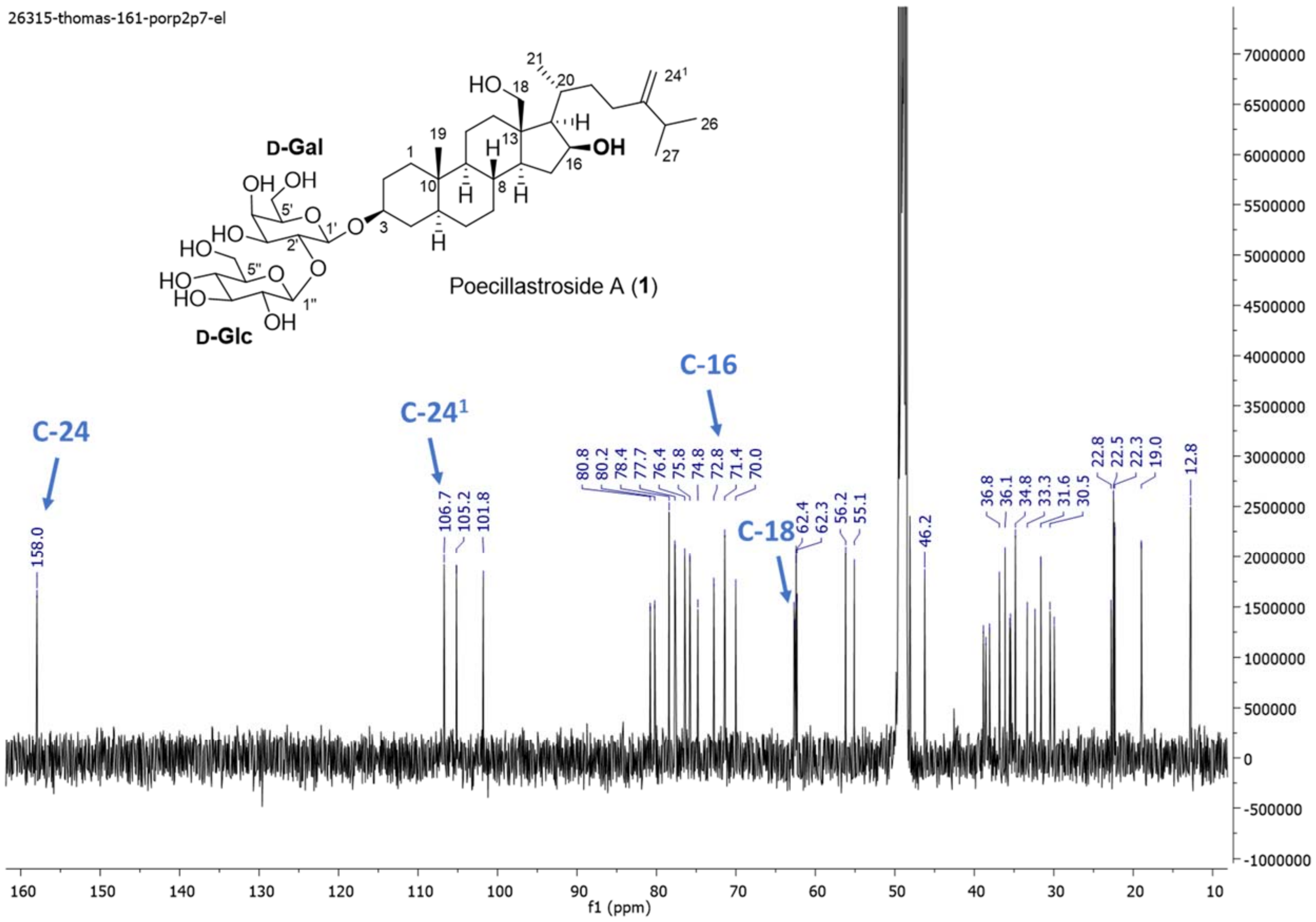

Figure S6. ^{13}C NMR spectrum of **1** at 125 MHz in CD_3OD

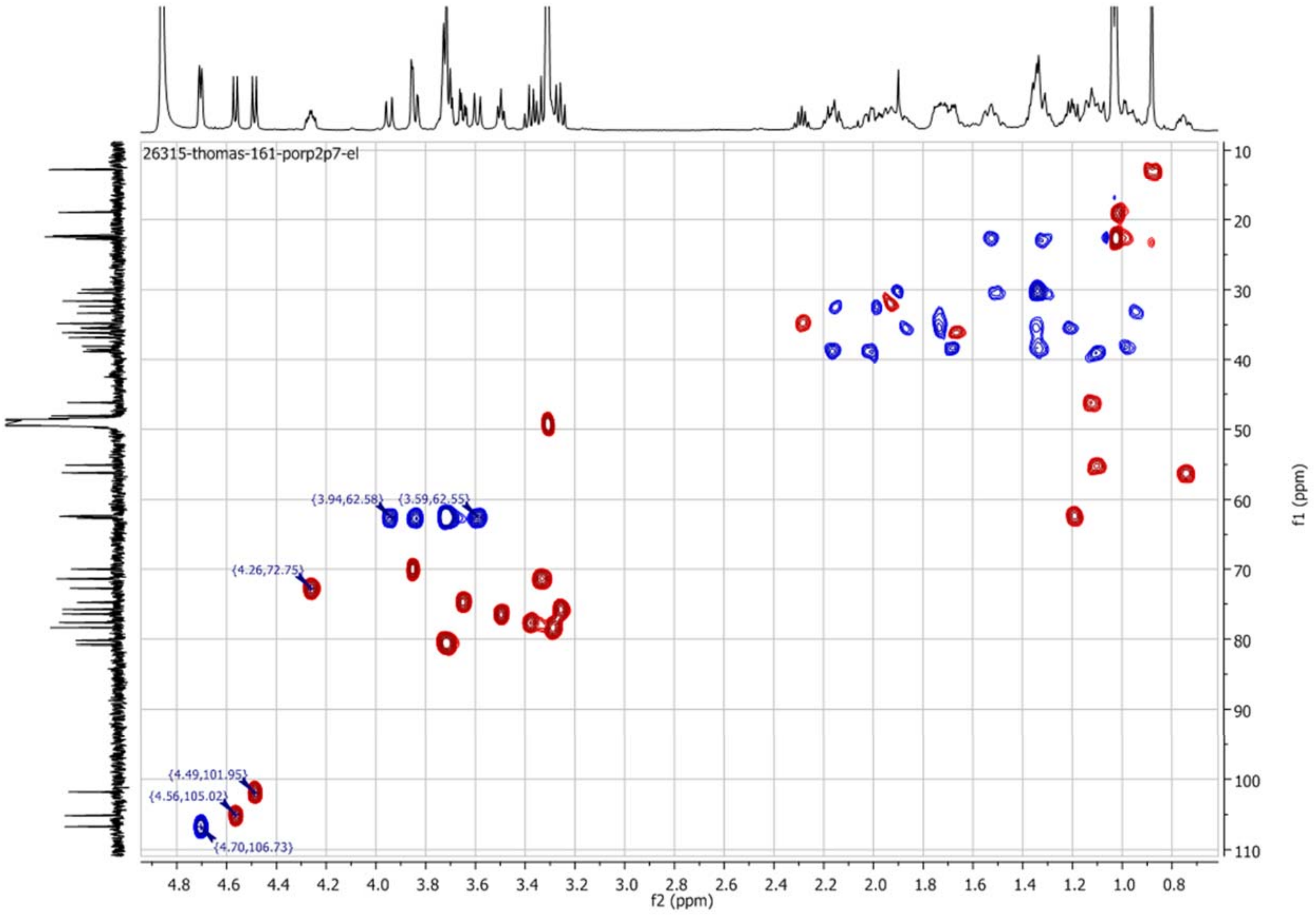

Figure S7. HSQC NMR spectrum of **1** at 500 MHz in CD_3OD

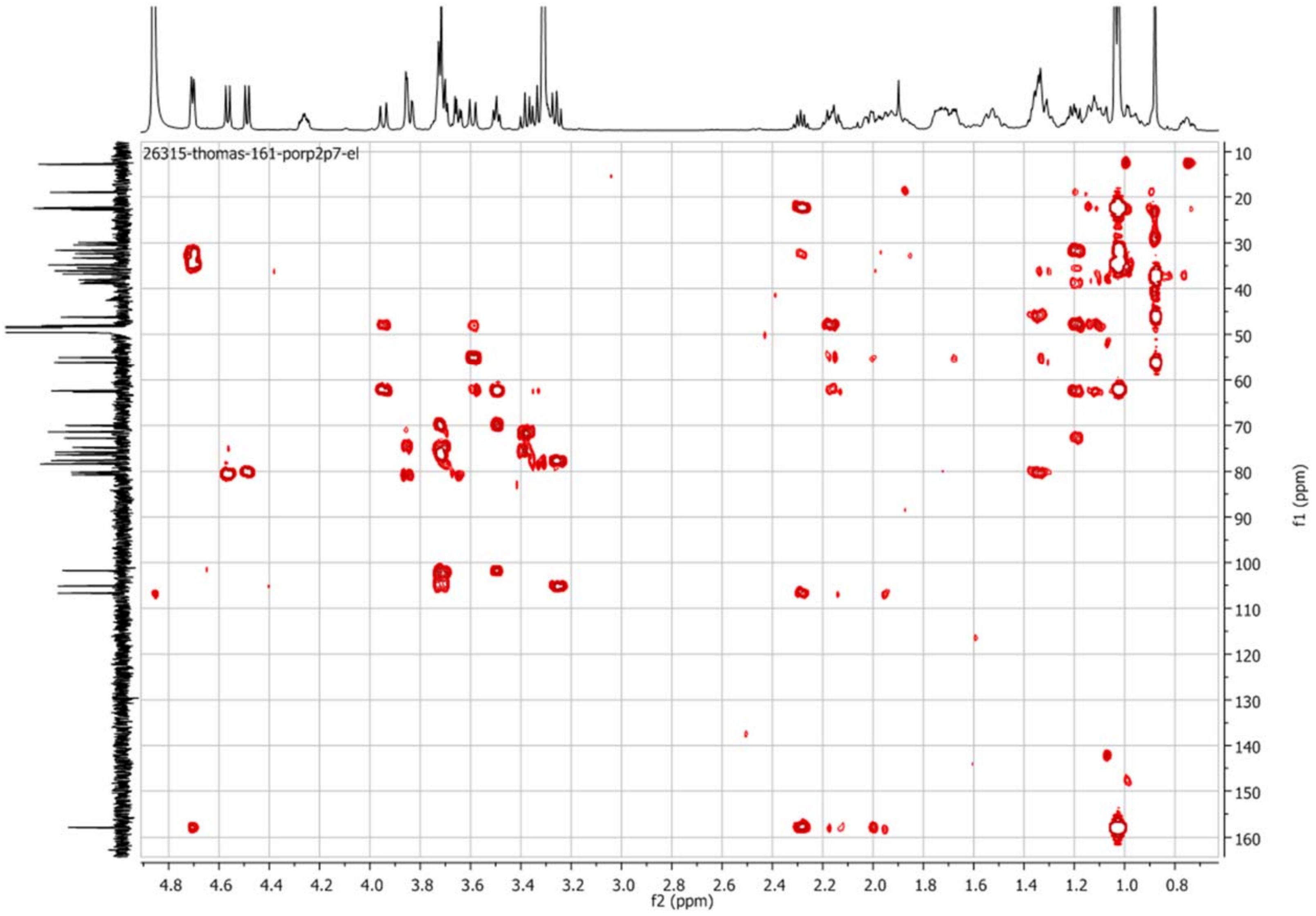

Figure S8. HMBC spectrum of **1** at 500 MHz in CD_3OD

Compound Formula	Name	RT	Algorithm
$C_{41}H_{70}O_{13}$	**Poecillastroside B**	6.581	Spectrum Extraction

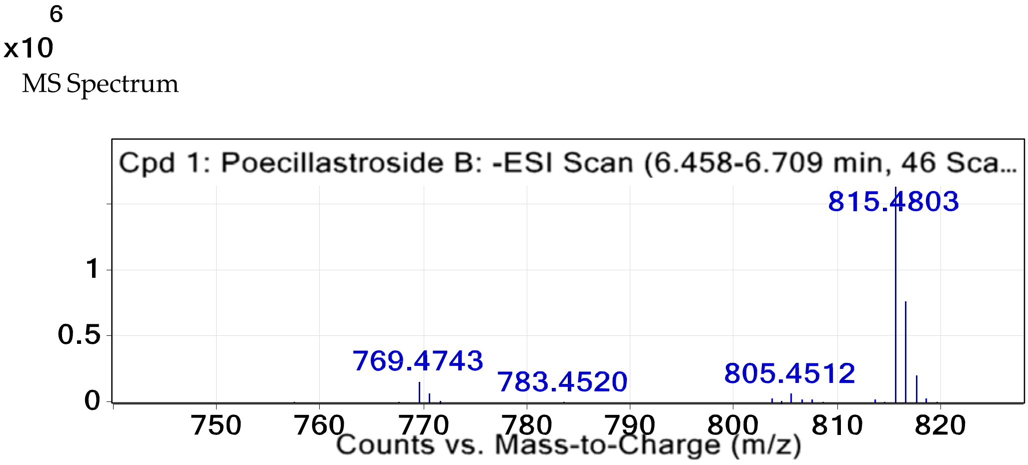

Figure S9. (–)-HRESIMS analysis of **2.**

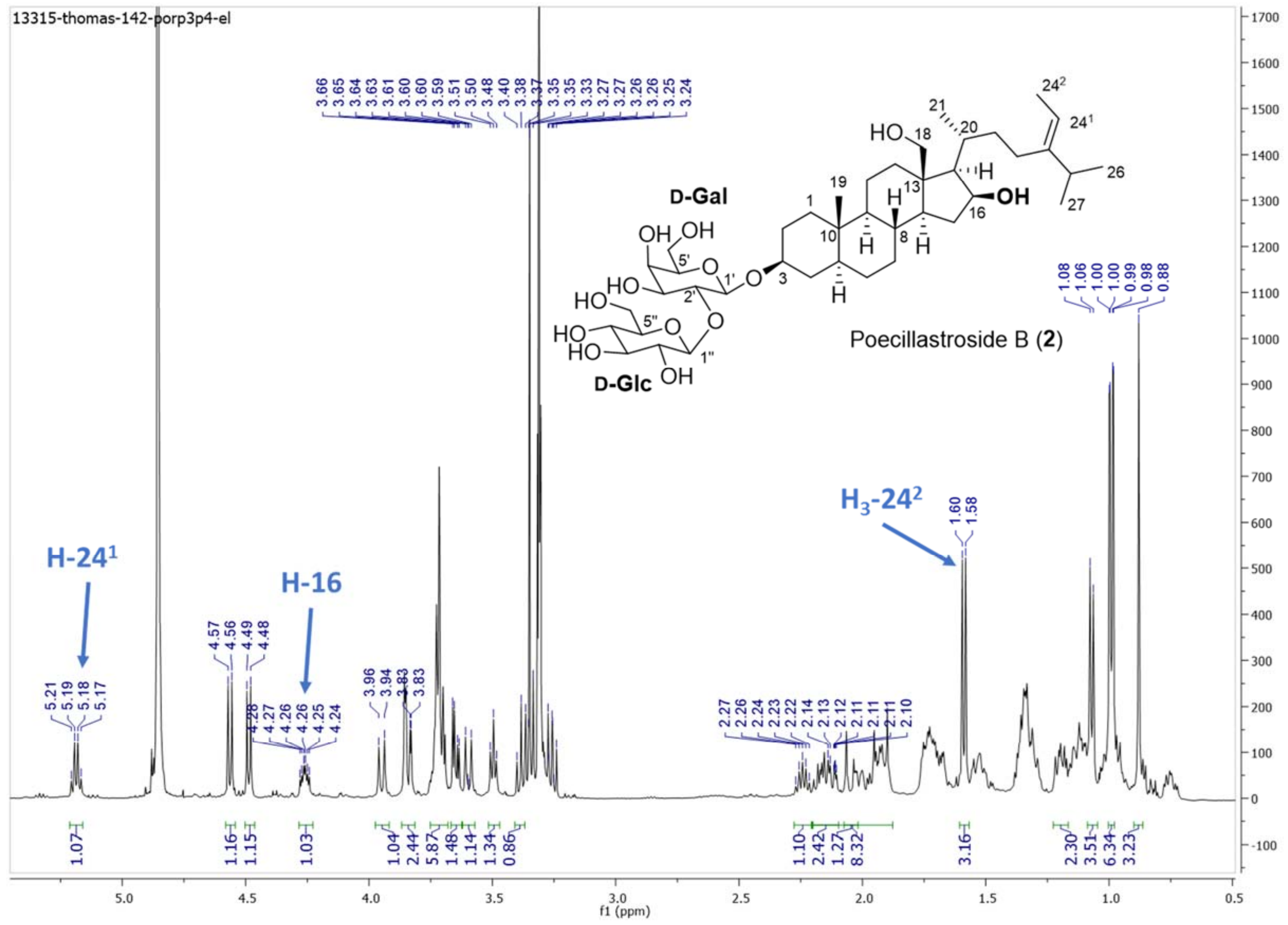

Figure S10. ^{1}H NMR spectrum of **2** at 500 MHz in CD_3OD

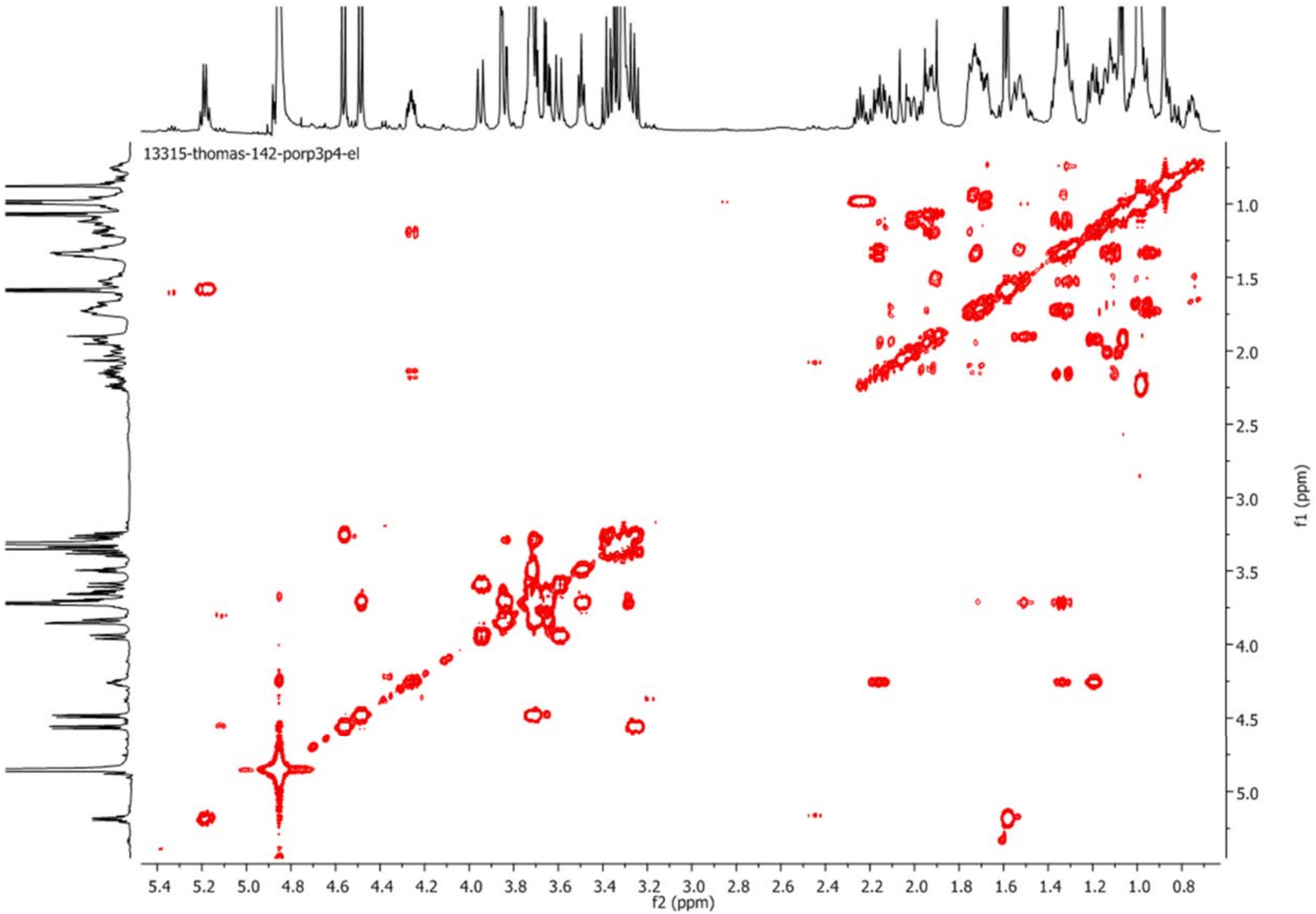

Figure S11. COSY NMR spectrum of **2** at 500 MHz in CD_3OD

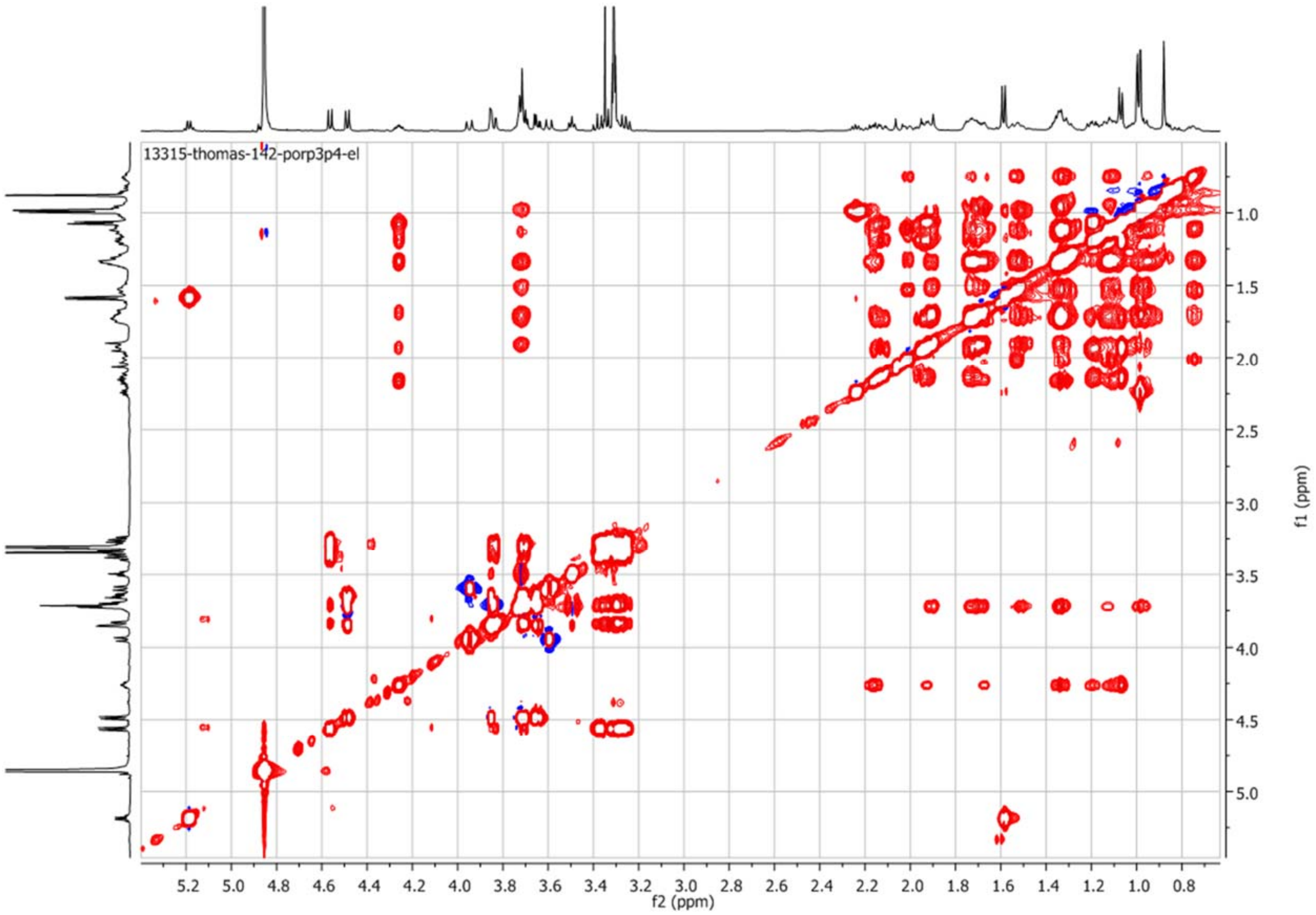

Figure S12. TOCSY NMR spectrum of **2** at 500 MHz in CD_3OD

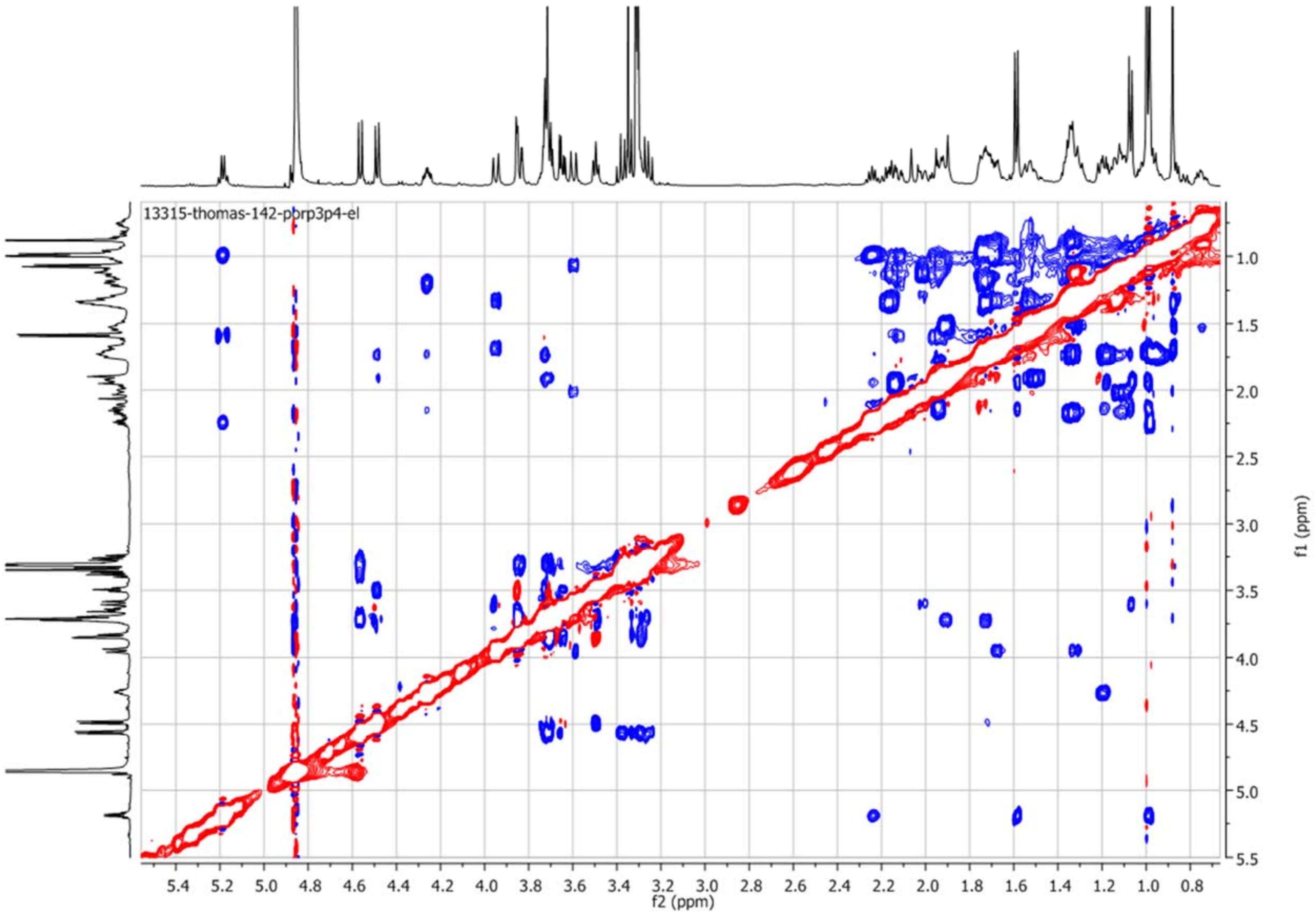

Figure S13. NOESY NMR spectrum of **2** at 500 MHz in CD_3OD

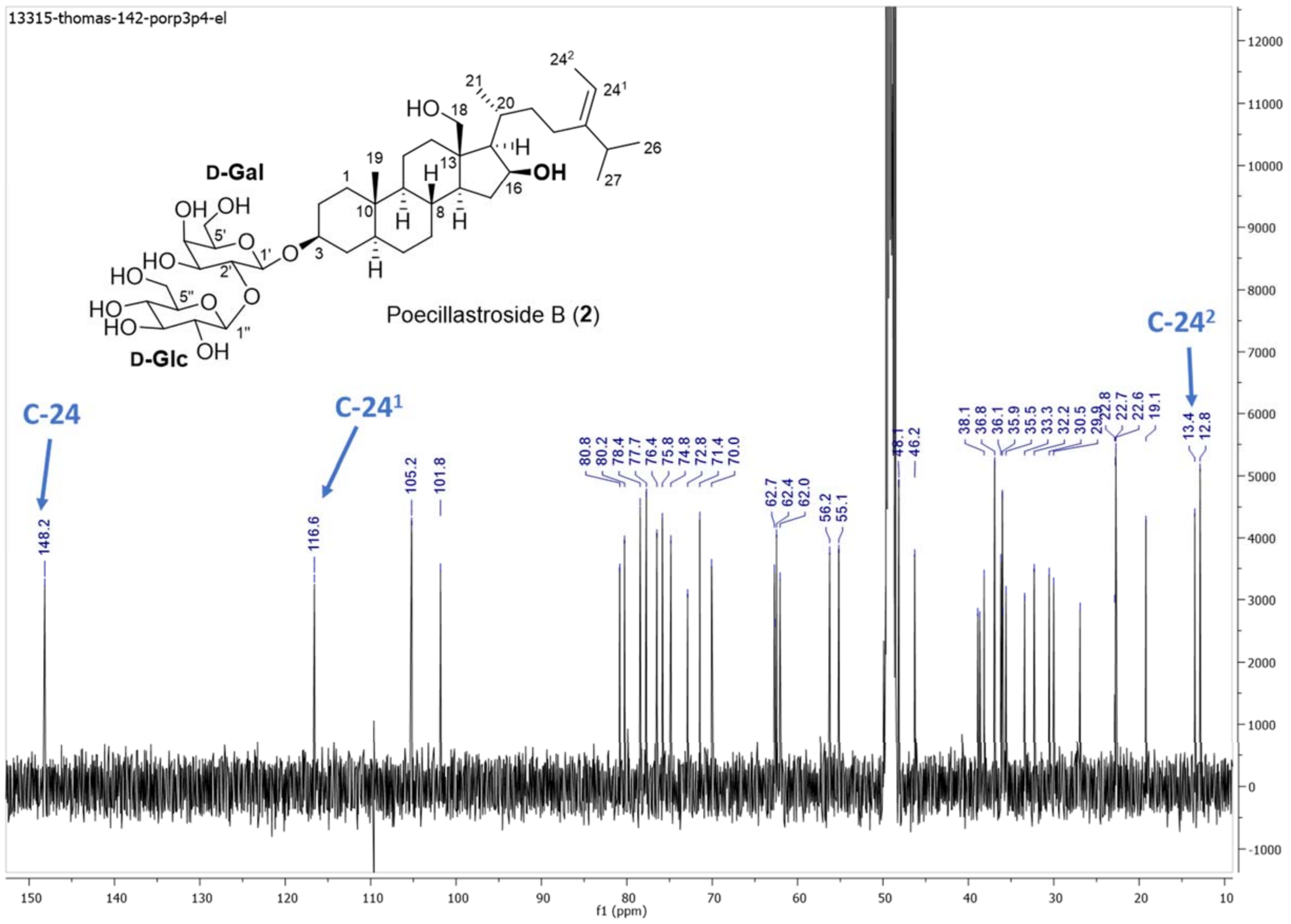

Figure S14. ^{13}C NMR spectrum of **2** at 125 MHz in CD_3OD

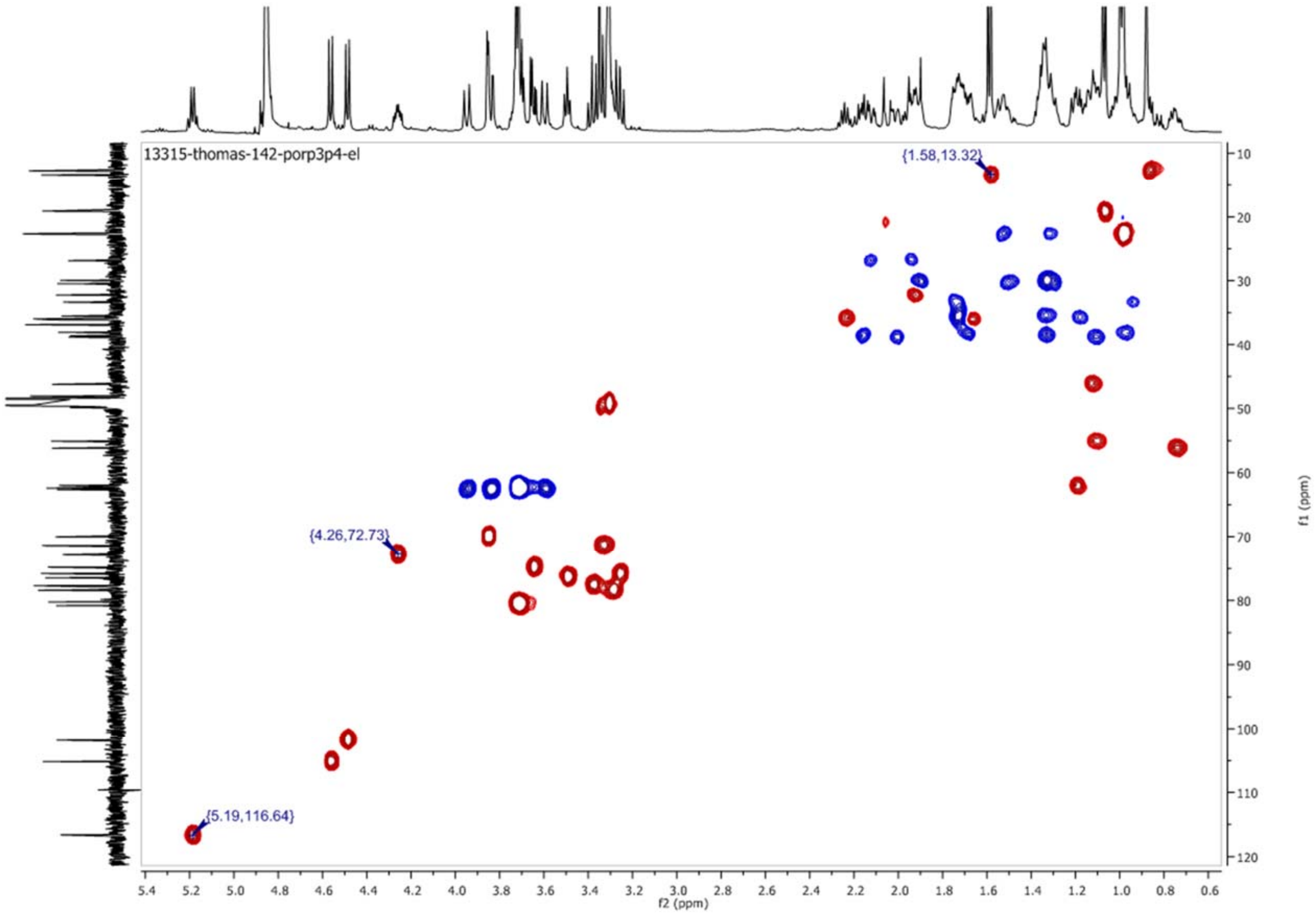

Figure S15. HSQC NMR spectrum of **2** at 500 MHz in CD_3OD

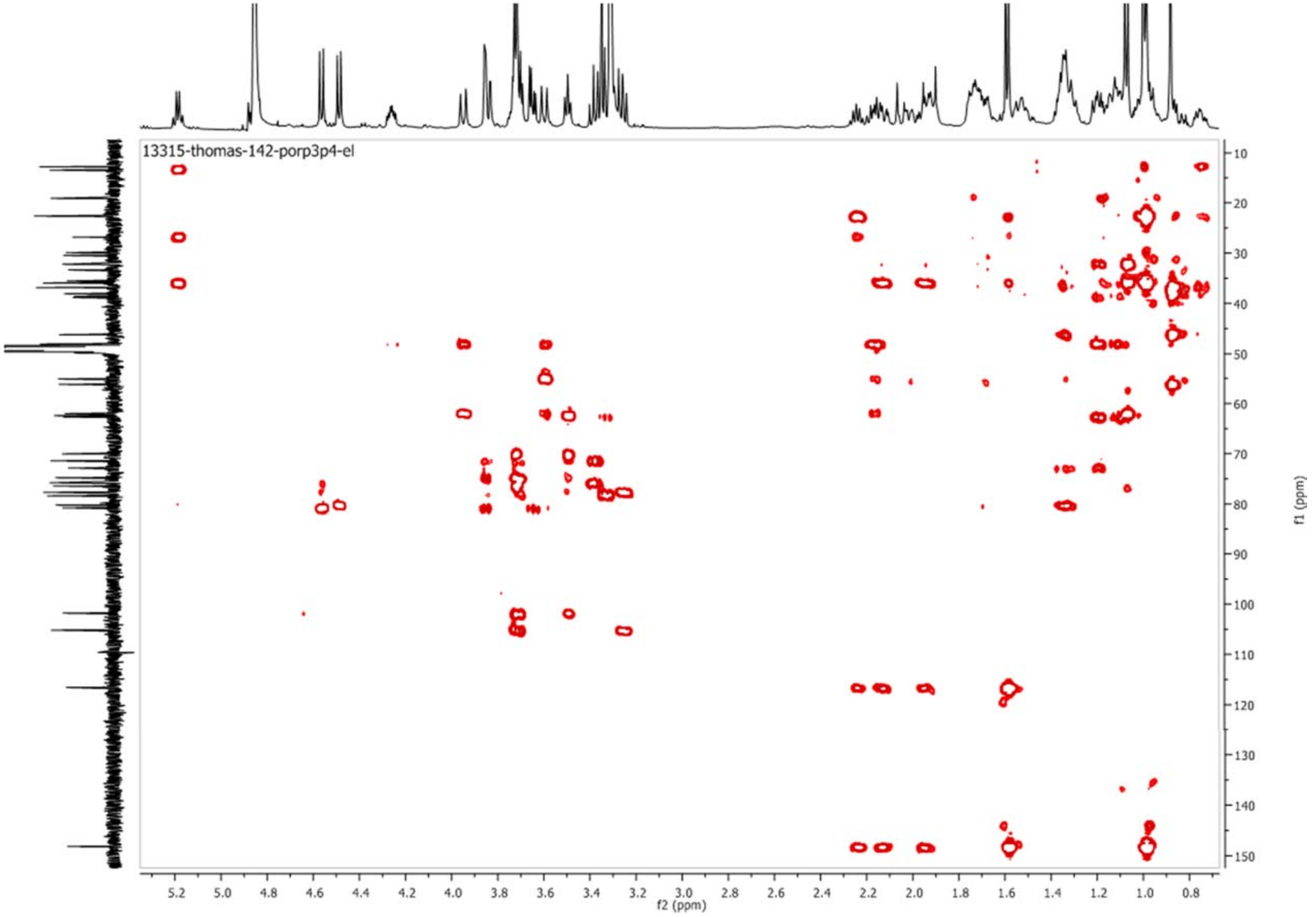

Figure S16. HMBC spectrum of **2** at 500 MHz in CD_3OD

Compound Formula	Name	RT	Algorithm
$C_{41}H_{70}O_{13}$	**Poecillastroside C**	6.559	Spectrum Extraction

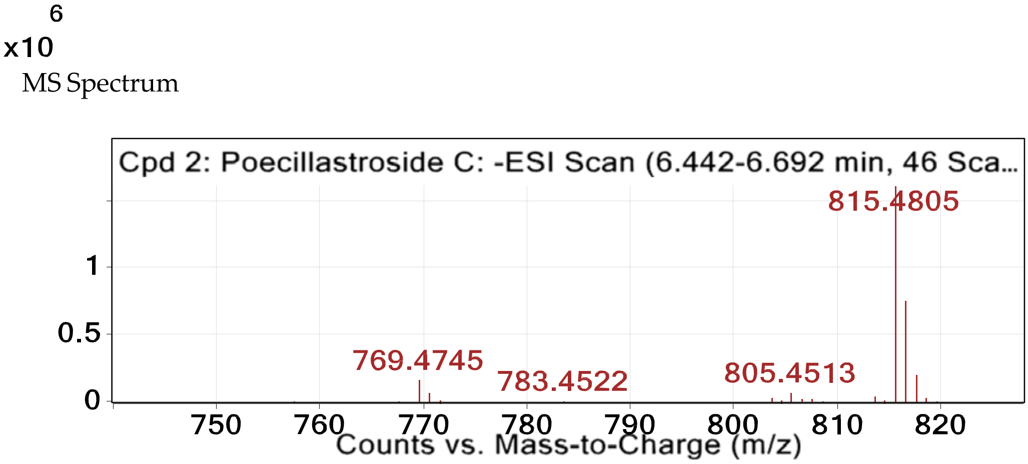

Figure S17. (–)-HRESIMS analysis of **3.**

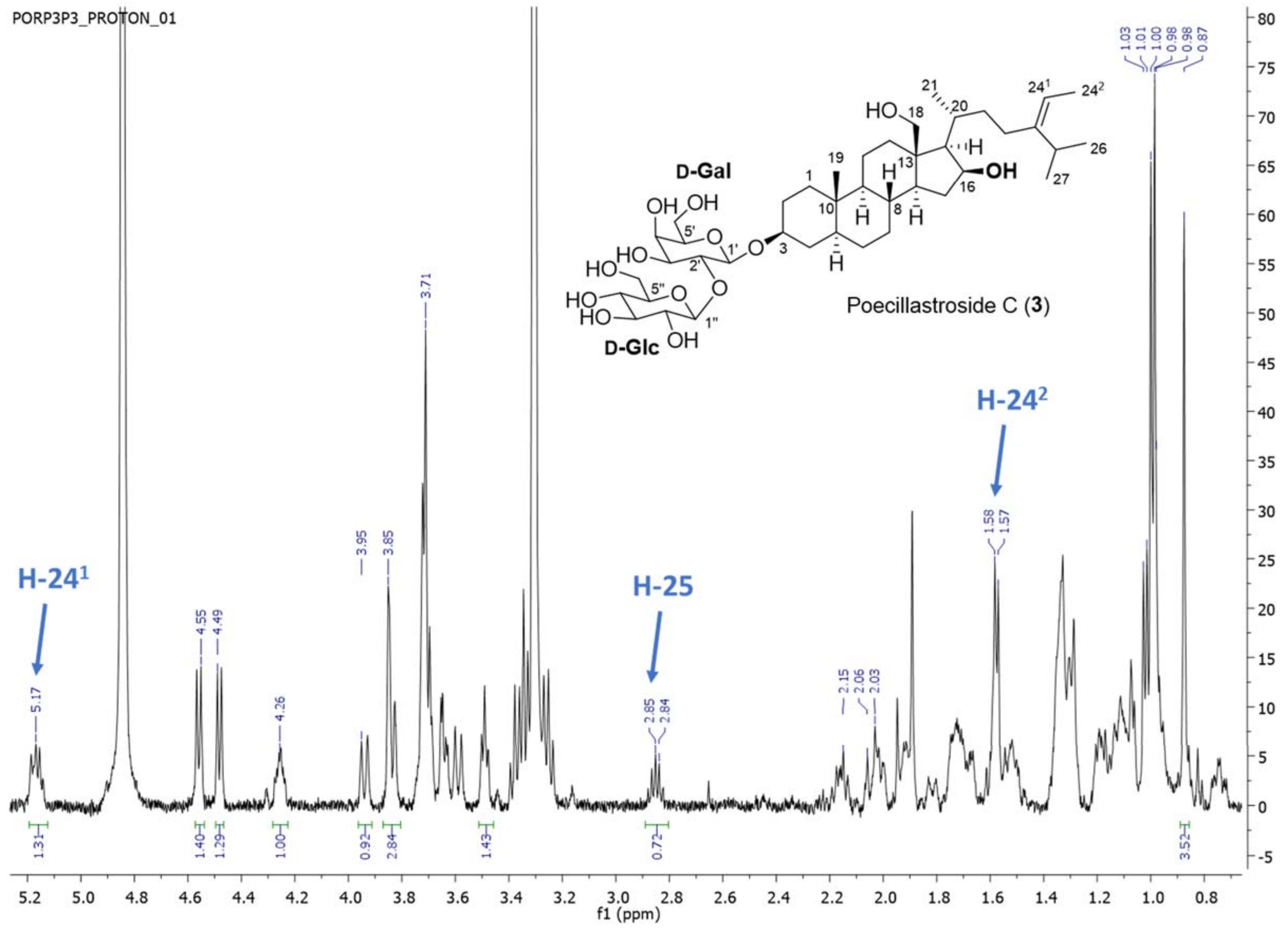

Figure S18. ^{1}H NMR spectrum of **3** at 500 MHz in CD_3OD

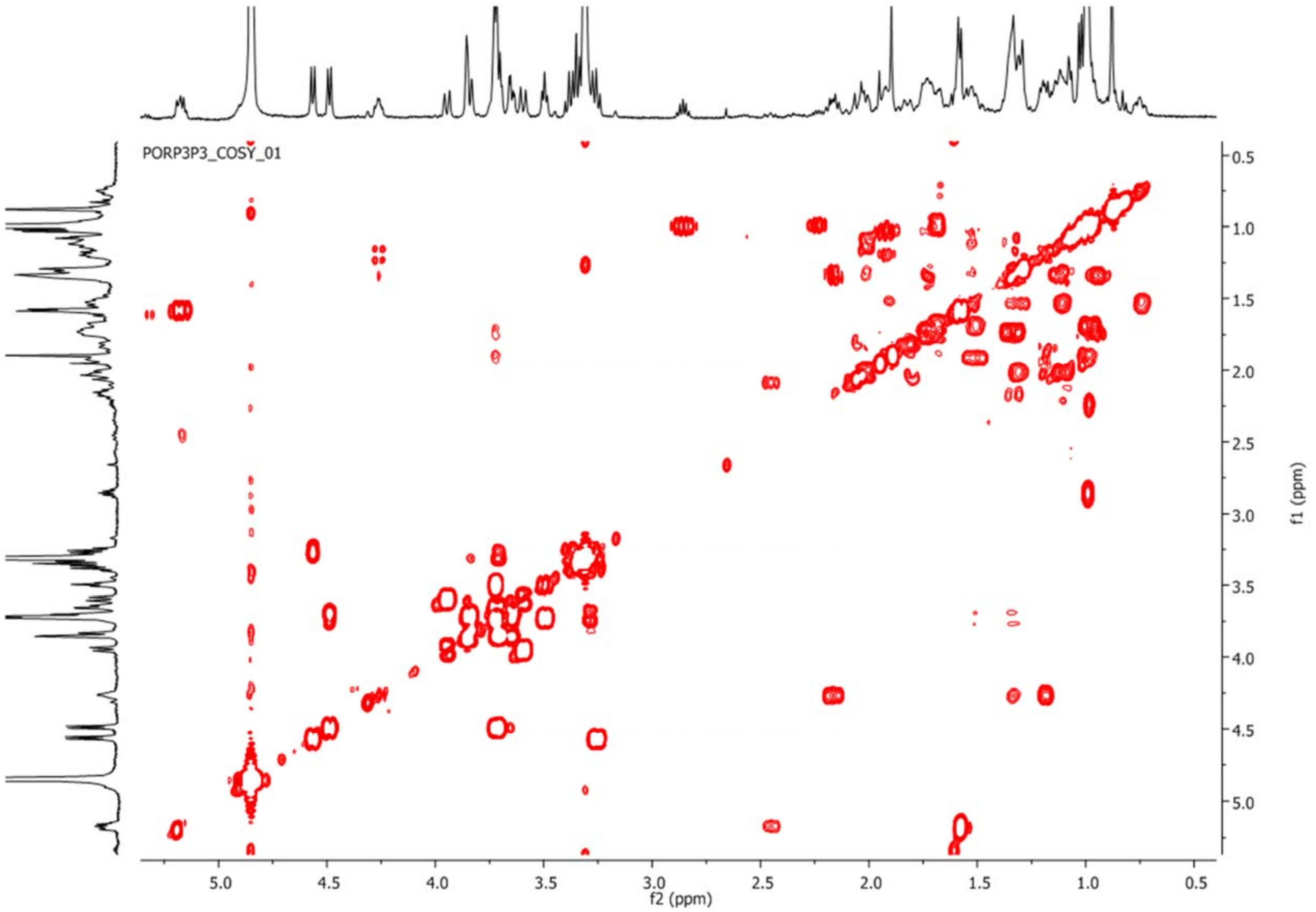

Figure S19. COSY NMR spectrum of **3** at 500 MHz in CD_3OD

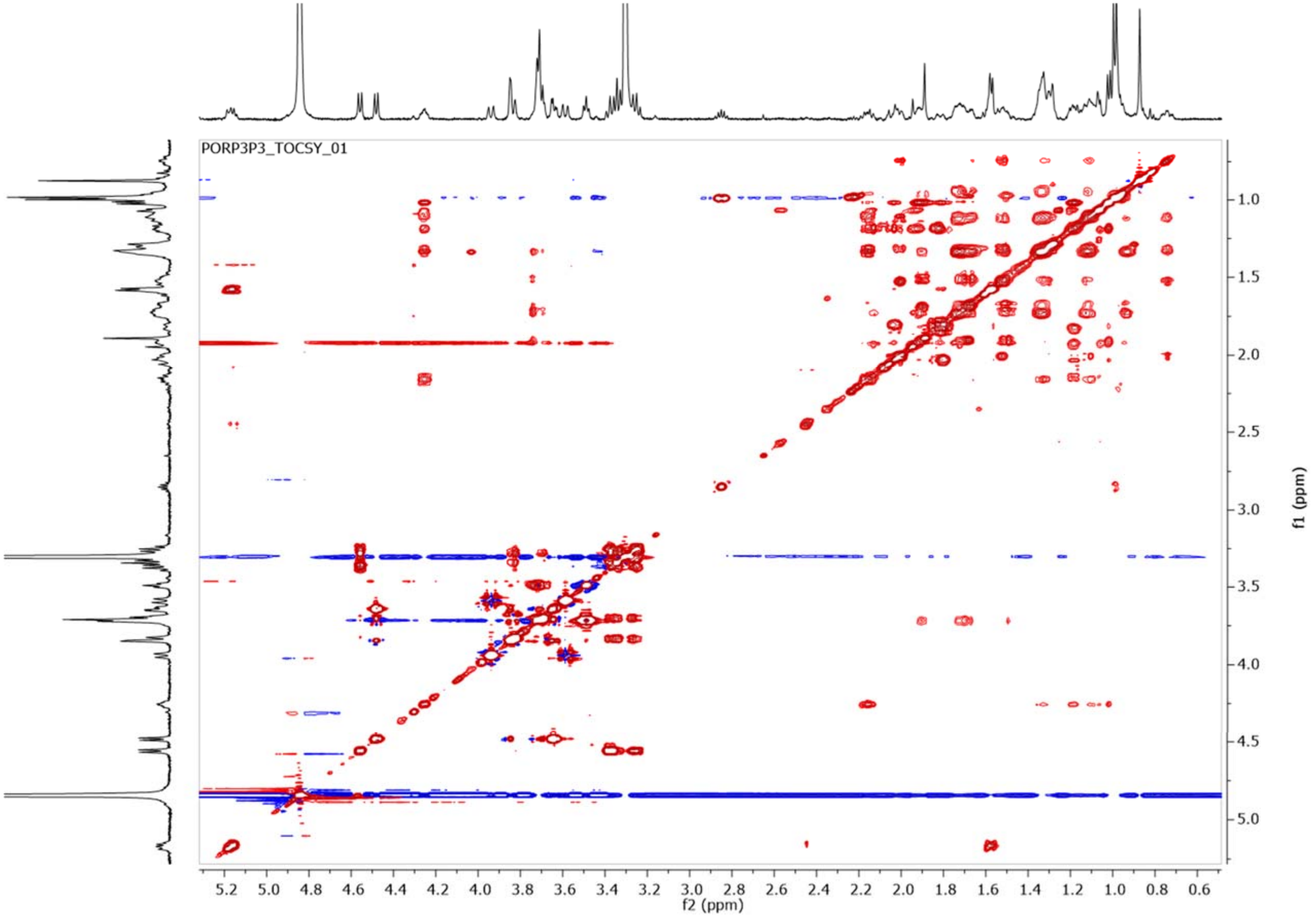

Figure S20. TOCSY NMR spectrum of **3** at 500 MHz in CD_3OD

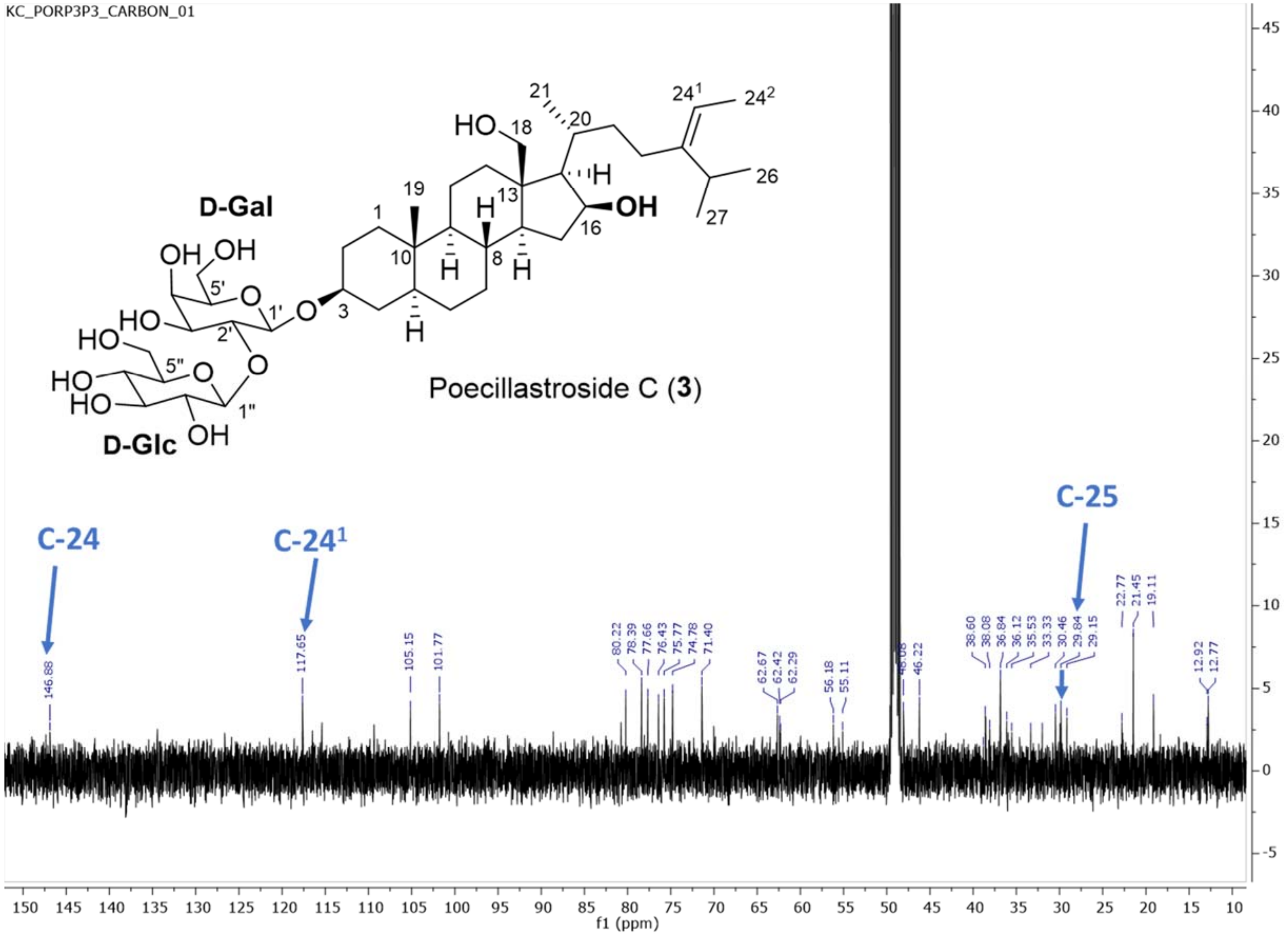

Figure S21. ^{13}C NMR spectrum of **3** at 500 MHz in CD_3OD

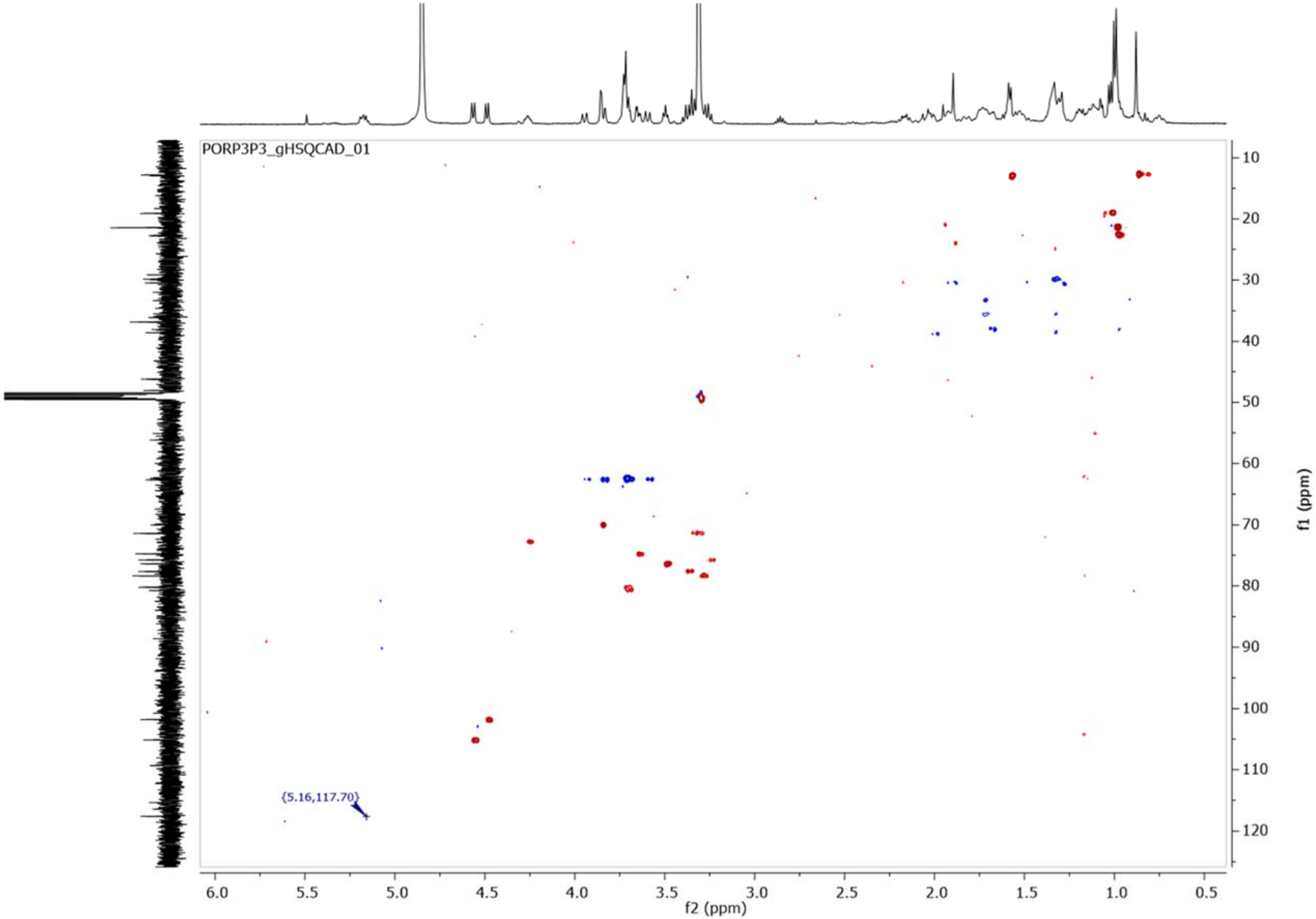

Figure S22. HSQC NMR spectrum of **3** at 500 MHz in CD_3OD

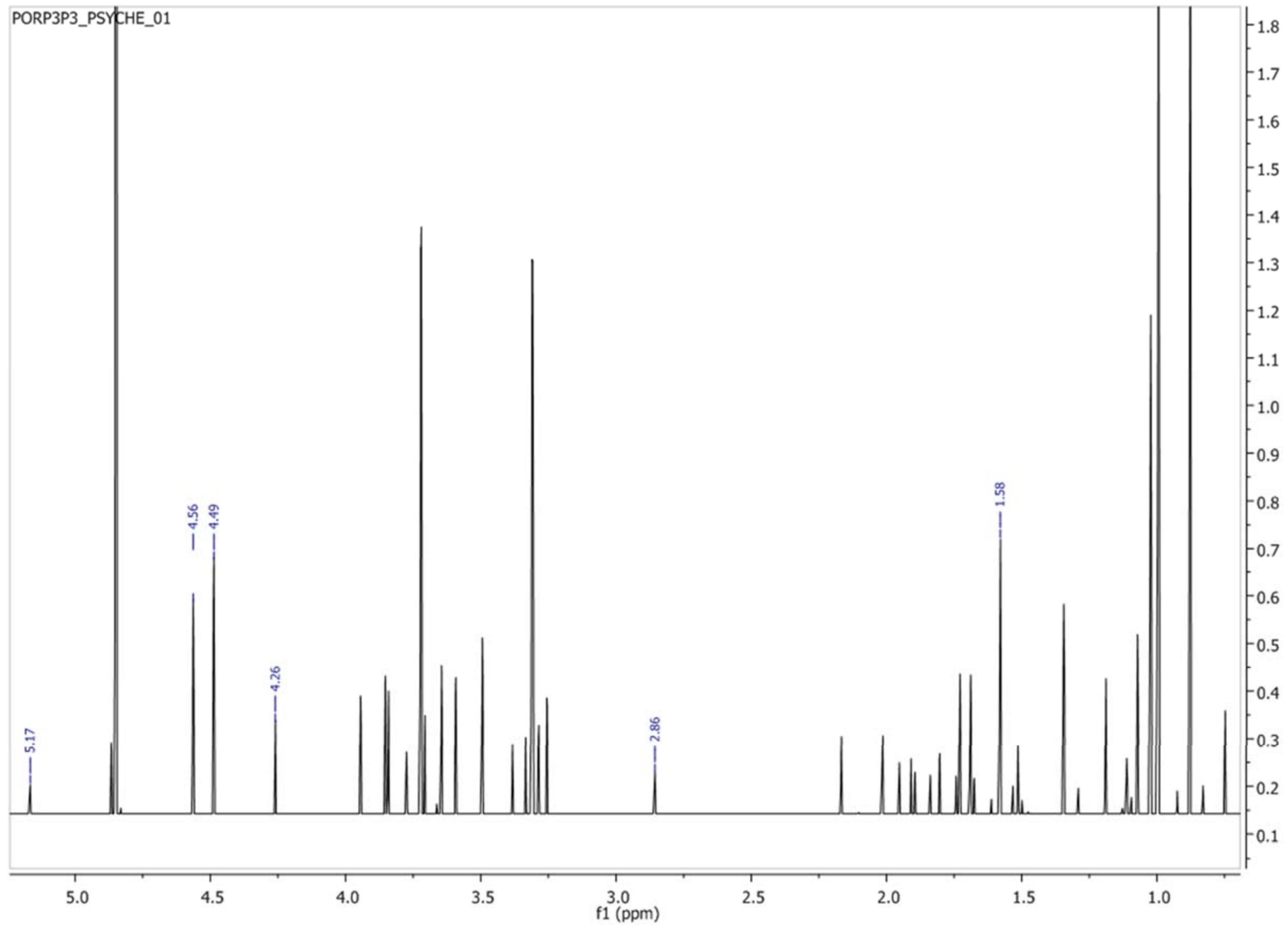

Figure S23. PSYCHE_1D NMR spectrum of **3** at 500 MHz in CD_3OD

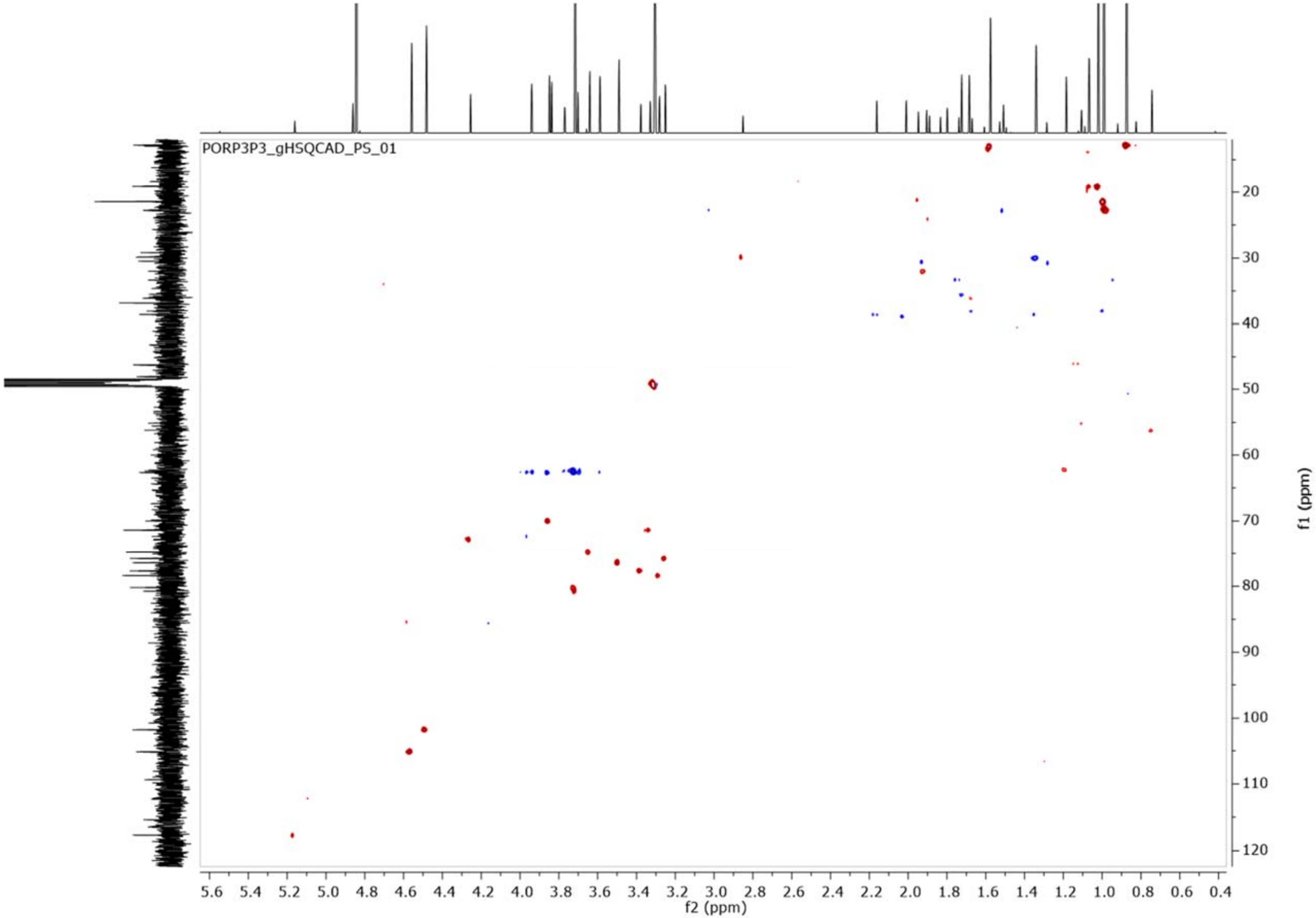

Figure S24. PS-HSQC NMR spectrum of **3** at 500 MHz in CD_3OD

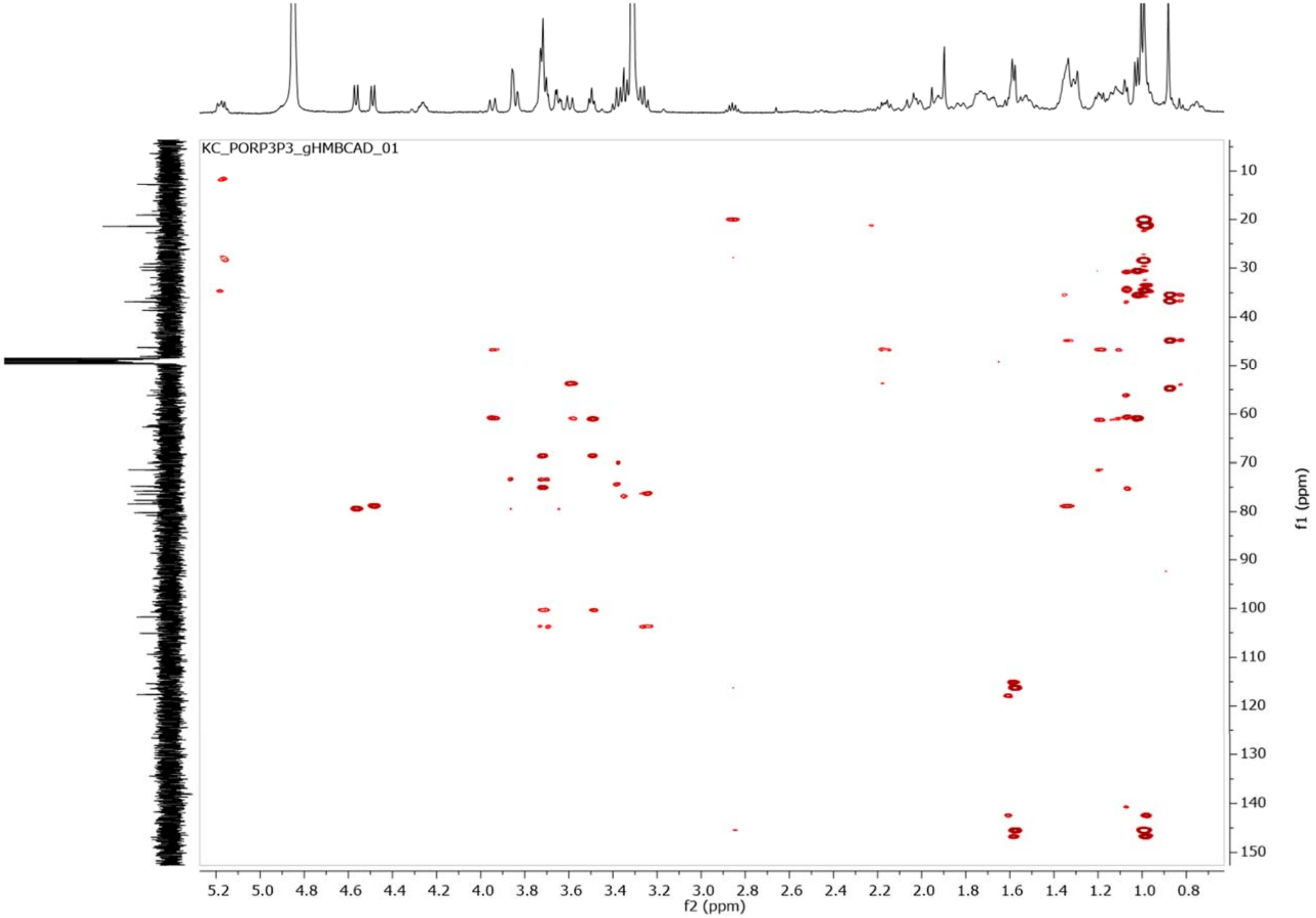

Figure S25. HMBC spectrum of **3** at 500 MHz in CD_3OD

Compound Formula	Name	RT	Algorithm
$C_{41}H_{68}O_{13}$	**Poecillastroside D**	7.049	Spectrum Extraction

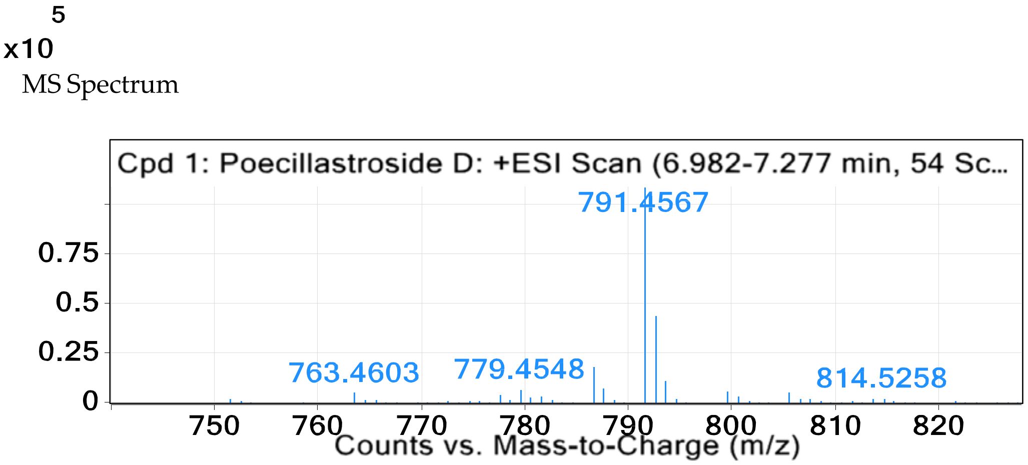

Figure S26. (+)-HRESIMS analysis of **4.**

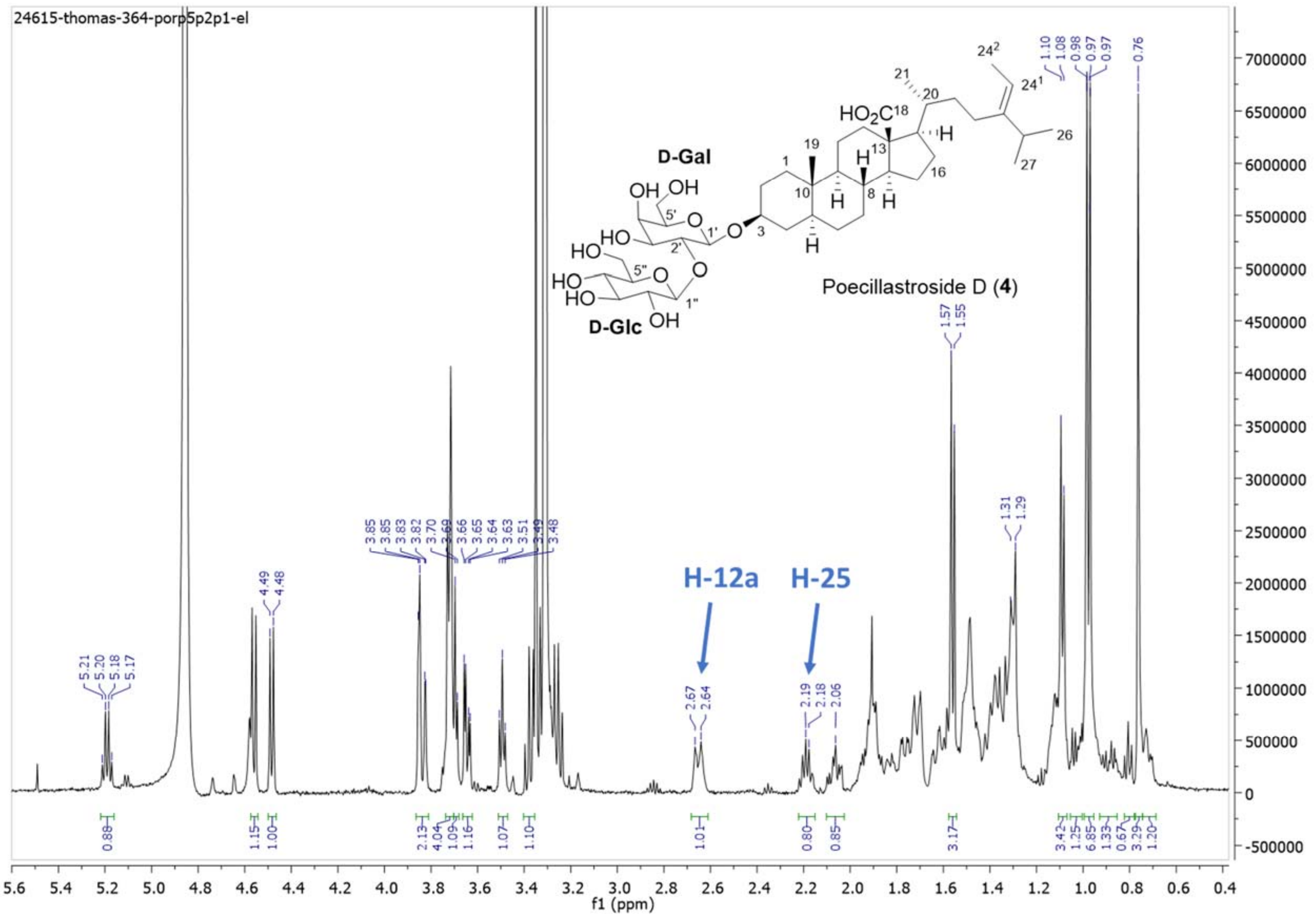

Figure S27. ^{1}H NMR spectrum of **4** at 500 MHz in CD_3OD

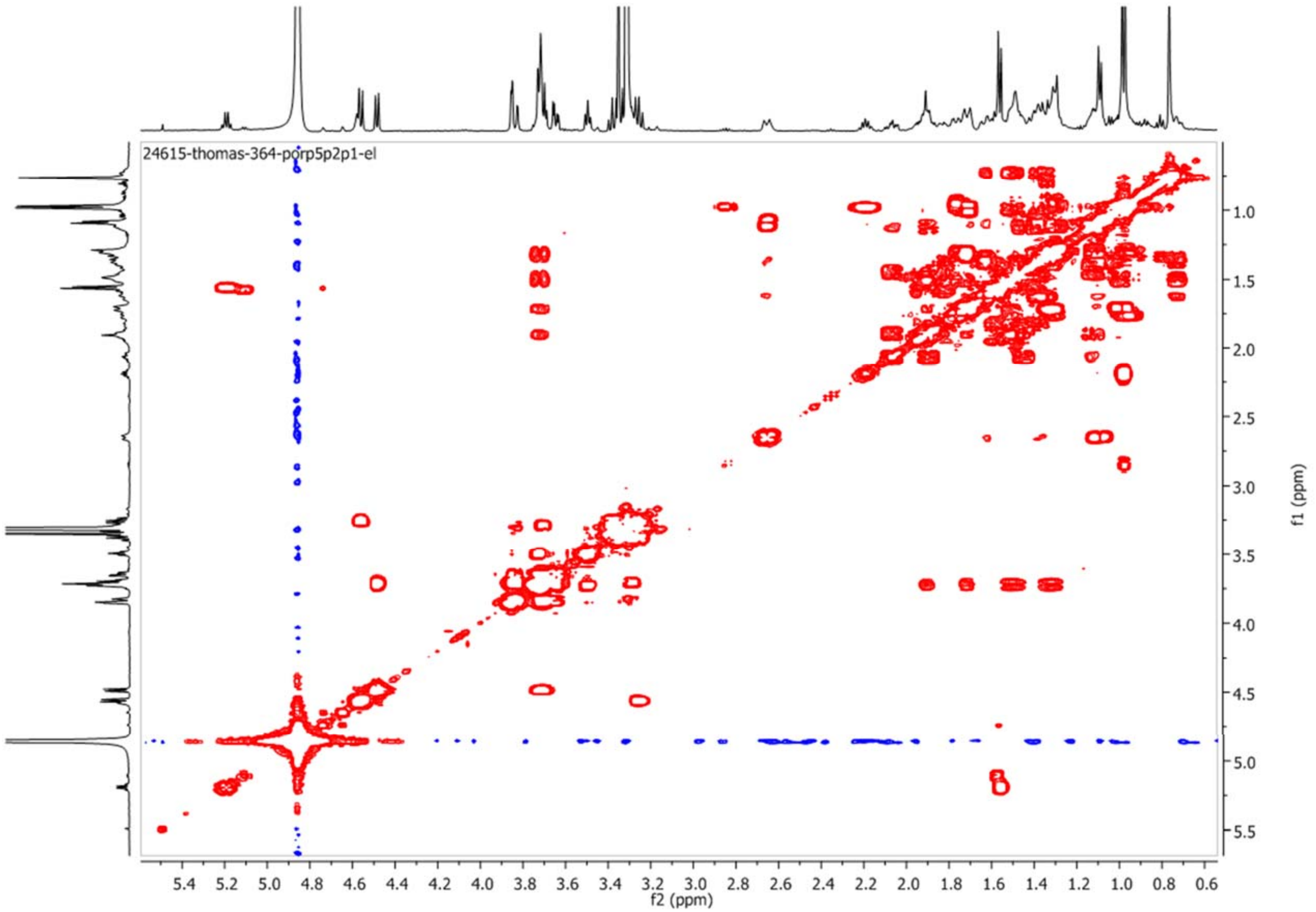

Figure S28. COSY NMR spectrum of **4** at 500 MHz in CD_3OD

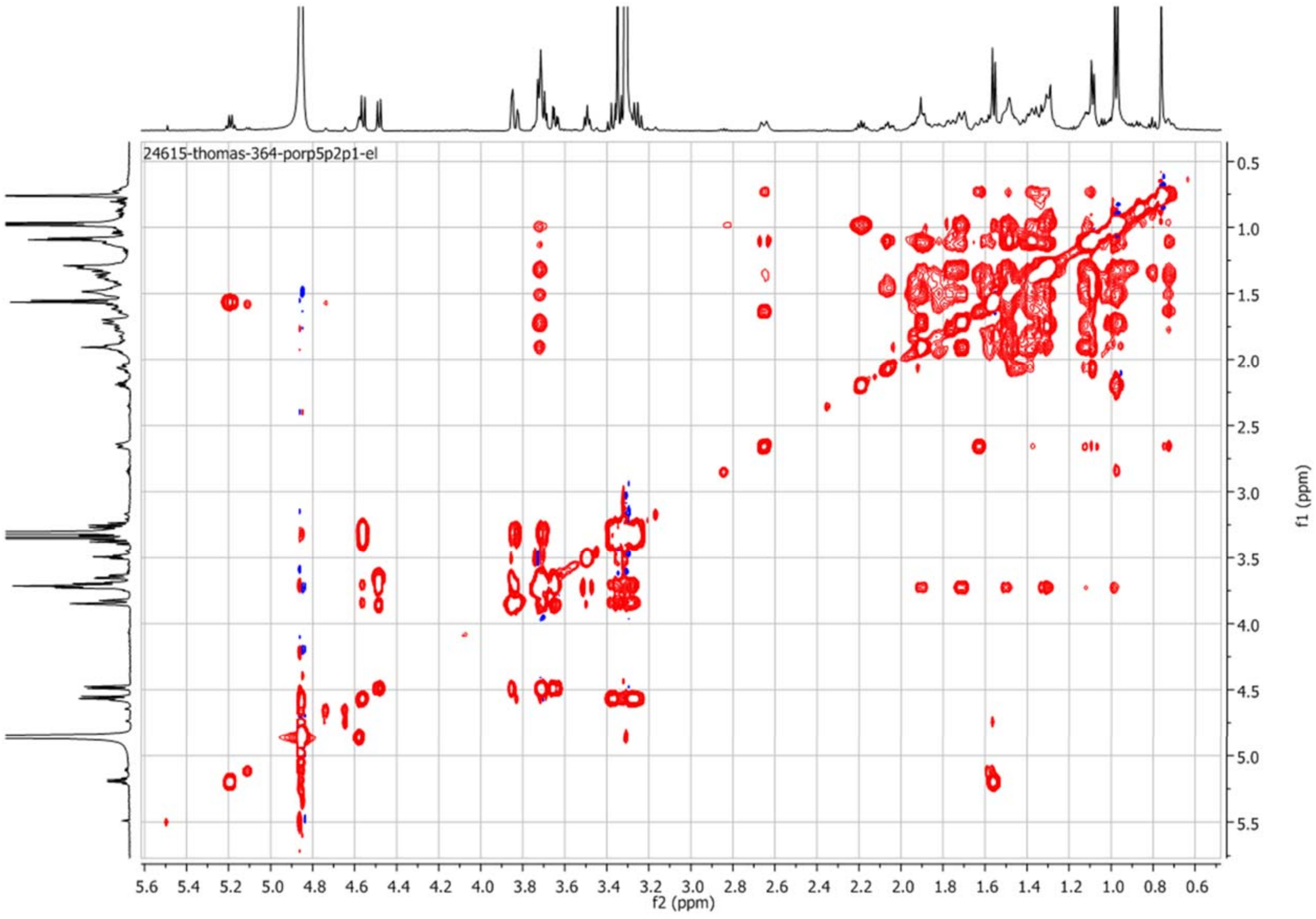

Figure S29. TOCSY NMR spectrum of **4** at 500 MHz in CD_3OD

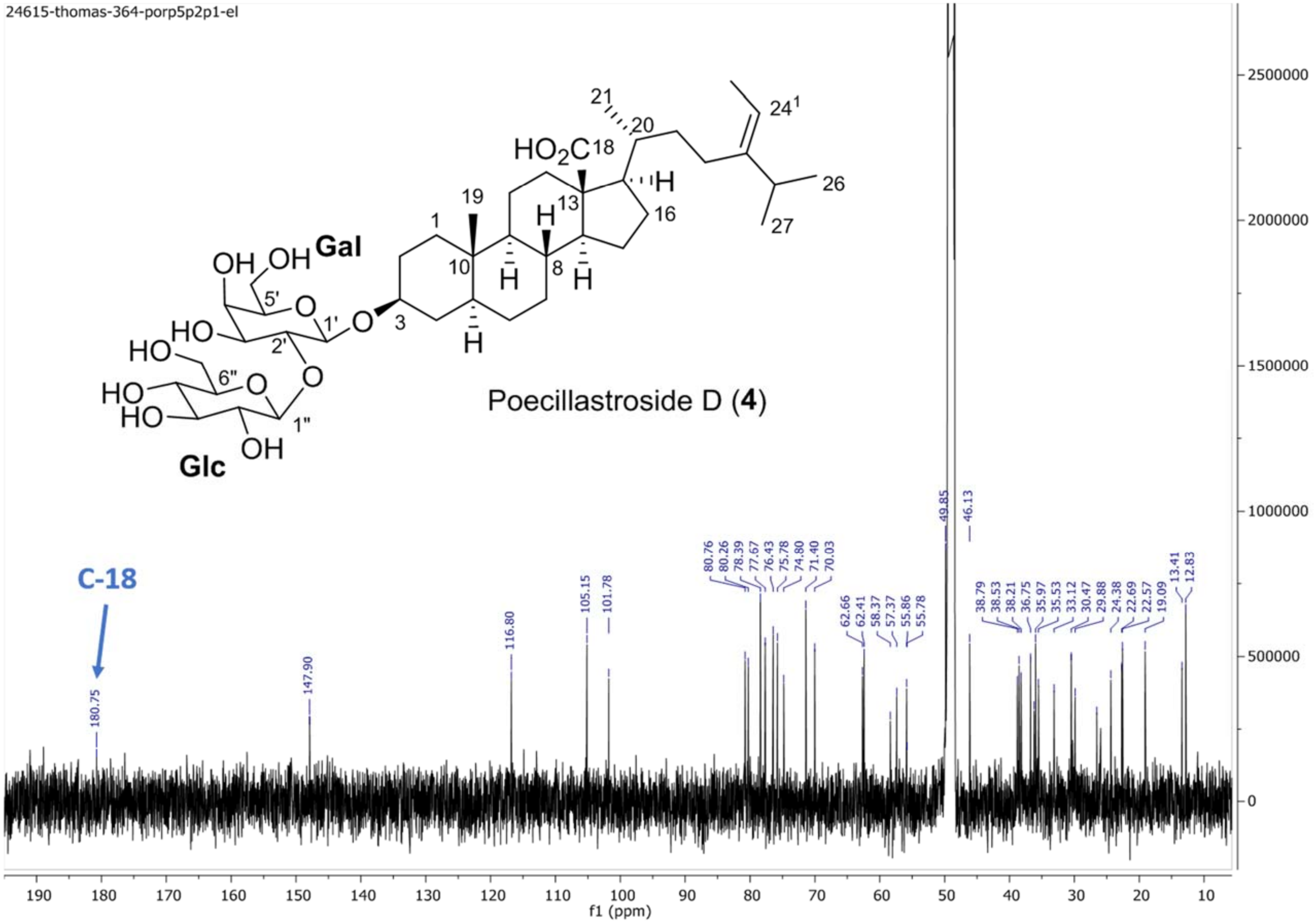

Figure S30. ^{13}C NMR spectrum of **4** at 125 MHz in CD_3OD

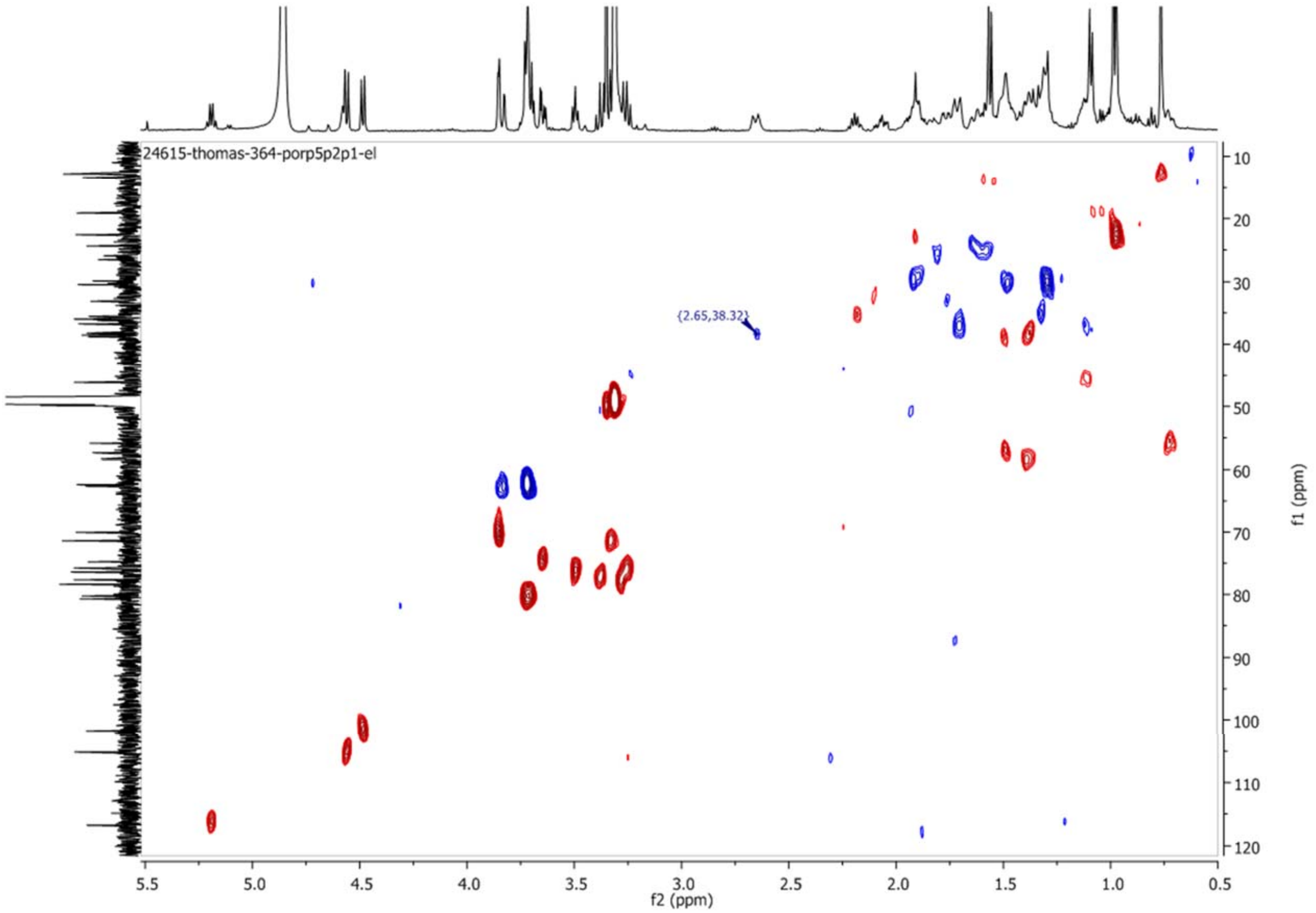

Figure S31. HSQC NMR spectrum of **4** at 500 MHz in CD_3OD

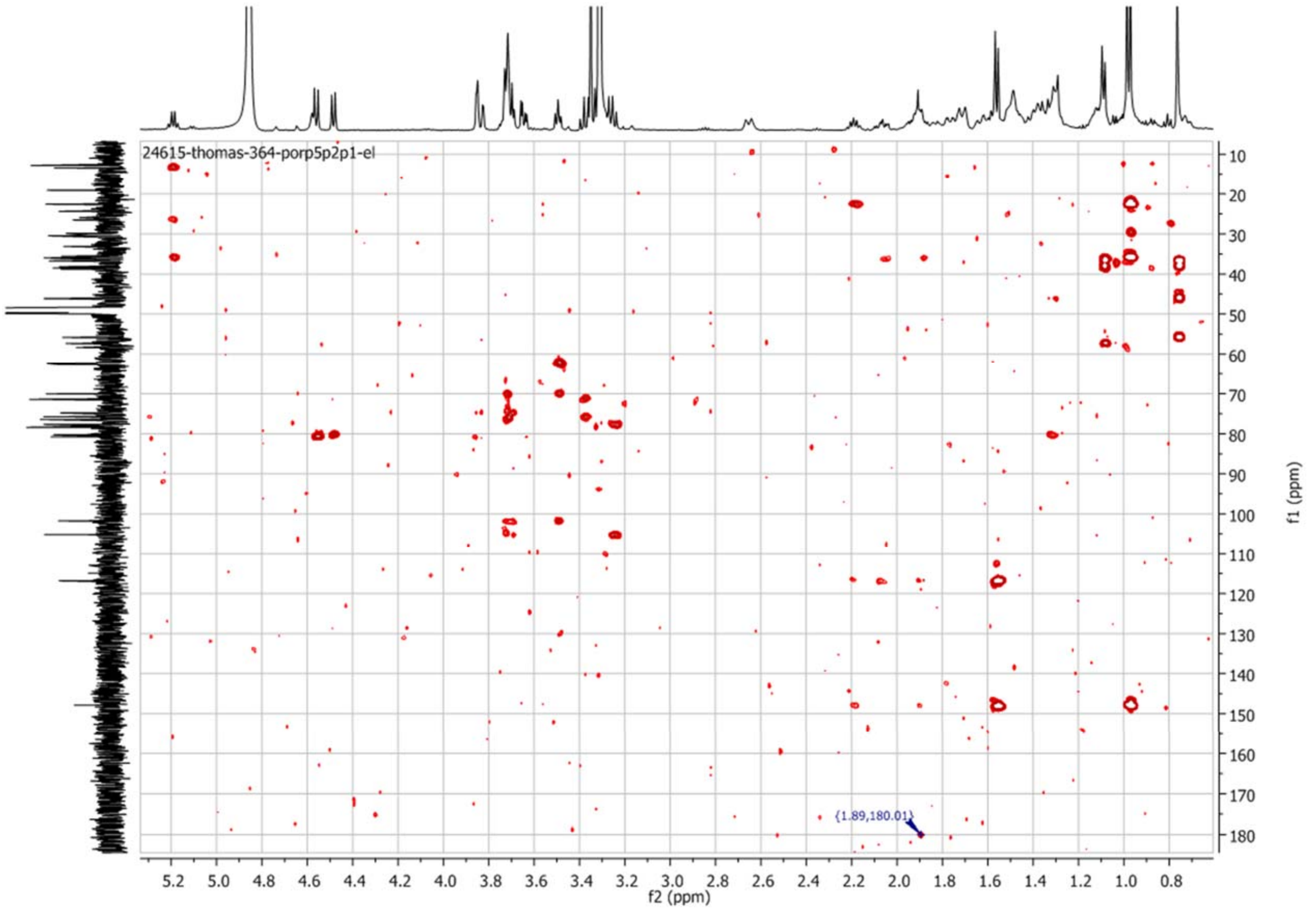

Figure S32. HMBC spectrum of **4** at 500 MHz in CD_3OD

Compound Formula	Name	RT	Algorithm
$C_{43}H_{66}O_{15}$	**Poecillastroside E**	7.109	Spectrum Extraction

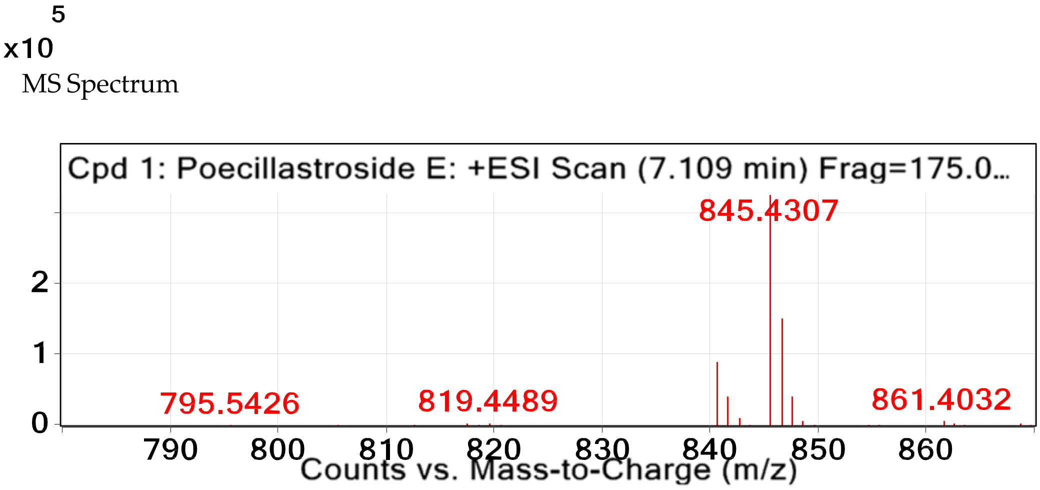

Figure S33. (+)-HRESIMS analysis of **5.**

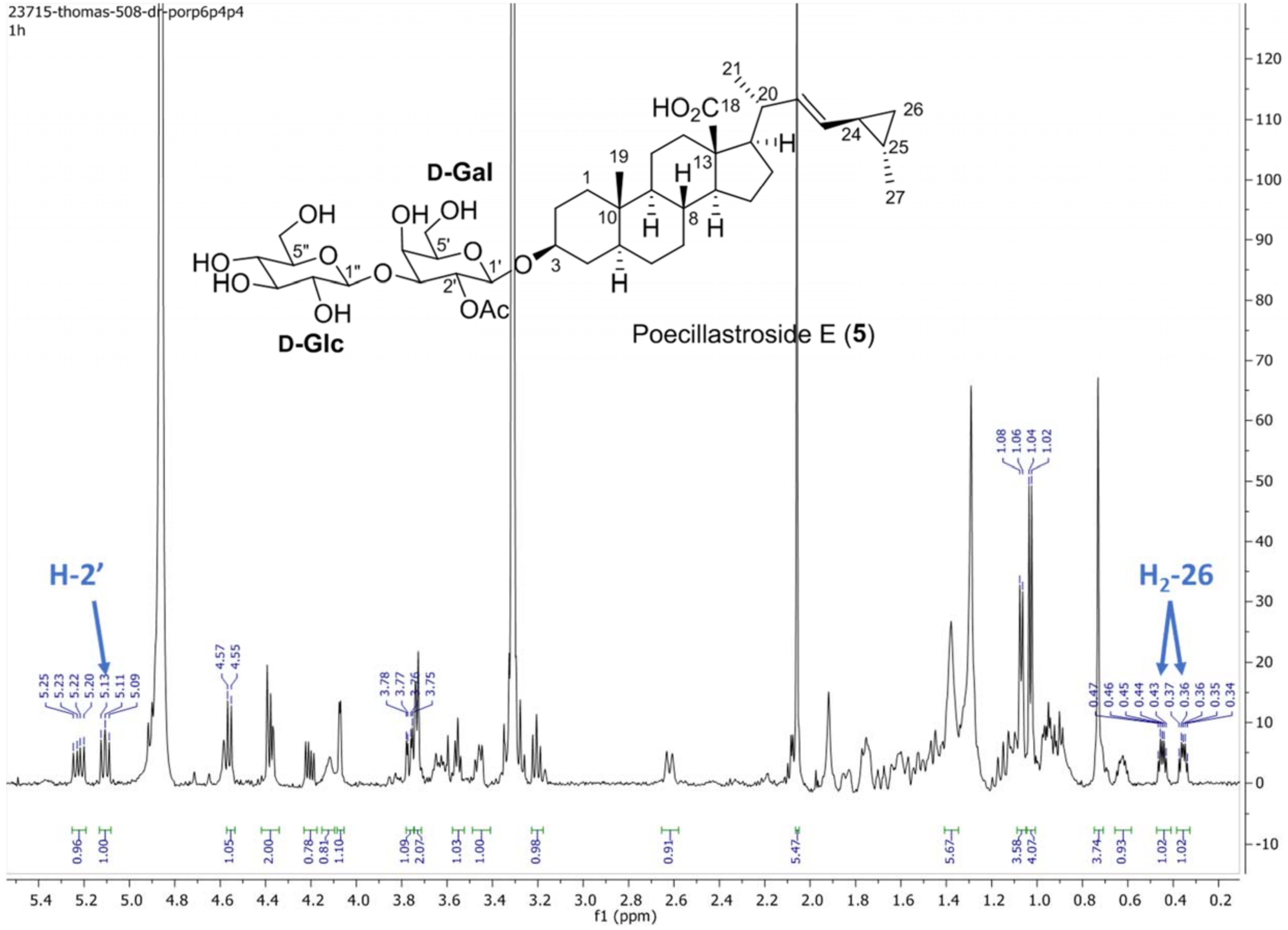

Figure S34. ^{1}H NMR spectrum of **5** at 500 MHz in CD_3OD

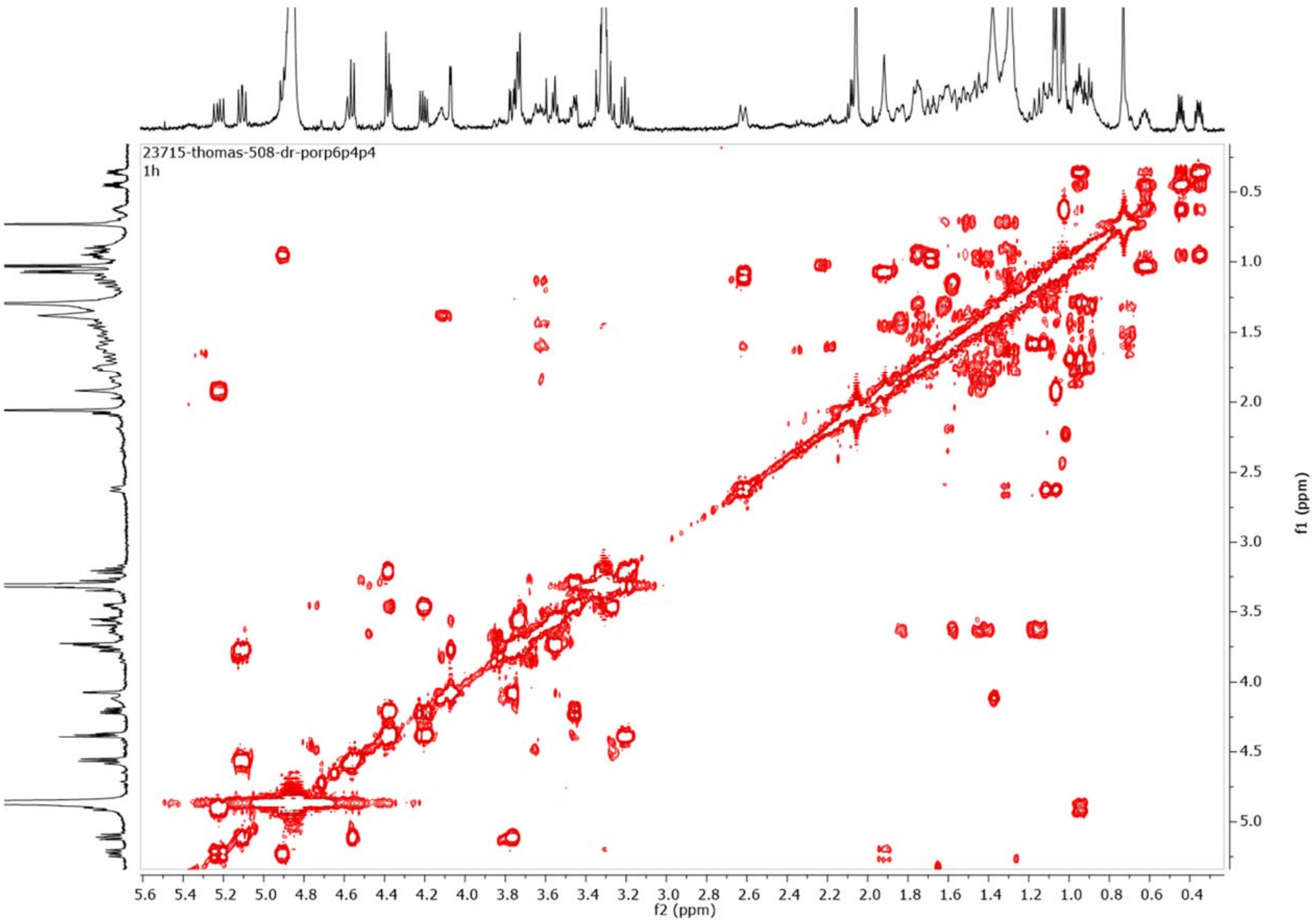

Figure S35. COSY NMR spectrum of **5** at 500 MHz in CD_3OD

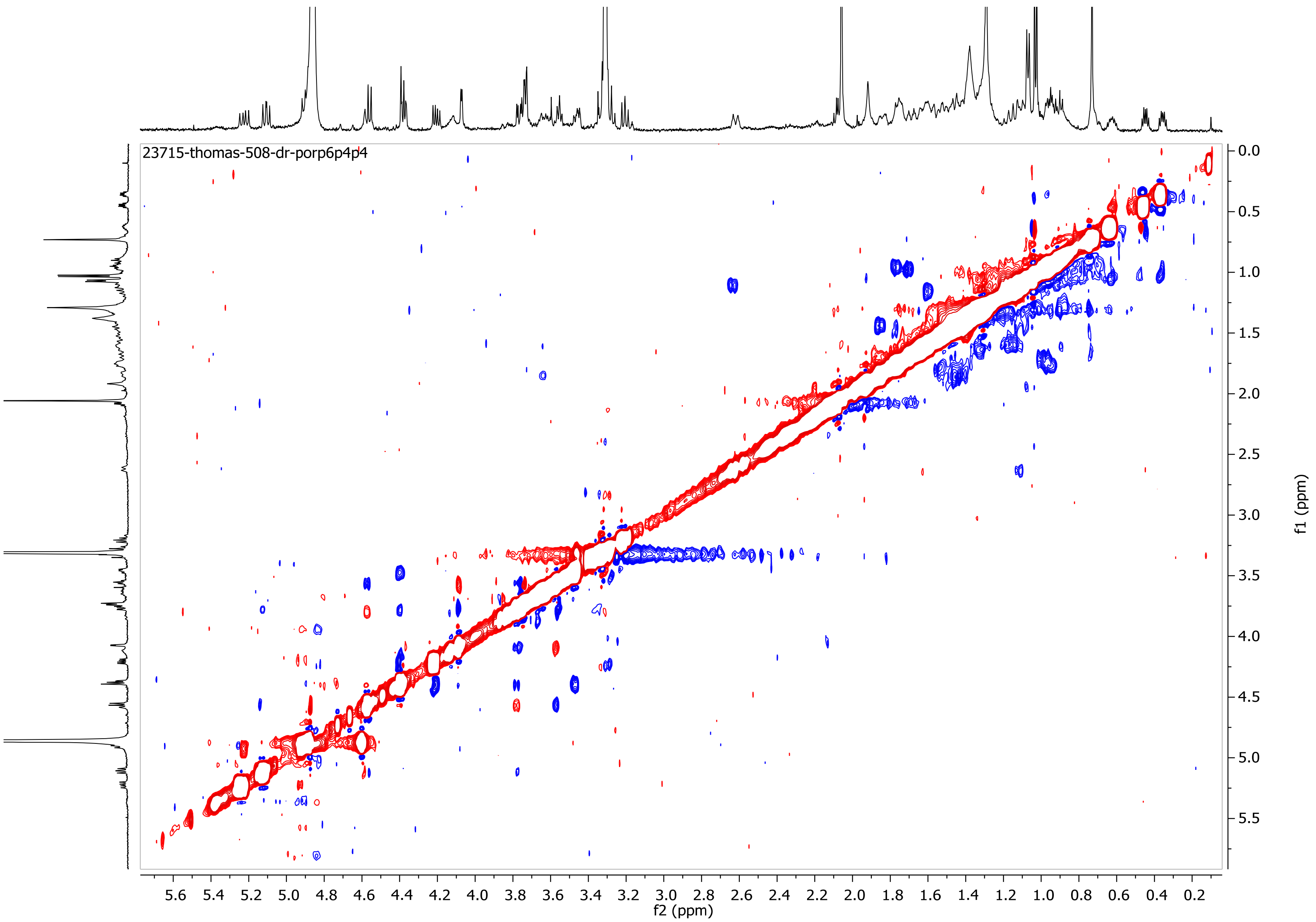

Figure S36. NOESY NMR spectrum of **5** at 500 MHz in CD_3OD

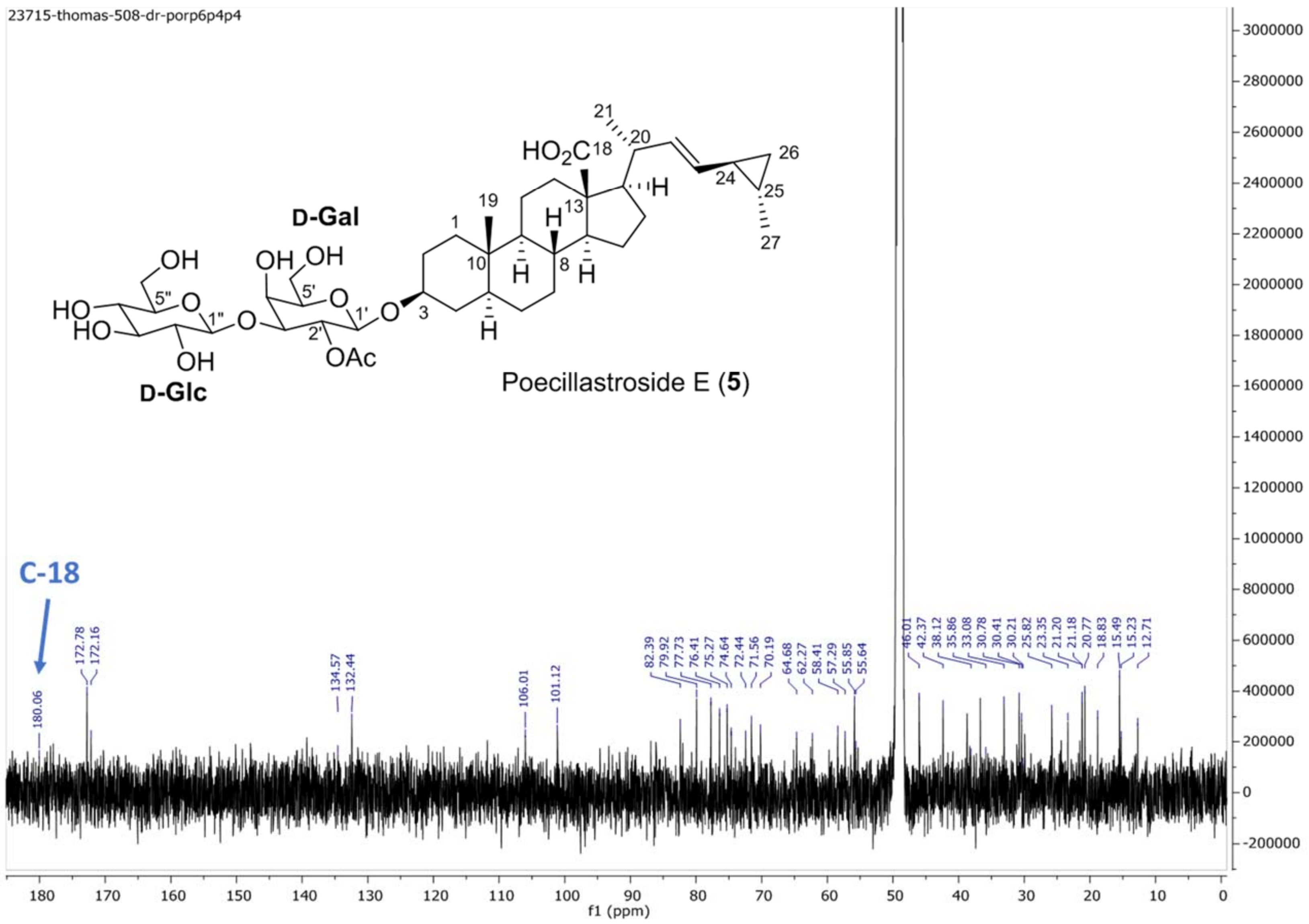

Figure S37. ^{13}C NMR spectrum of **5** at 125 MHz in CD_3OD

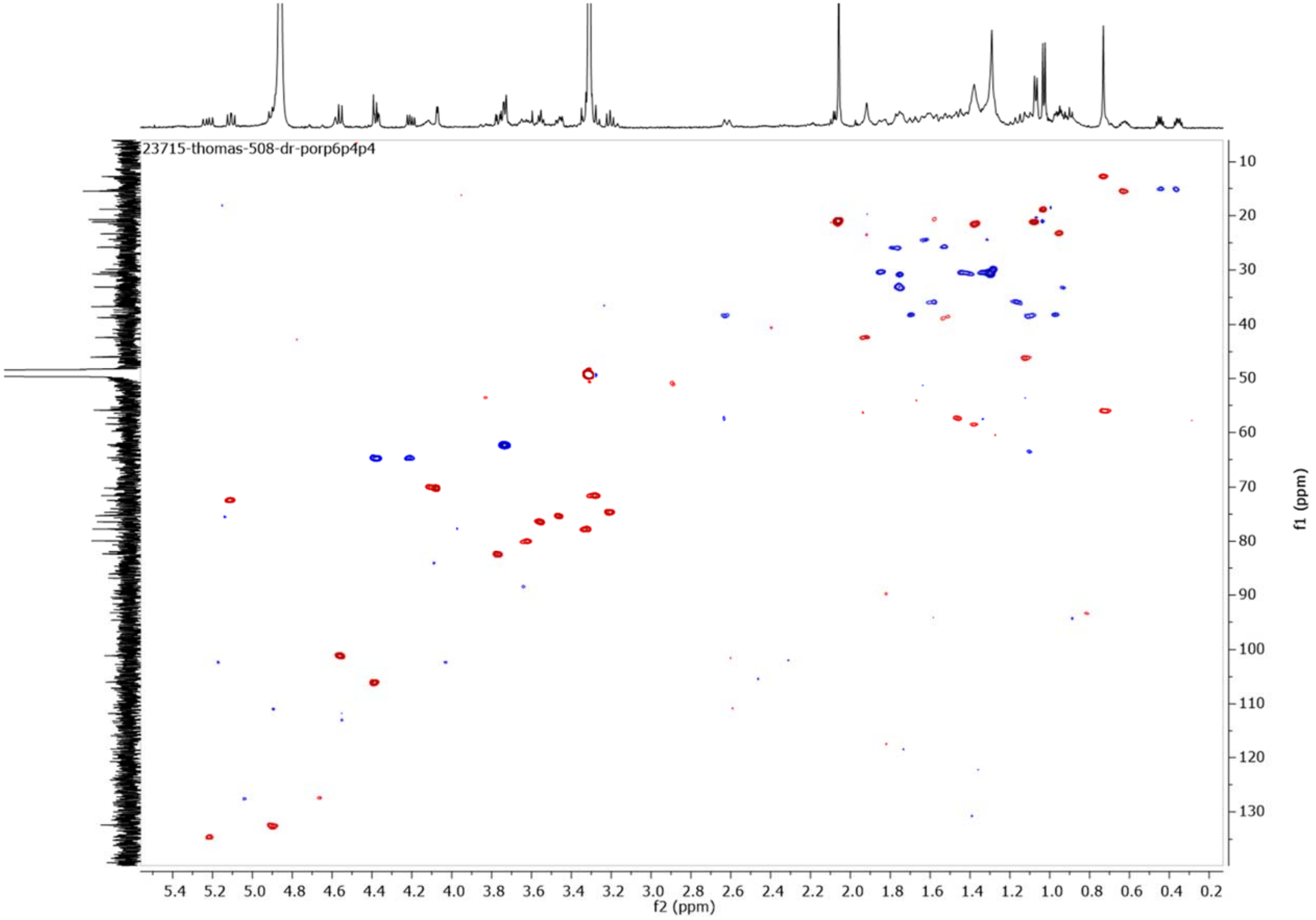

Figure S38. HSQC NMR spectrum of **5** at 500 MHz in CD_3OD

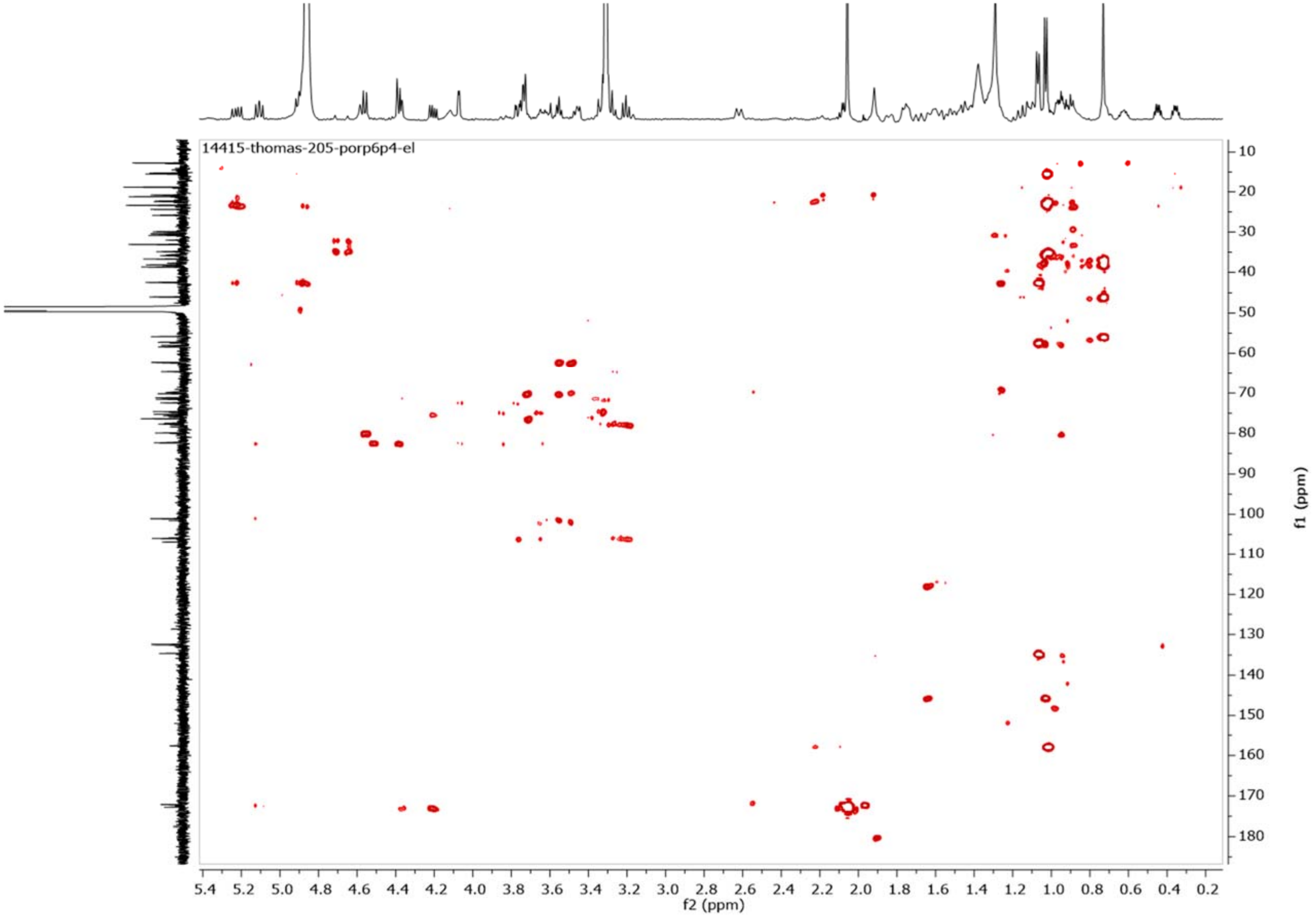

Figure S39. HMBC spectrum of **5** at 500 MHz in CD_3OD

Compound Formula	Name	RT	Algorithm
$C_{41}H_{66}O_{13}$	**Poecillastroside F**	7.024	Spectrum Extraction

MS Spectrum

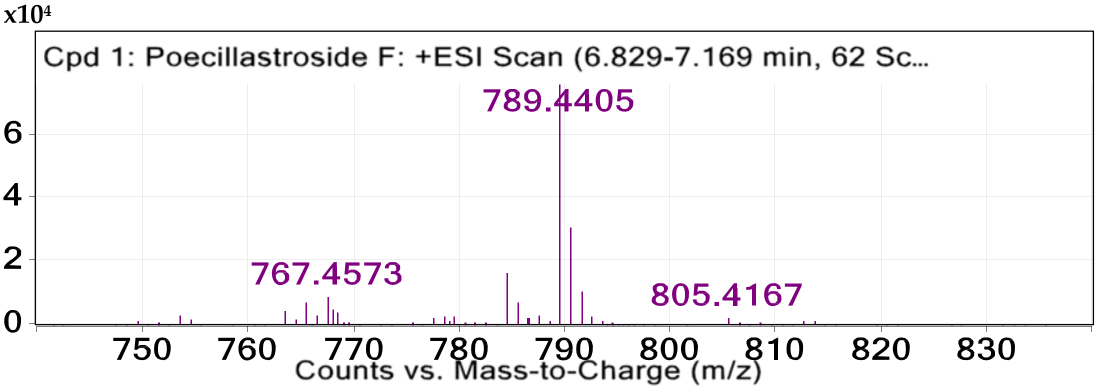

Figure S40. (+)-HRESIMS analysis of **6.**

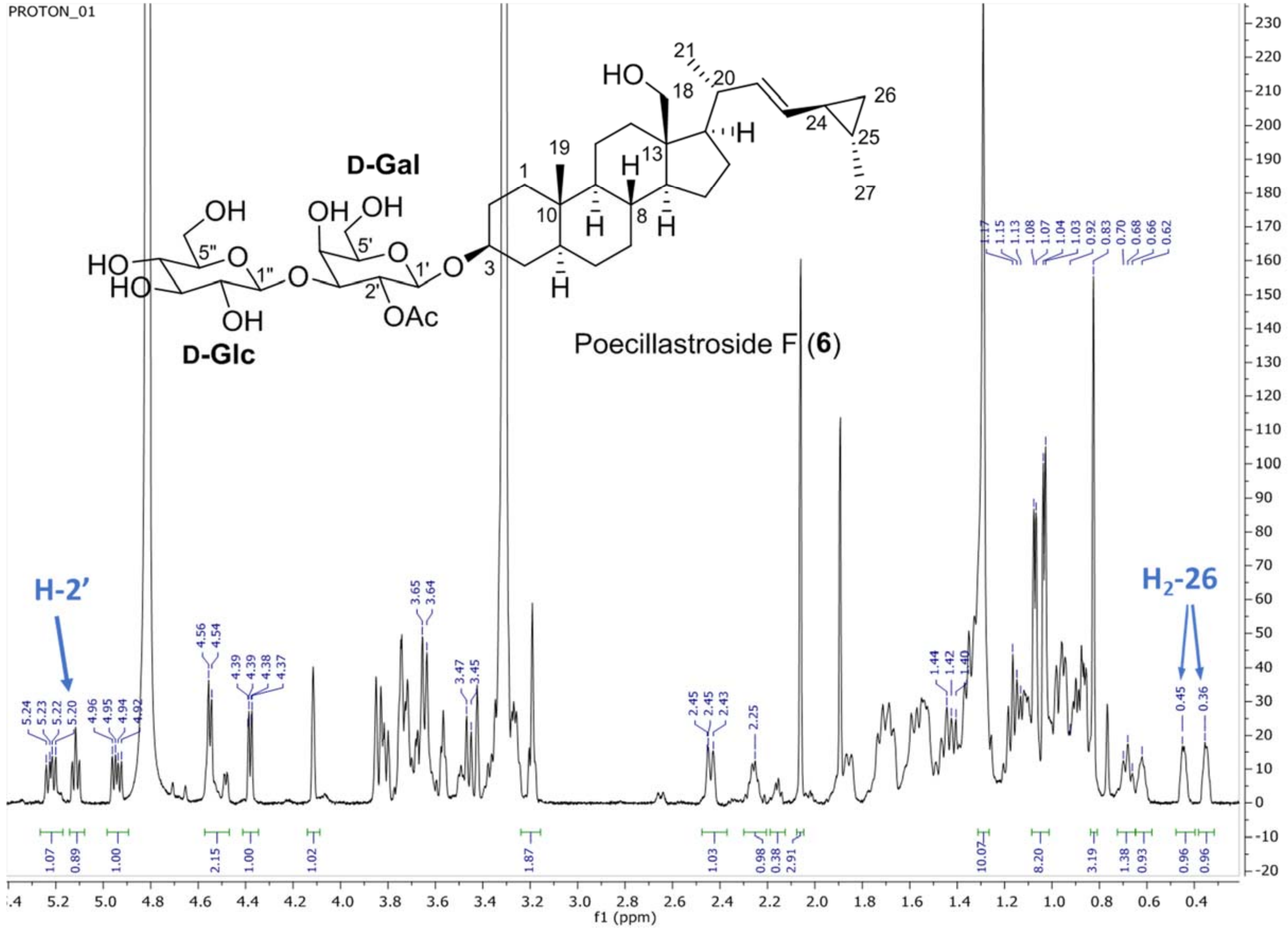

Figure S41. ^{1}H NMR spectrum of **6** at 600 MHz in CD_3OD

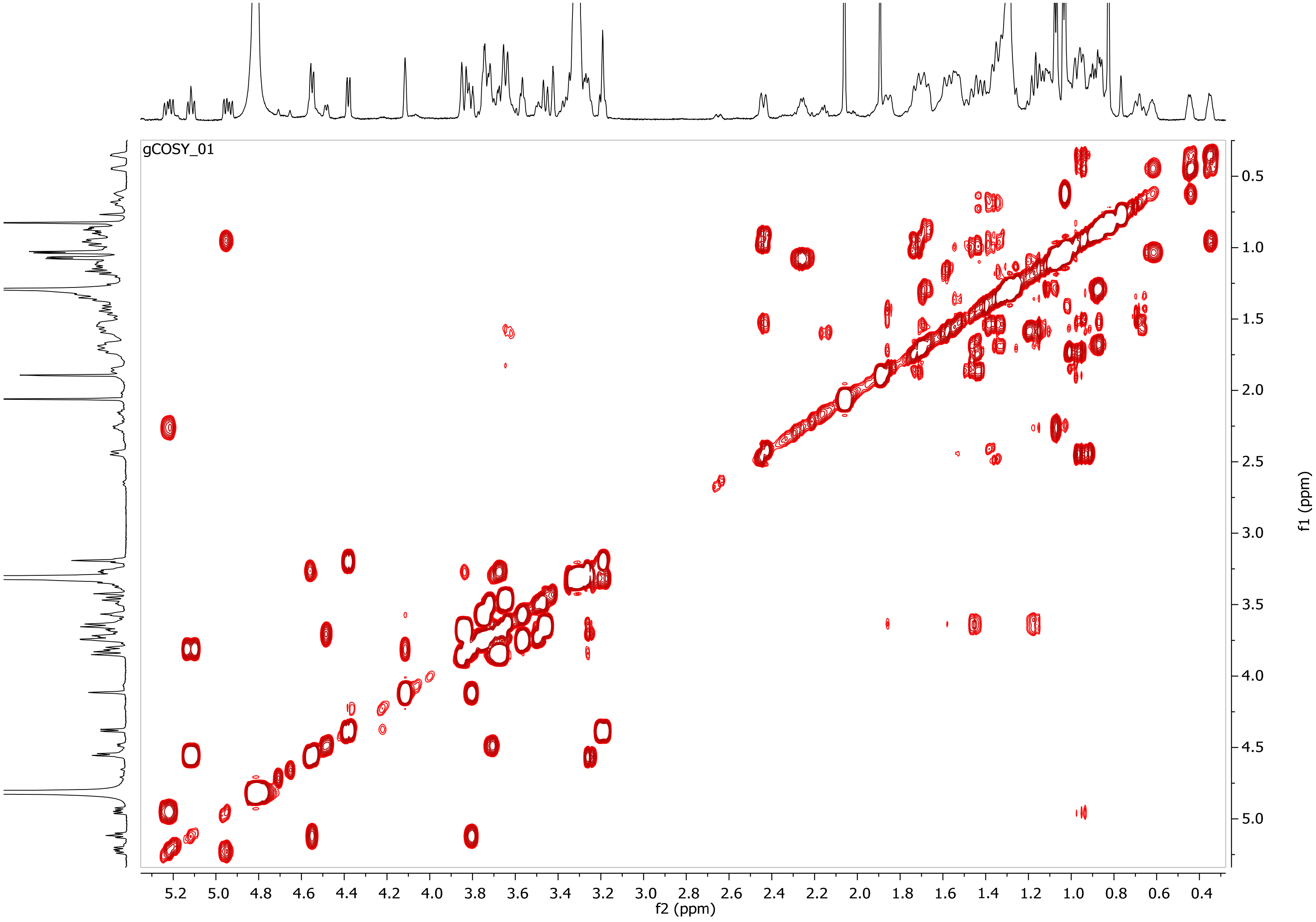

Figure S42. COSY NMR spectrum of **6** at 600 MHz in CD_3OD

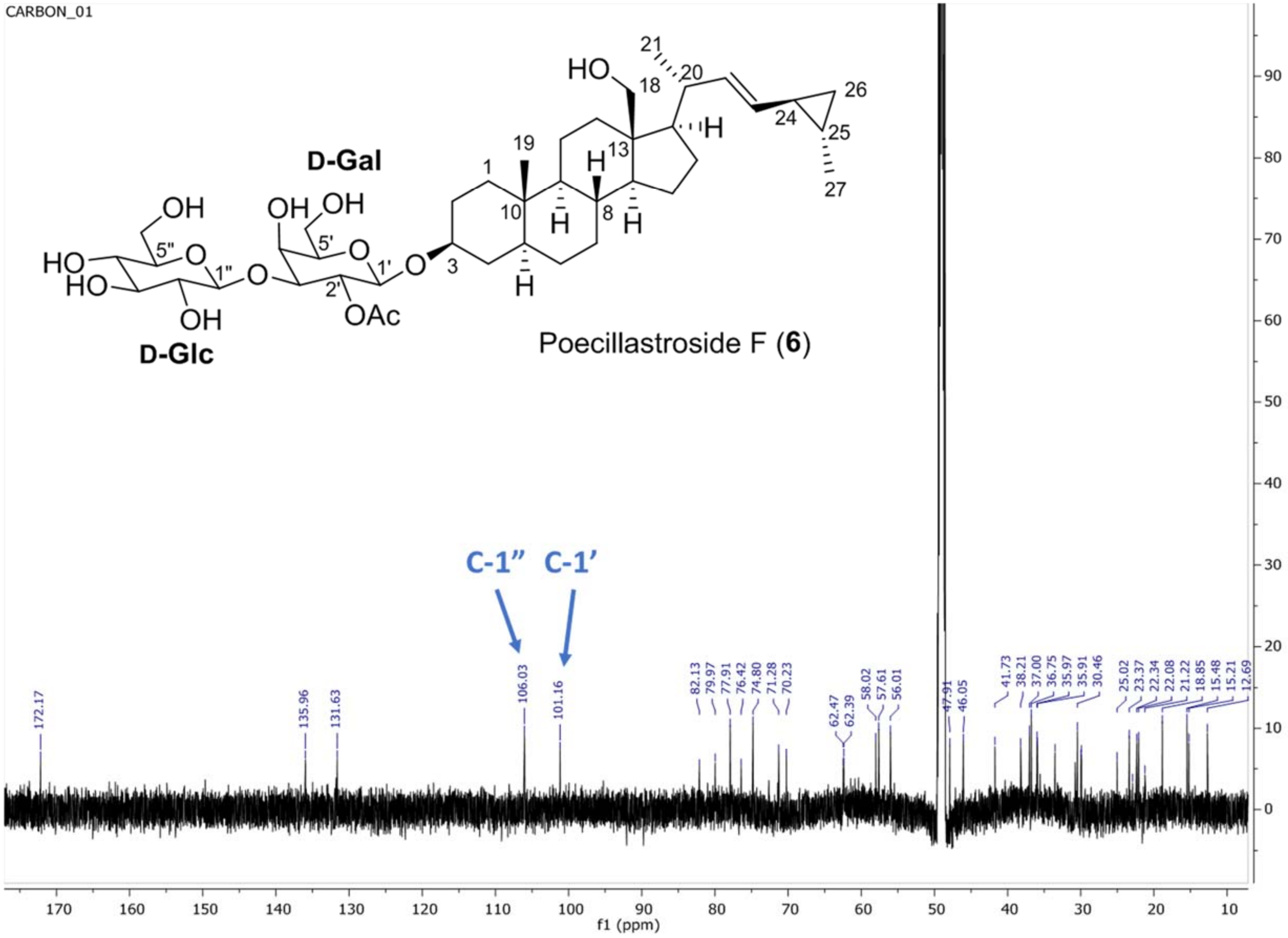

Figure S43. ^{13}C NMR spectrum of **6** at 150 MHz in CD_3OD

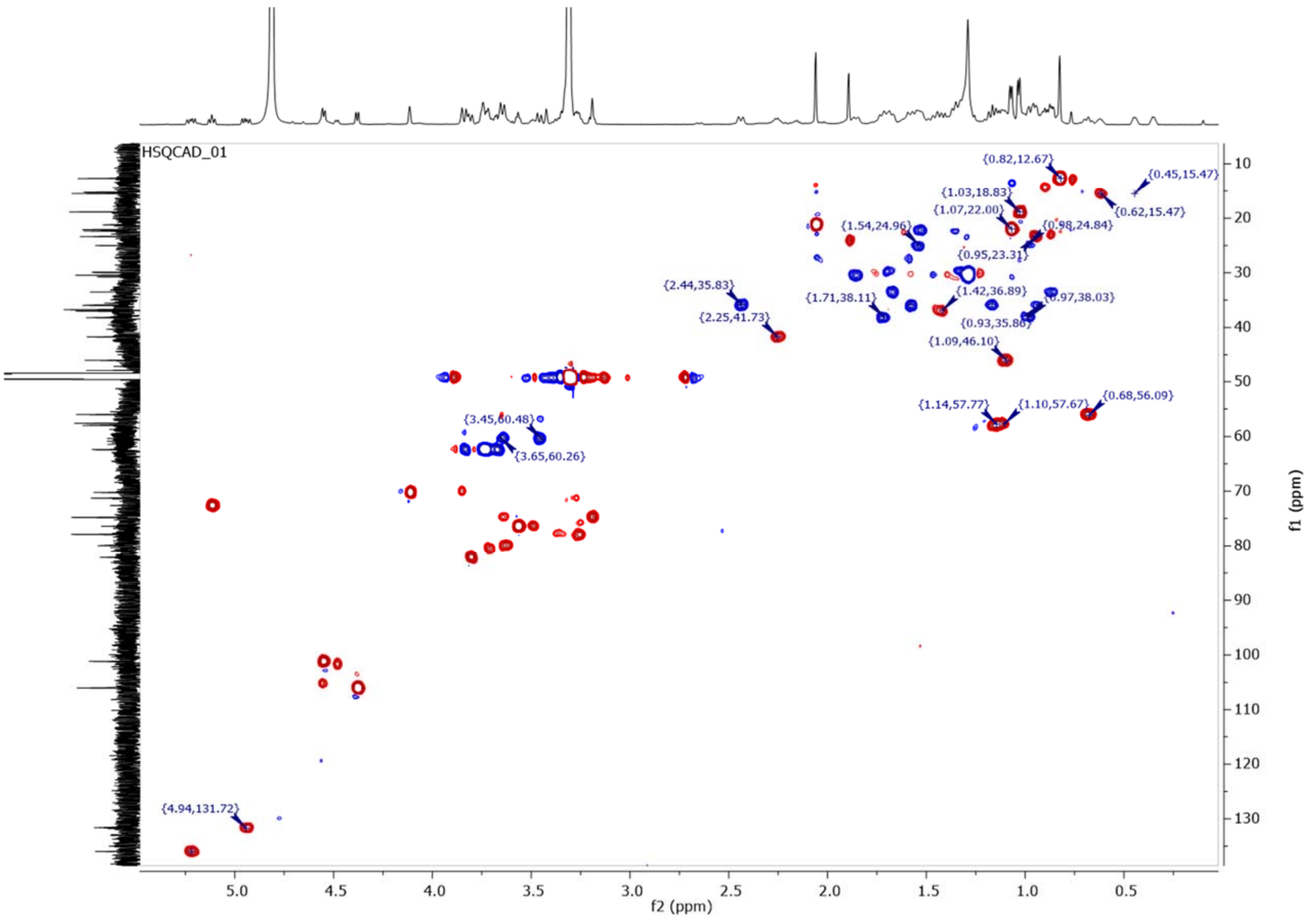

Figure S44. HSQC NMR spectrum of **6** at 600 MHz in CD_3OD

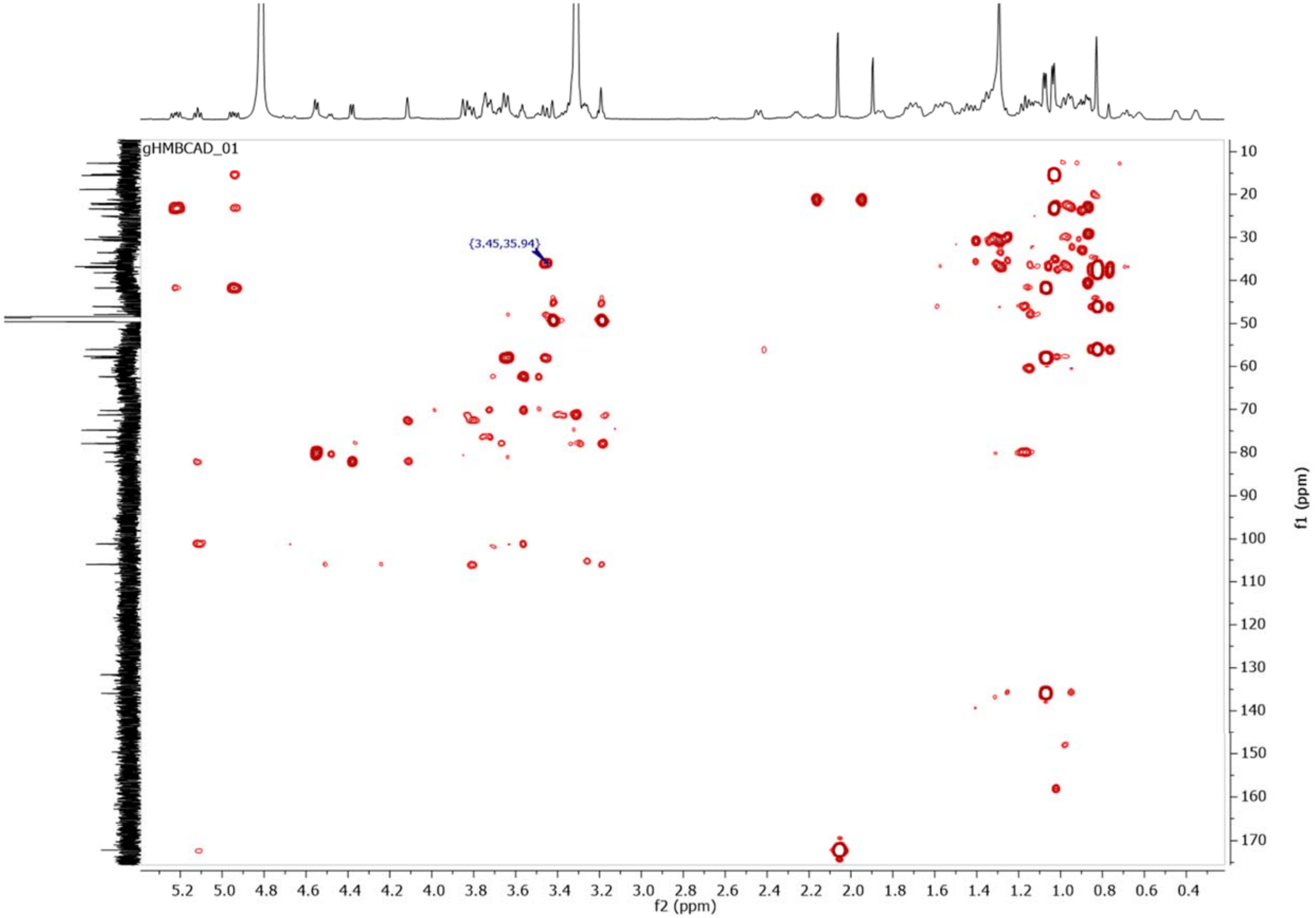

Figure S45. HMBC spectrum of **6** at 600 MHz in CD_3OD

Compound Formula	Name	RT	Algorithm
$C_{43}H_{68}O_{14}$	**Poecillastroside G**	7.384	Spectrum Extraction

MS Spectrum

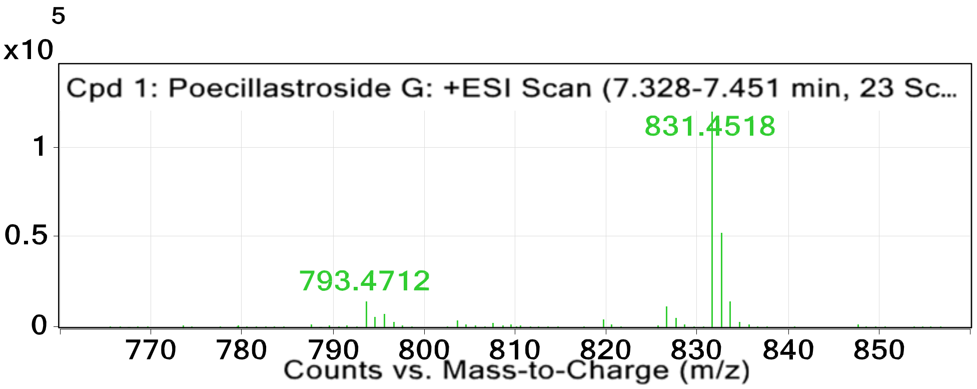

Figure S46. (+)-HRESIMS analysis of **7.**

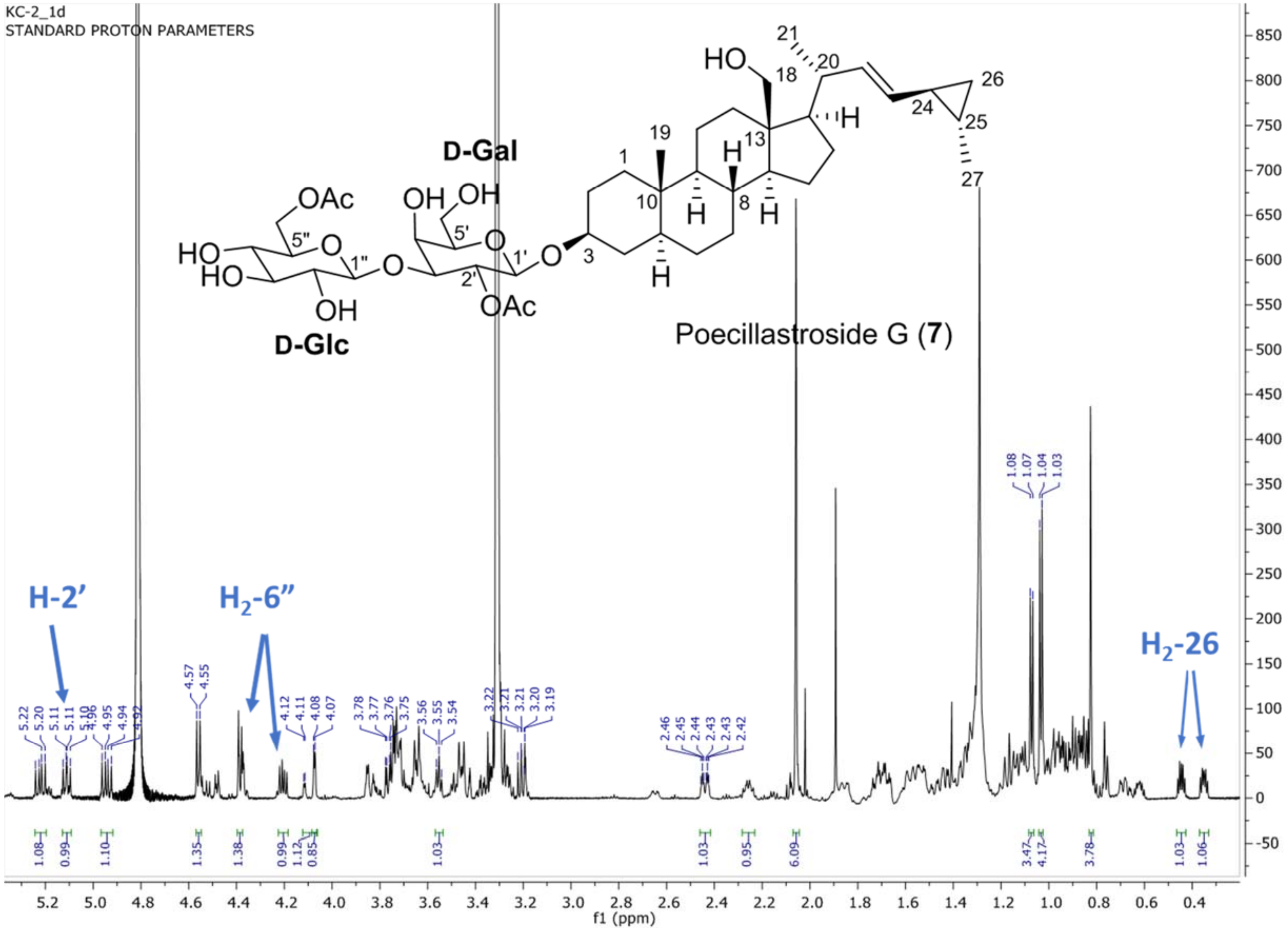

Figure S47. ^{1}H NMR spectrum of **7** at 600 MHz in CD_3OD

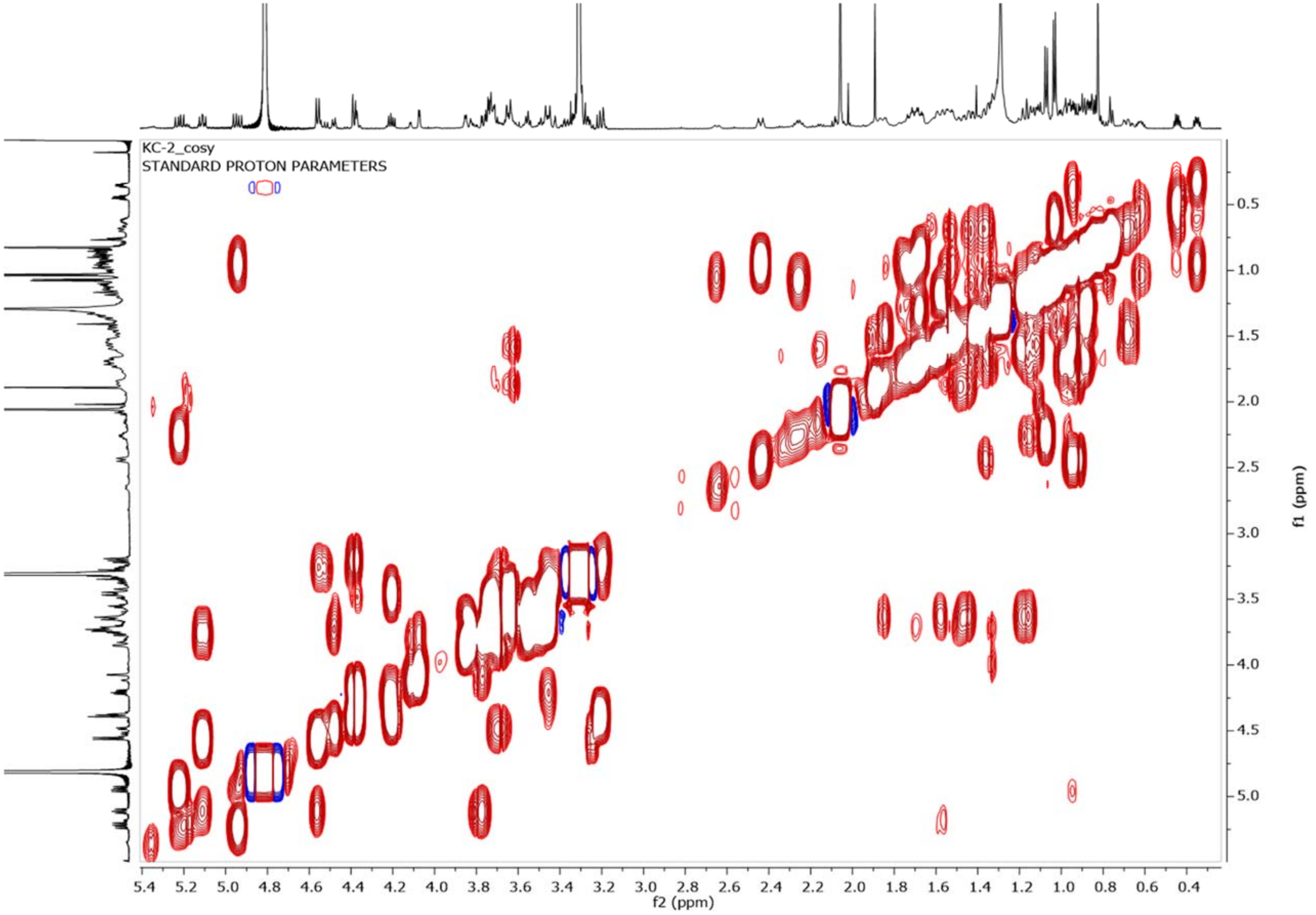

Figure S48. COSY NMR spectrum of **7** at 600 MHz in CD_3OD

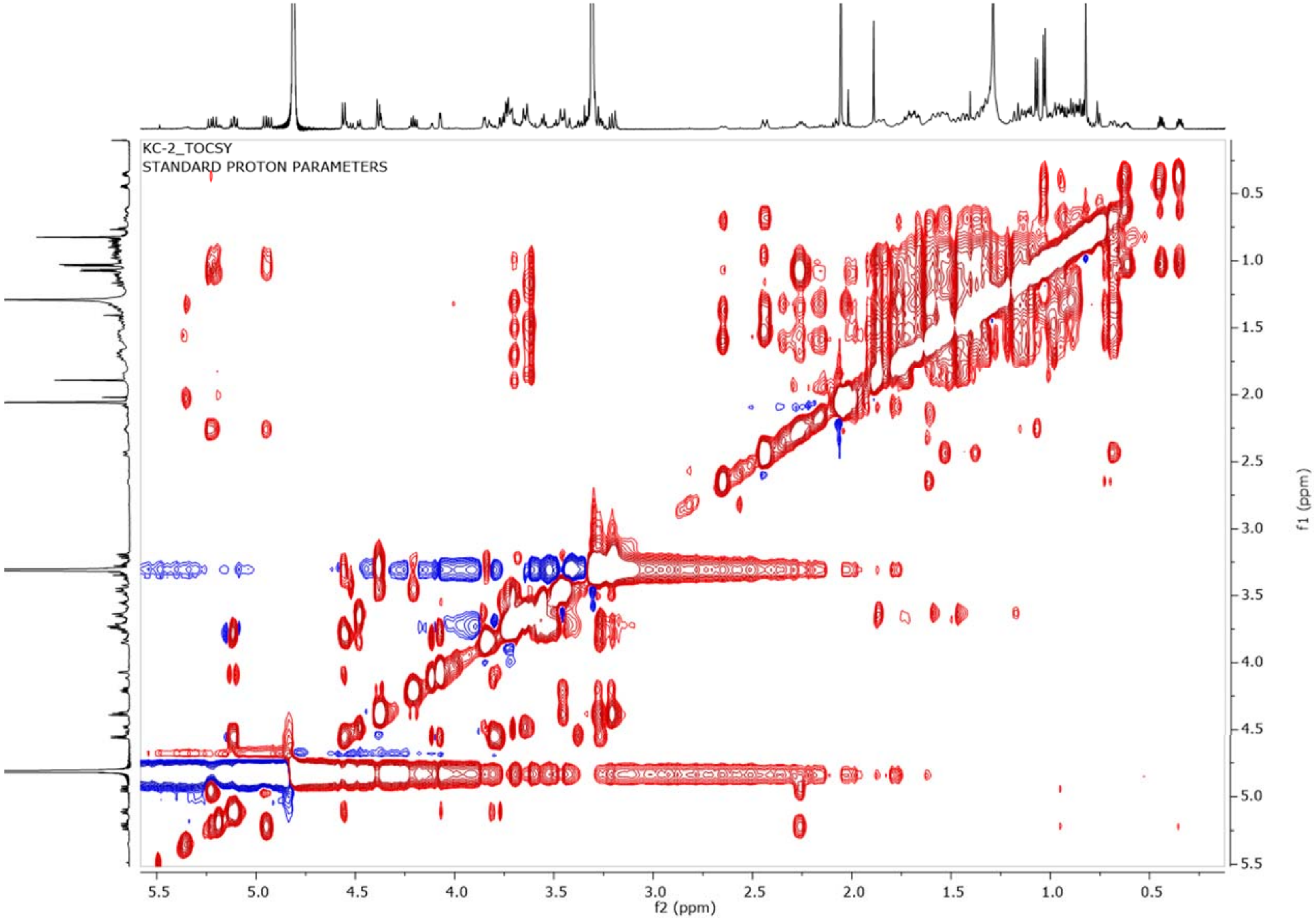

Figure S49. TOCSY NMR spectrum of **7** at 600 MHz in CD_3OD

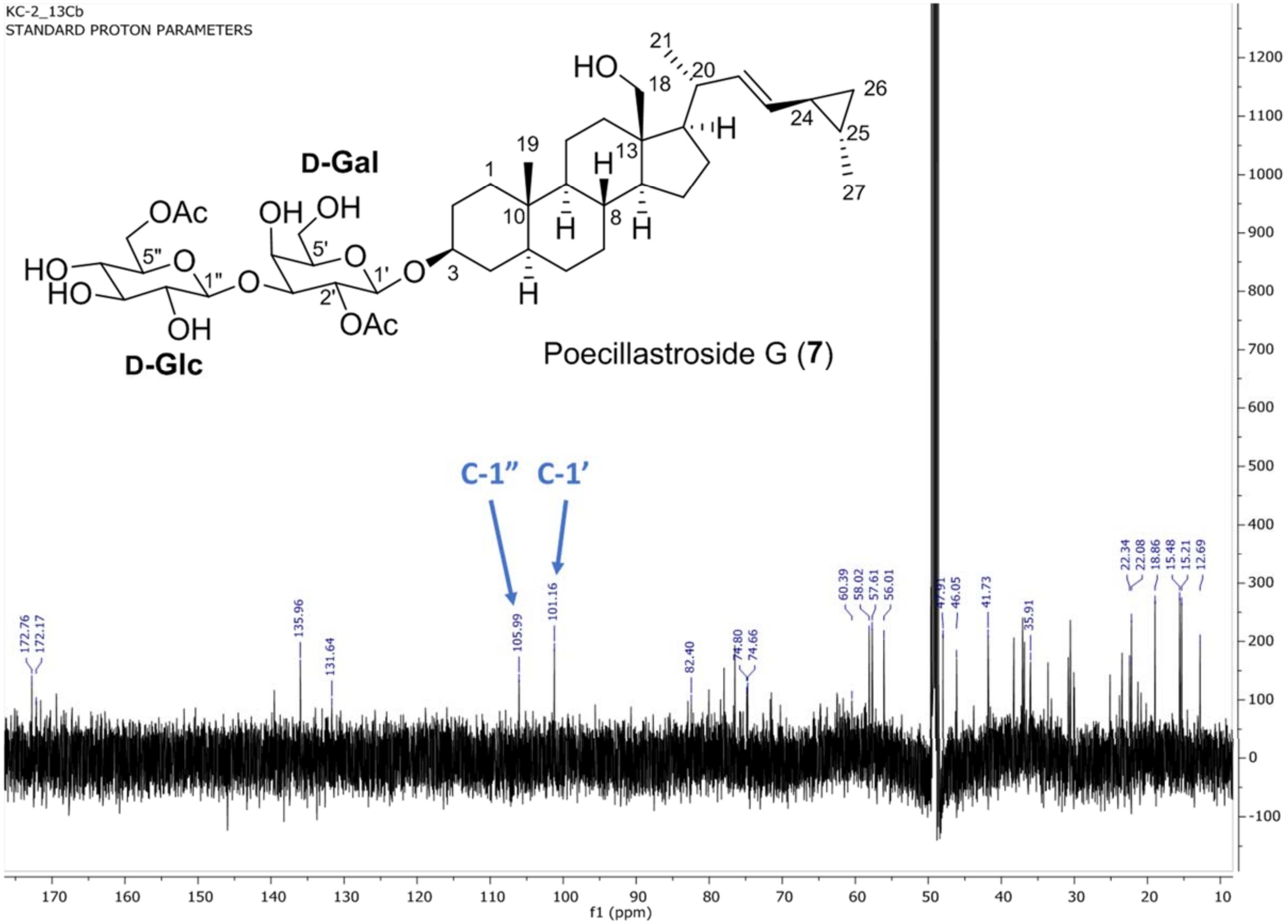

Figure S50. ^{13}C NMR spectrum of **7** at 150 MHz in CD_3OD

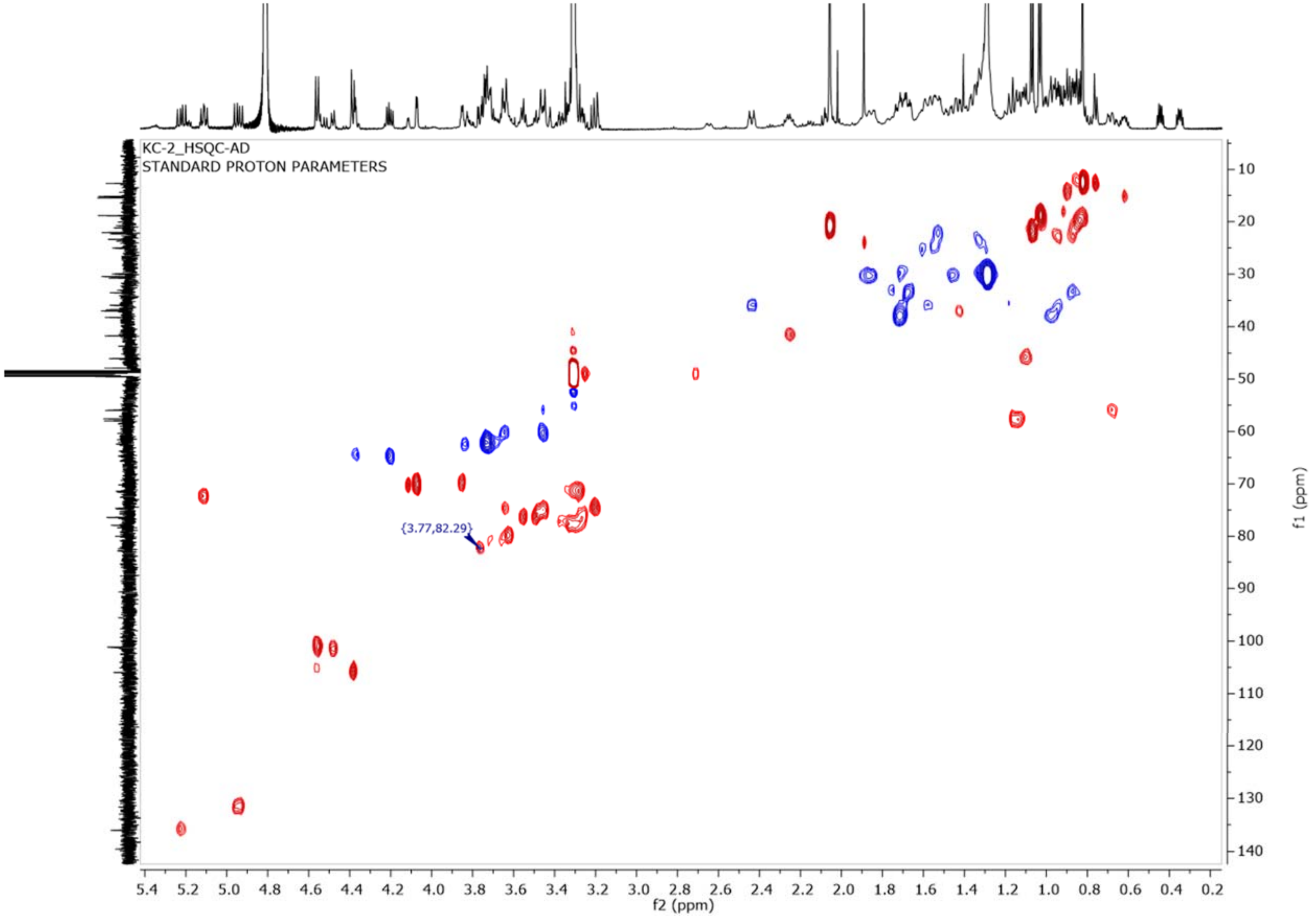

Figure S51. HSQC NMR spectrum of **7** at 600 MHz in CD_3OD

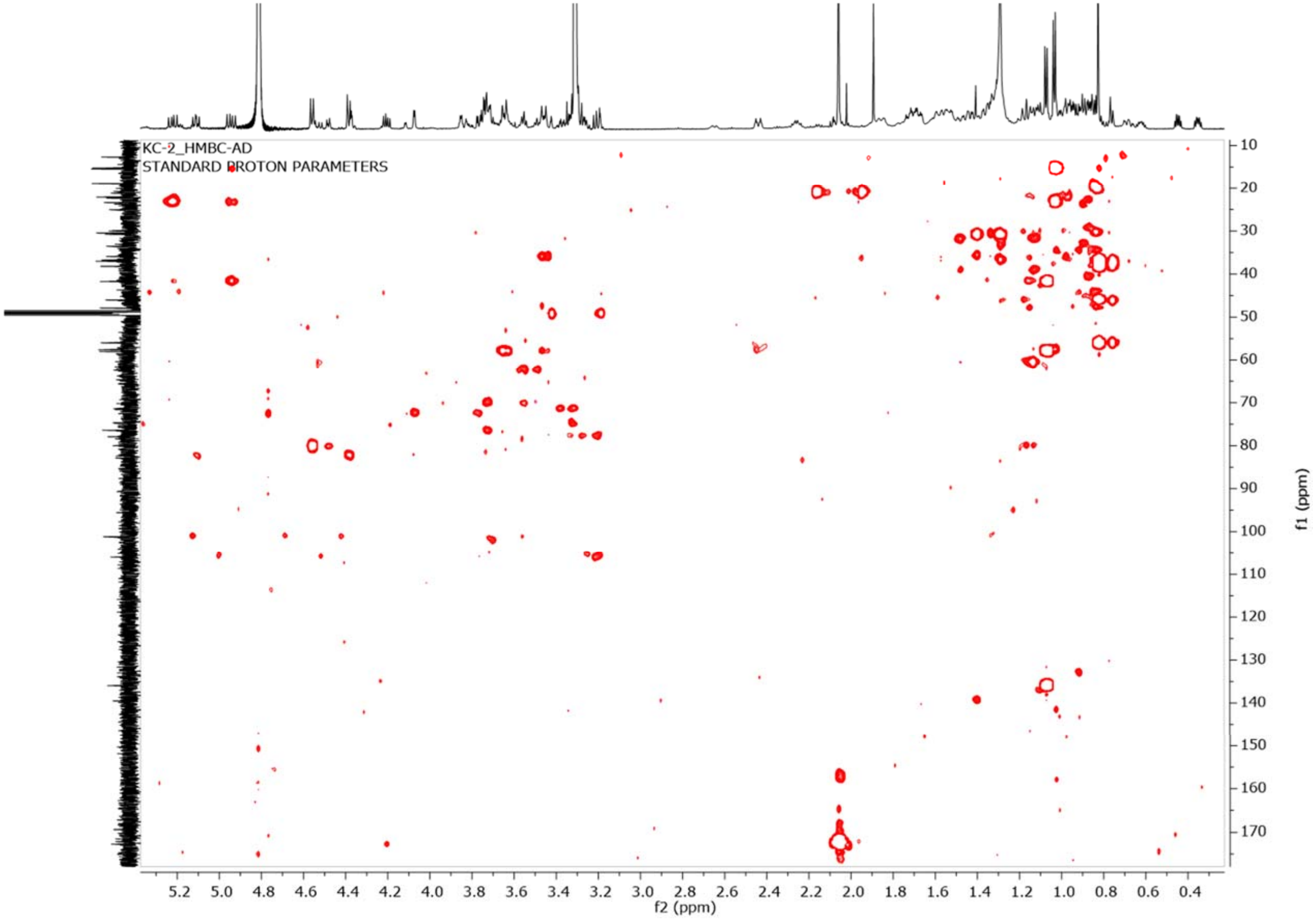

Figure S52. HMBC spectrum of **7** at 600 MHz in CD_3OD

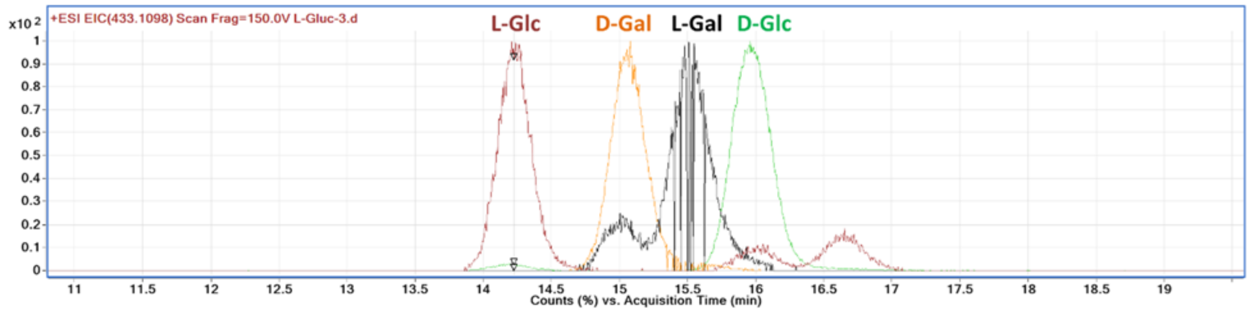

Figure S53. UPLC-qToF analysis of the four monosaccharide derivatives

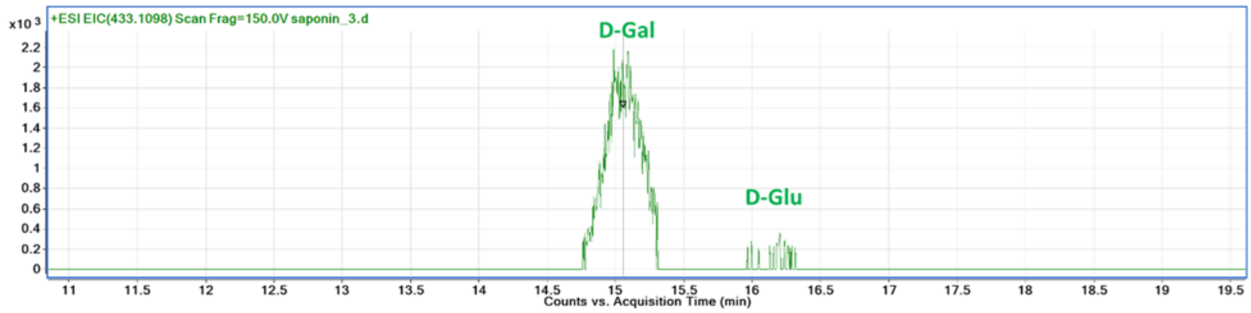

Figure S54. Absolute configuration of the pyranose moieties of **3**

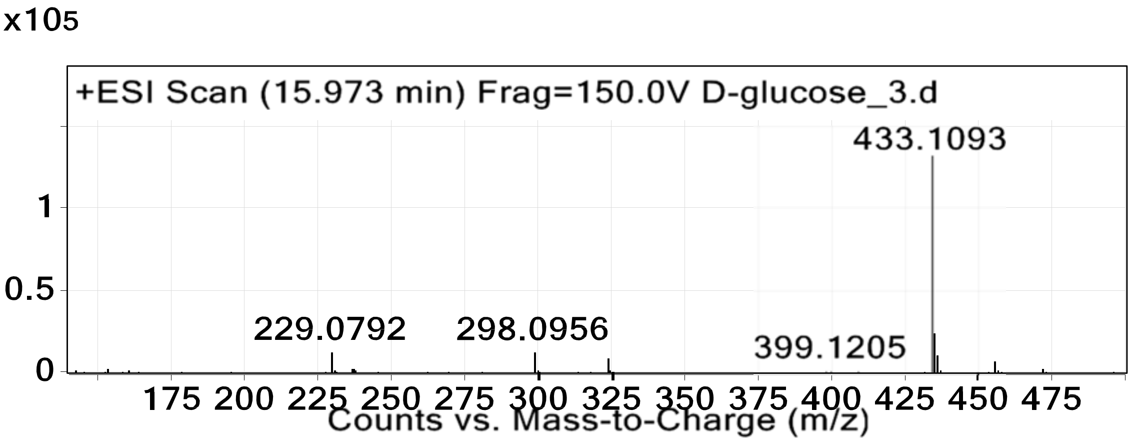

Figure S55. ESI-(+) mass spectrum of D-(+)-glucose derivative
